# Efficacy of Biologic Agents and Small Molecules for Endoscopic Improvement and Mucosal Healing in Patients with Moderate-to-Severe Ulcerative Colitis: Systematic Review and Meta-Analysis

**DOI:** 10.3390/jcm14165789

**Published:** 2025-08-15

**Authors:** Christos Mademlis, Anastasia Katsoula, Theocharis Koufakis, Paschalis Paschos, Aristeidis Kefas, Lefteris Teperikidis, Niki Theodoridou, Olga Giouleme

**Affiliations:** 1 Gastroenterology and Hepatology Unit, Second Propaedeutic Department of Internal Medicine, Aristotle University of Thessaloniki, 54124 Thessaloniki, Greece; chrismademlis97@gmail.com (C.M.); akatsoula@auth.gr (A.K.); giouleme@auth.gr (O.G.); 2 Second Propaedeutic Department of Internal Medicine, Hippokration General Hospital, Aristotle University of Thessaloniki, 54124 Thessaloniki, Greece; md.aristideskefas@yahoo.gr (A.K.); lefty12@gmail.com (L.T.); 3 Clinical Research and Evidence-Based Medicine Unit, Second Medical Department, Aristotle University of Thessaloniki, 54124 Thessaloniki, Greece; pashospas@hotmail.com; 4 Dermatology Unit of Gustave Roussy, 114 rue Édouard-Vaillant, 94805 Villejuif Cedex, France; nikitheodor2@gmail.com

**Keywords:** ulcerative colitis, biologic agents, endoscopic improvement

## Abstract

**Background and Aim**: The therapeutic landscape for ulcerative colitis (UC) is rapidly evolving, with an increasing number of biologic agents available. This systematic review and meta-analysis synthesized randomized controlled trials (RCTs) data on biologic therapies for achieving key endoscopic and histologic endpoints in moderate to severe UC. **Methods**: A systematic search of MEDLINE, EMBASE, Cochrane Library, Web of Science and grey literature was conducted through November 2024. Separate meta-analyses were performed for induction and maintenance. A random-effects model was used to estimate relative risks (RR), with 95% confidence intervals (CI), and confidence in estimates was evaluated with the GRADE approach (Grading of Recommendation Assessment, Development and Evaluation). **Results**: We included 40 RCTs (13 therapies, 14,369 patients). Thirty-two trials provided data in induction and twenty-eight in maintenance. During induction, all biologic therapies, except mirikizumab and filgotinib 100 mg, demonstrated superiority over placebo (RR 2.02, 95% CI: 1.76–2.31, I^2^ = 72%) for endoscopic improvement. Upadacitinib showed the highest efficacy (RR 5.53, 95% CI: 3.78–8.09). For mucosal healing, all interventions were superior to placebo (RR 2.95, 95% CI: 2.11–4.13, I^2^ = 61%), except filgotinib 100 mg. Risankizumab showed the highest efficacy (RR 10.25, 95% CI: 2.49–42.11). In maintenance, all therapies showed superiority over placebo for endoscopic improvement. For mucosal healing all therapies were superior to placebo, except risankizumab. Upadacitinib 30 mg showed the highest efficacy (RR 4.01, 95% CI: 1.81–8.87). **Conclusions**: Biologic and small-molecule therapies demonstrated substantial efficacy in achieving key endpoints. Standardized outcome definitions and further head-to-head RCTs are essential to strengthen confidence in our findings.

## 1. Introduction

Ulcerative colitis (UC) affects approximately 1.5 million individuals in the United States, 2.2 million individuals in Europe, and several thousand more worldwide [1] It is characterized by a chronic, relapsing-remitting disease course. This condition often results in a significant disease burden, with more than one-third of patients requiring hospitalization or surgical intervention due to complications [2]. For many patients, moderate-to-severe disease activity necessitates the use of advanced immunosuppressive therapies to manage symptoms and prevent disease progression.

UC arises from a multifactorial interplay of genetic susceptibility, epithelial barrier defects, dysbiosis, and dysregulated mucosal immunity, which distinguishes it from Crohn’s disease (CD), where Th1/Th17 cytokine signaling and tumor necrosis factor-α (TNF-α)–driven inflammation predominates. In UC, neutrophilic infiltration and aberrant Th2/Th17 cytokine signaling (including IL-5, IL-13, IL-17, and IL-22) perpetuate mucosal injury, while innate immune dysfunction amplifies barrier breakdown [3,4,5]. These mechanistic differences underpin the variable efficacy of targeted biologics and small molecules across therapeutic classes.

The current therapeutic armamentarium for moderate-to-severe UC encompasses biologic agents and small-molecules, each targeting distinct immune and inflammatory pathways. Anti-TNF monoclonal antibodies, including infliximab, adalimumab and golimumab, neutralize both soluble and membrane-bound TNF-α. By doing so, they suppress pro-inflammatory signaling, promote apoptosis of activated CD4^+^ T lymphocytes, and support an anti-inflammatory immune environment [6]. Moreover, vedolizumab is a humanized monoclonal antibody that selectively binds the gut-homing α4β7 integrin on memory T lymphocytes. By blocking its interaction with mucosal addressin cell adhesion molecule-1 (MAdCAM-1) on intestinal endothelium, vedolizumab prevents lymphocyte trafficking into the gastrointestinal mucosa [7]. The interleukin-12/23 cytokine family plays a pivotal role in driving type 1 and type 17 immune responses that perpetuate mucosal injury. Ustekinumab, a first-generation anti-IL-12/23p40 antibody, and selective IL-23p19 inhibitors—such as mirikizumab, risankizumab and guselkumab—target these pathways, aiming to suppress the inflammatory cascade in IBD [8]. Among small molecules, Janus kinase (JAK) signaling plays a central role in the pathogenesis of IBD by transmitting signals from type I and type II cytokine receptors through the JAK–STAT pathway, promoting intestinal inflammation. JAK inhibitors, including tofacitinib, filgotinib and upadacitinib, selectively block this intracellular signaling cascade, attenuating both innate and adaptive immune activation in IBD [9]. Sphingosine-1-phosphate (S1P) signaling regulates the egress of T and B lymphocytes from lymph nodes into the circulation. By selectively modulating S1P receptors, agents such as ozanimod and etrasimod sequester lymphocytes within lymphoid tissues, thereby reducing their migration to the gut and dampening chronic intestinal inflammation [10]. These mechanistically diverse therapies reflect the complex immunopathogenesis of ulcerative colitis and form the rationale for evaluating their relative efficacy on disease progression.

The therapeutic goals in the management of UC have evolved significantly over the past decades. While the primary aim was once to relieve symptoms, the focus has now shifted toward achieving more comprehensive outcomes such as endoscopic improvement and mucosal healing. The latest STRIDE II (Selecting Therapeutic Targets in Inflammatory Bowel Disease) recommendation highlights endoscopic healing as the principal long-term goal because persistent mucosal inflammation predicts complications and relapse. Histologic remission, although not a formal target, is recognized as an adjunct indicator of deeper disease control, particularly in ulcerative colitis [11]. Furthermore, data demonstrate that achieving endoscopic remission has been consistently associated with improved long-term outcomes, including reduced rates of colectomy, hospitalizations, and the need for immunosuppressive therapy in patients with ulcerative colitis [12,13,14,15]. Moreover, maintaining endoscopic improvement has been associated with a lower risk of developing colorectal neoplasia, including colorectal cancer, in patients with IBD [16]. Notably, persistent endoscopic activity despite clinical remission is a strong predictor of early relapse, underscoring the importance of objective disease assessment beyond symptomatic control [13,15]. International guidelines now advocate for the routine use of endoscopic and histologic endpoints in both clinical practice and trials, reflecting a shift from symptom-based management to a treat-to-target approach [17,18]. These findings underscore the importance of redefining treatment success beyond symptomatic relief to incorporate objective measures of disease activity. Despite the critical importance of these therapeutic targets, substantial debate still persists regarding their optimal definitions. A significant challenge lies in the inconsistent reporting and lack of standardized criteria for defining endoscopic, histologic, and mucosal outcomes across studies. Bridging this knowledge gap necessitates a systematic effort to establish clear, uniform definitions so as to assess the efficacy of various therapeutic classes.

To this end, we conducted a systematic review and meta-analysis of randomized controlled trials (RCTs) evaluating the efficacy of therapies for moderate-to-severe UC. Our primary objective was to synthesize the available clinical trial results by integrating the various terms used to define endoscopic, histologic, and mucosal outcomes and subsequently to evaluate the ability of these therapies to induce and maintain these key outcomes. This work aims to provide critical insights into the relative effectiveness of these agents and to inform clinical decision-making in UC management.

## 2. Methods

### 2.1. Search Strategy and Selection Criteria

This systematic review and meta-analysis was conducted by the use of an a priori established protocol and is reported according to the Preferred Reporting Items for Systematic Reviews and Meta-Analyses (PRISMA) statement for systematic reviews incorporating meta-analyses for healthcare interventions [19] (Supplementary Table S4). A comprehensive search of MEDLINE, Embase, Cochrane Library, and Web of Science was conducted by two authors (CM and AK) up to November 2024. Additional searches were also performed manually in ClinicalTrials.gov and major gastroenterology conference abstracts (European Crohn’s and Colitis Organization, Digestive Disease Week, and United European Gastroenterology Week) from 2018 to 2024 to capture unpublished or gray literature. The full search strategy used for each database is presented in the Supplementary Materials under the section ‘Search Algorithm’.

Eligible studies included phase 2 and 3 RCTs evaluating the efficacy and safety of biologics or small molecule drugs as induction or maintenance therapy in patients with moderate-to-severe UC. Inclusion criteria were adult patients (≥18 years) diagnosed with moderate-to-severe UC using standardized scoring methods (defined as a Mayo Score of 6–12, with an endoscopic sub-score of 2–3) who were either biologic-naive or had previously been exposed to at least one biologic factor, a minimum follow-up of six weeks and reporting of at least one predefined endoscopic or histologic outcome. Studies were excluded if they assessed Crohn’s disease, indeterminate colitis, malignancy, extensive colectomy history, non-English full texts, or unapproved treatments.

For clinical trials evaluating multiple doses of a biological agent, all treatment arms receiving the drug were included in the analysis, provided that the administered doses were consistent with standard clinical practice. However, in cases where one of the doses was not commonly used in routine clinical practice or did not align with regulatory-approved regimens, only data from the approved dosing schedules by the US Food and Drug Administration (FDA) and the European Medicines Agency (EMA) were considered.

Two independent reviewers (CM and AK) screened the titles and abstracts of the identified records, excluding those that were clearly irrelevant. The full texts of potentially eligible studies were then assessed to determine their relevance to the research topic. Additionally, the reference lists of the included studies, as well as those from relevant systematic reviews and meta-analyses, were manually examined to identify any additional publications. Any disagreements regarding study selection were resolved through discussion with a third reviewer (PP). Data extraction from the selected studies was conducted by two independent reviewers (CM and AK).

Data from each study were extracted independently using a pre-designed Microsoft Excel form (version 16.9). The extracted information included citation details, the last name of the first author, study design, sample size, study duration, population characteristics, exposure details (such as drug type, dosage, and treatment duration), and reported outcomes. Any discrepancies in data extraction were resolved through consensus, with reference to the original study. To assess study quality, three independent reviewers evaluated the included trials using the Cochrane Risk of Bias Tool (version 2.0).

### 2.2. Outcome Definitions

The primary outcomes included endoscopic improvement, defined as a Mayo Endoscopic Subscore (MES) of ≤1, and mucosal healing, which was defined as the concurrent achievement of endoscopic and histologic endpoints. The Mayo Endoscopic Subscore (MES) is a four-point scale assessing endoscopic disease activity in ulcerative colitis. A score of 0 reflects normal or inactive mucosa, 1 indicates mild disease with erythema and mild friability, 2 corresponds to moderate disease with marked erythema, loss of vascular pattern and friability, or superficial erosions, and 3 denotes severe disease characterized by spontaneous bleeding or ulcerations [20]. Histologic activity was most commonly assessed using the Geboes Score, which grades ulcerative colitis inflammation from structural changes to ulceration, with scores below 2 indicating histologic remission and scores below 3.1 indicating histologic improvement. Additional indices used included the Robarts Histopathology Index (RHI), which quantifies neutrophilic infiltration and epithelial injury on a 0–33 scale, and the Nancy Index (NI), which grades chronic and acute inflammatory infiltrates and ulceration on a 0–4 scale. All three indices provide validated measures of mucosal inflammation suitable for defining histologic endpoints in clinical trials [21]. Due to the considerable heterogeneity among studies regarding the definition of mucosal healing, our systematic review and meta-analysis adopted a standardized definition for mucosal healing; encompassing endoscopic improvement (defined as a [MES] ≤ 1) and histologic remission (defined as a Geboes scoring system < 2), endoscopic improvement and histologic improvement (defined as as a Geboes score < 3.1 or a Robarts Histopathology Index (RHI) ≤ 3) and lastly endoscopic remission (defined as MES = 0) and histologic remission. In studies evaluating more than one of these definitions, mucosal healing was defined as the simultaneous achievement of both endoscopic remission and histologic remission. Secondary outcomes included the achievement of endoscopic remission, defined as a (MES) of 0 or a Baron score of 0, histologic improvement defined as a Geboes score < 3.1, and histologic remission defined as a Geboes score < 2.

### 2.3. Data Analysis

Pairwise meta-analyses were conducted to compare the efficacy of biologic agents and small molecule drugs for the induction therapy of moderate-to-severe ulcerative colitis. Due to variations in study design for maintenance phase—where some trials followed a treat-straight-through approach while others used re-randomized only responders design from induction therapy—separate meta-analyses were performed for these distinct trial designs. Additional pairwise meta-analyses were conducted to assess endoscopic improvement and mucosal healing during induction and maintenance therapy in both biologic-naive/no previous biologic failure status and biologic-exposed/with previous biologic failure status populations.

Meta-analyses were performed using the “meta” and “metafor” packages in R (version 4.3.1). Pooled estimates were calculated using a random-effects model (DerSimonian and Laird method), given expected inter-study heterogeneity. Risk ratios (RRs) were used for dichotomous outcomes, with log transformation applied for normal distribution. Efficacy was assessed based on pooled RRs for each therapeutic agent compared to placebo or active comparators. Heterogeneity was assessed via Cochrane’s Q statistic and I^2^ index (>50% indicating substantial heterogeneity). Heterogeneity was managed through random-effects modeling, and head-to-head comparisons were included where available.

The Grading of Recommendations Assessment, Development, and Evaluation (GRADE) framework was employed to assess the confidence in the estimates obtained from our meta-analysis. This evaluation considered five key domains: risk of bias, inconsistency, indirectness, imprecision, and publication bias. The GRADE assessment was applied only to the primary outcomes of our meta-analysis. This systematic review and meta-analysis were conducted following a pre-established protocol and registered in the PROSPERO database (CRD42024533059).

## 3. Results

**Figure 1:**
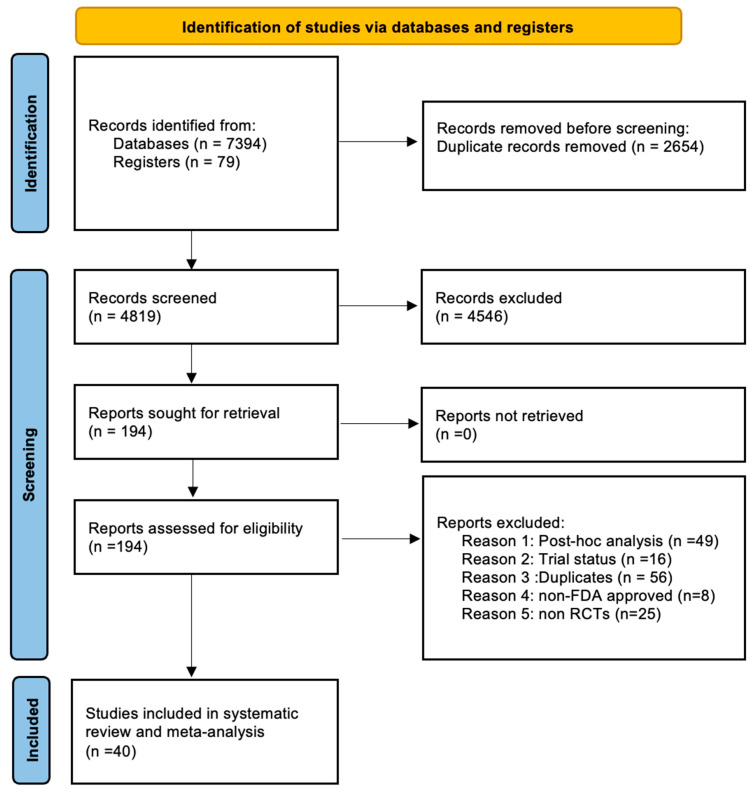
Study selection RCTs = Randomized Controlled Trials.

Our bibliographical search yielded 7473 records, of which 40 studies that fulfilled our inclusion criteria were included (Figure 1). The trials were conducted across an extensive international network of study sites. Most patients were enrolled in North America and Europe, and a substantial proportion came from Asia through both multinational trials and single-country studies in Japan and China. Patients were also recruited from South America, Australia, New Zealand, and, to a smaller extent, from Africa, creating a study population that reflects the global distribution of moderate-to-severe UC. Of these 40 clinical trials, 32 assessed induction therapy with either a biologic or a small molecule drug, comprising a total of 14,369 patients with ulcerative colitis. Among 32 induction-phase trials, 30 studies [22,23,24,25,26,27,28,29,30,31,32,33,34,35,36,37,38,39,40,41,42,43,44,45,46,47,48] assessed endoscopic improvement as an endpoint, 16 trials evaluated endoscopic remission [30,32,34,38,39,40,44,45,46,47,48,49,50], 5 assessed histologic improvement [29,39,40,41], 5 evaluated histologic remission [29,34,36,44,48], and 12 evaluated mucosal healing [30,32,35,37,40,44,45,47,48]. The main characteristics of the included trials evaluating the induction phase are described in the appendix (Supplementary Table S1). Among 28 studies evaluating maintenance therapy, 13 were conducted by the use of a treat-straight-through strategy (3473 patients) and 15 followed a randomized responders design (7738 patients). Complete data were available for 14 of the re-randomization studies, as one study [32] only reported induction-phase data due to its ongoing status. Across the re-randomization studies, 13 assessed endoscopic improvement [35,37,38,40,41,42,43,46,47,48,51,52], 6 endoscopic remission [34,38,40,46,47,48], 2 histologic improvement [41,46], 3 histologic remission [34,46,48], and 7 evaluated mucosal healing [35,37,40,46,47,48,53]. In the 13 treat-straight-through studies, all evaluated endoscopic improvement [22,24,25,26,27,28,30,31,36,44,54,55], 3 mucosal healing [30,31,44], and 2 assessed endoscopic remission, 1 histologic improvement, and 3 trials evaluated histologic remission. For the maintenance phase, the characteristics of the included trials are also displayed in the appendix (Supplementary Table S2).

The definition of endoscopic improvement was consistent across most studies, with the Mayo Endoscopic Score (MES) ≤ 1 being the standard measure. The trials evaluating adalimumab, infliximab, golimumab, ozanimod [36], tofacitinib, and vedolizumab [42,43] used the term mucosal healing, which was previously used to describe endoscopic improvement. All the trials evaluating endoscopic remission as a secondary outcome used the definition of MES = 0, except for the trial of Probert et al. [49], which used the Baron score for the definition of endoscopic outcomes [56]. For histologic improvement, definitions varied across the included studies, with the most frequently used criterion being the Geboes scoring system < 3.1. Two trials [39,40] used histologic improvement as any decrease in Geboes score, while the one trial [41] used with clarity the definition of this outcome without using a threshold value. Additionally, the LIBERTY-UC [53] study used the Robarts Histopathology Index for pathology assessment, and the VARSITY [55] study set a cutoff value of Geboes score < 3.2 for histologic improvement and incorporated both the Geboes and Robarts indices to present its results. Similar variability was observed in the definition of mucosal healing. While many studies defined mucosal healing as the simultaneous achievement of endoscopic improvement and histologic remission [30,31,32,37,48], notable deviations were observed. Some phase 3 studies [34,40,44,46,47] defined mucosal healing as the concurrent achievement of endoscopic remission (MES = 0) and histologic remission (Geboes < 2) while some phase 2b studies [35,53] also diverged by defining mucosal healing as the combination of endoscopic and histologic improvement, using the Geboes score or Robarts Histopathology Index for histologic improvement.

The included studies evaluated 13 trials on TNF antagonists (7 on infliximab [25,26,27,28,49,53], 3 trials on adalimumab [22,23,24], 3 trials on golimumab [33,51,54]), 4 trials on integrin inhibitors (vedolizumab [42,43,52,55]), 8 trials on IL-12/23 or IL-23 inhibitors (ustekinumab [41], mirikizumab [34,35], guselkumab [45,46], and risankizumab [47]), 7 trials on S1P modulators (ozanimod [36,37] and etrasimod [29,30,31,32]), 7 trials on JAK inhibitors (upadacitinib [39,40], tofacitinib [38,50], and filgotinib [48]), and 1 trial on the combination of golimumab and guselkumab [44]. Additional data from studies investigating subcutaneous formulations of infliximab and vedolizumab were included to evaluate their efficacy. Phase 3 trials of etrolizumab were excluded from the primary analysis as the therapy has not been approved in North America or Europe. A risk of bias assessment showed a low risk of bias for most of the included studies (Supplementary Figures S1 and S2).

**Figure 2:**
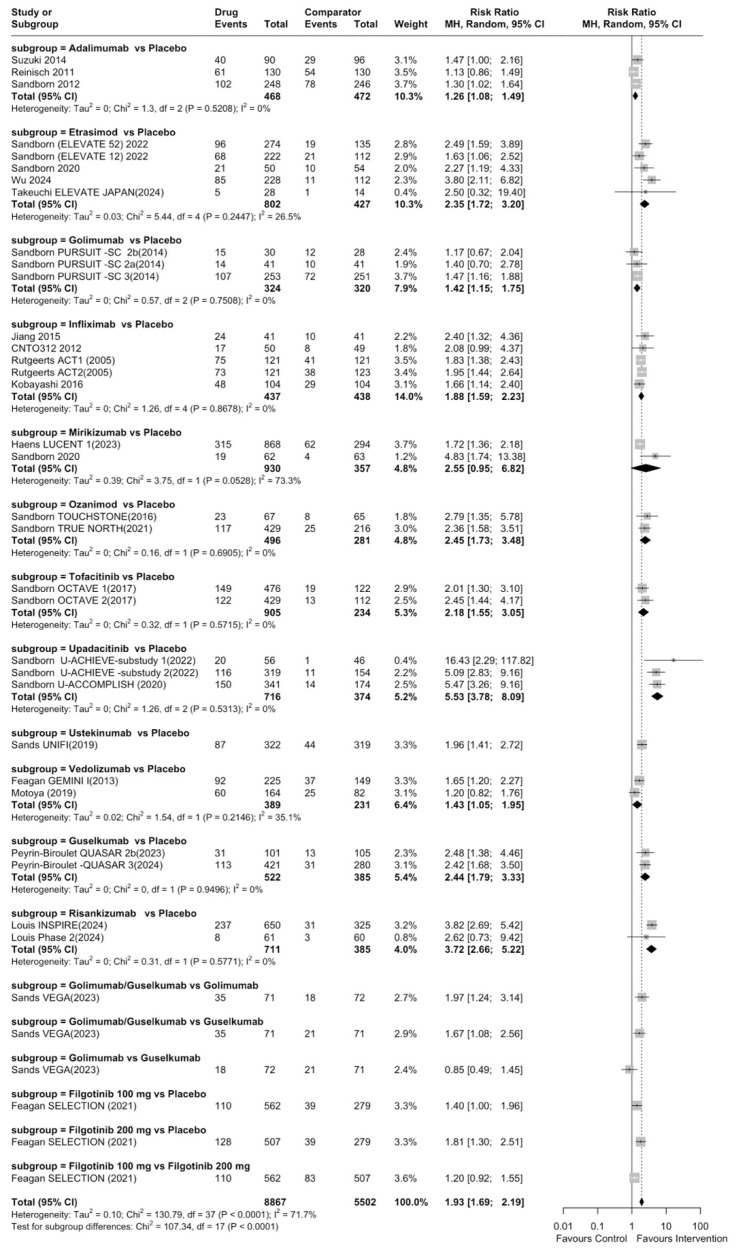
Pairwise meta-analysis for induction of endoscopic improvement.

In the evaluation of endoscopic improvement in the induction phase, a total of 30 studies were included in the meta-analysis. All interventions were significantly superior compared to placebo (RR 2.02, 95% CI: 1.76–2.31), except for mirikizumab and filgotinib 100 mg (Supplementary Figure S3). Upadacitinib showed the highest efficacy (RR 5.53, 95% CI: 3.78–8.09), and the overall heterogeneity was substantial (I^2^ = 72%) (Figure 2). To aid interpretation, the induction results are summarized in Table 1, which uses simplified layouts and an added ‘Category’ column to indicate the relative magnitude of each therapy’s effect versus placebo. Categories were defined as follows: highly efficacious (RR > 2.0); moderately efficacious (1.0 < RR ≤ 2.0 with the 95% CI excluding 1); and not efficacious (RR ≤ 1.0 or the 95% CI includes 1). These thresholds are descriptive aids to improve clarity and have not been validated as clinical cut-points; they should be interpreted cautiously. They do not represent a formal ranking of active therapies, which would require a network meta-analysis. Forest plots are retained as the primary displays for inference. The quality of evidence was low due to inconsistency and publication bias (Supplementary Table S3). For the induction of endoscopic improvement in biologic-naive patients and in patients with no previous biologic failure status, all interventions, except for mirikizumab, filgotinib 100 mg, and vedolizumab, were significantly superior to placebo in our analysis (I^2^ = 79%). Upadacitinib showed the highest efficacy among therapies (RR 4.07, 95% CI: 2.69–6.16) (Supplementary Figure S20). In biologic-exposed patients and in patients with previous biologic failure status, only upadacitinib, mirikizumab, ustekinumab, risankizumab, tofacitinib, and filgotinib 200 mg were significantly superior to placebo in the induction of endoscopic improvement with a substantial heterogeneity (I^2^ = 75%). Upadacitinib demonstrated the greatest efficacy (RR 10.52, 95% CI: 4.55–24.34) (Supplementary Figure S21).

When evaluating the maintenance of endoscopic improvement in treat-straight-through studies, a total of 13 studies were included in the meta-analysis. All active interventions were significantly superior to placebo (RR 2.03, 95% CI: 1.67–2.46) (Supplementary Figure S4). Golimumab showed the highest efficacy (RR 3.88, 95% CI: 1.66–9.03), followed by etrasimod (RR 3.23, 95% CI: 2.05–5.18), and the overall heterogeneity was substantial (I^2^ = 84%) (Supplementary Figure S5). The quality of evidence was moderate due to publication bias (Supplementary Table S3). In the evaluation of endoscopic improvement in the maintenance phase in studies with a re-randomization design, a total of 13 studies were included in the meta-analysis. All active interventions were significantly superior to placebo (RR 2.27, 95% CI: 1.97–2.61) (Supplementary Figure S6). Upadacitinib 30 mg showed the highest efficacy (RR 4.18, 95% CI: 2.79–6.27), and the overall heterogeneity was substantial (I^2^ = 90%) (Supplementary Figure S7). The quality of evidence was low due to inconsistency and publication bias (Supplementary Table S3). For the maintenance of endoscopic improvement in biologic-naive patients and in patients with no previous biologic failure status, all interventions, except for filgotinib 100 mg and vedolizumab, were significantly superior to placebo (I^2^ = 91%). Etrasimod showed the highest efficacy (RR 3.12, 95% CI: 1.79–5.43) (Supplementary Figure S22). In biologic-exposed patients and in patients with previous biologic failure status, upadacitinib, mirikizumab, ustekinumab, etrasimod, tofacitinib, and risankizumab 180 mg were significantly superior to placebo (I^2^ = 81%). Upadacitinib demonstrated the highest efficacy (RR 7.58, 95% CI: 3.42–16.8) (Supplementary Figure S23).

**Figure 3:**
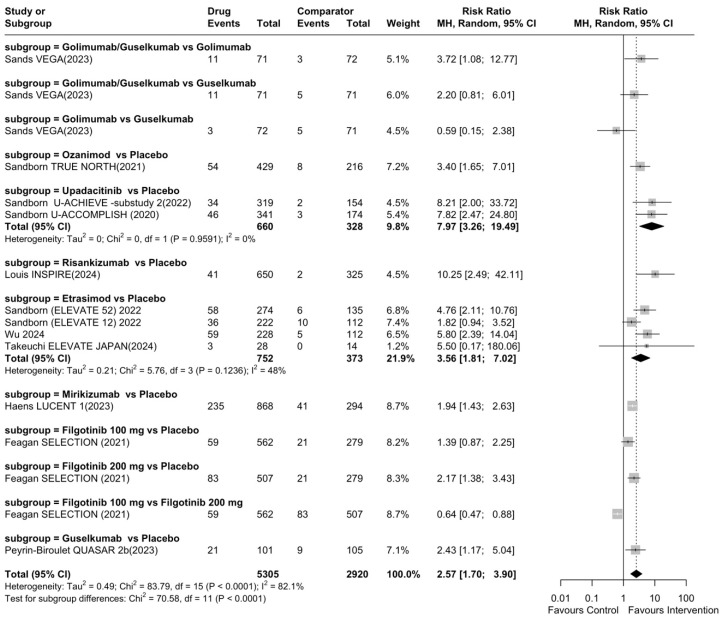
Pairwise meta-analysis for induction of mucosal healing.

In the evaluation of mucosal healing in the induction phase, a total of 12 studies were included. All the interventions were superior to placebo for the induction of mucosal healing (RR 2.95, 95% CI: 2.11–4.13), except for filgotinib 100 mg (Supplementary Figure S8). Risankizumab showed the highest efficacy (RR 10.25, 95% CI: 2.49–42.11), followed by upadacitinib (RR 7.97, 95% CI: 3.26–19.49), and the overall heterogeneity was substantial (I^2^ = 82%) (Figure 3). Results for the induction of mucosal healing are summarized in Table 2, which employs simplified layouts and includes an ‘Category’ column applying the same descriptive thresholds as above (highly efficacious: RR > 2.0; moderately efficacious: 1.0 < RR ≤ 2.0 with the 95% CI excluding 1; not efficacious: RR ≤ 1.0 or the 95% CI includes 1). The quality of evidence was low due to inconsistency and publication bias (Supplementary Table S3). When evaluating the induction of mucosal healing in biologic-naive patients and in patients with no previous biologic failure status, all interventions, except filgotinib 100 mg, were significantly superior to placebo in our direct, pairwise meta-analysis (I^2^ = 79%) (Supplementary Figure S24). In biologic-exposed patients and in patients with previous biologic failure status, only mirikizumab and ustekinumab were significantly superior to placebo in the induction of mucosal healing (I^2^ = 54%) (Supplementary Figure S25).

When evaluating the maintenance of mucosal healing, a total of seven studies with a re-randomization design and three studies [30,31,44] with a treat-straight-through design were included. All the interventions were superior to placebo (RR 1.94, 95% CI: 1.45–2.59), except for risankizumab in either 180 mg or 360 mg dose (Supplementary Figure S9). Upadacitinib 30 mg showed the highest efficacy (RR 4.01, 95% CI: 1.81–8.87), and the overall heterogeneity was substantial (I^2^ = 81%) (Supplementary Figure S11). The quality of evidence was moderate in the treat-straight-through design studies due to imprecision, whereas it was moderate in the re-randomization design trials due to inconsistency (Supplementary Table S3). For the maintenance of mucosal healing in biologic-naive patients and in patients with no previous biologic failure status, only etrasimod, mirikizumab, upadacitinib 30 mg, and subcutaneous infliximab were superior to placebo (I^2^ = 47%). Upadacitinib demonstrated the highest efficacy (RR 3.02, 95% CI: 1.18–7.71) **(**Supplementary Figure S24). In biologic-exposed patients and in patients with previous biologic failure status, only etrasimod, mirikizumab, and upadacitinib were significantly superior to placebo (I^2^ = 59%) (Supplementary Figure S25).

The results for the secondary outcomes will be analyzed alongside their respective forest plots, which are provided in the supplementary appendix from the Supplementary Figures S12–S19.

## 4. Discussion

To our knowledge, this study is the first to review all published efficacy data regarding endoscopic, histologic, and mucosal outcomes from both induction and maintenance trials of all FDA/EMA-approved drugs in patients with moderate to severe UC. This systematic review and meta-analysis synthesized data from 40 randomized controlled trials. Our findings highlight substantial variations in treatment response across different therapeutic classes, underscoring the complexity of achieving optimal disease control in UC. These findings are consistent with prior systematic reviews and meta-analyses, which have established the efficacy of these agents in UC treatment algorithms.

Several key findings have emerged from this meta-analysis that could influence therapeutic decision-making. Firstly, all approved small molecule drugs and biologics, except for mirikizumab and filgotinib 100 mg, were significantly superior compared to placebo in inducing and maintaining endoscopic improvement. Vedolizumab showed superiority over adalimumab, and the combination of golimumab/guselkumab was better than golimumab monotherapy in terms of endoscopic improvement. Upadacitinib showed the highest efficacy among advanced therapies.

Secondly, the data demonstrated that all small molecules and biologics, except filgotinib 100 mg, were significantly superior compared to placebo in inducing mucosal healing. Furthermore, all small molecules and biologics, except risankizumab in both regimens of 180 and 360 mg, showed superiority over placebo in terms of mucosal healing in the maintenance phase. The results revealed a divergence in the efficacy of risankizumab between the induction and maintenance phases. This represents a significant finding of our study and underscores the need for further prospective trials and post hoc analyses to draw safe, conclusive insights into its therapeutic role.

Thirdly, the differential efficacy observed between biologic-naive and biologic-exposed populations underscores the importance of individualized treatment selection. Biologic-naive patients generally exhibited higher response rates across all therapeutic classes, whereas biologic-experienced patients, particularly those with prior anti-TNF failure, demonstrated reduced response rates. This trend suggests that early introduction of more effective therapies, such as JAK inhibitors or IL-23 antagonists, may improve long-term disease control in select patient populations.

Our study has several strengths. We included a broad range of trials assessing not only endoscopic outcomes, but also histologic outcomes and mucosal healing—offering a multidimensional view of treatment efficacy. This comprehensive endpoint inclusion allows for a more nuanced understanding of therapeutic impact, aligned with evolving treat-to-target strategies in UC. The use of standardized definitions wherever possible, rigorous risk of bias assessments, and stratified analyses further strengthen the credibility and clinical relevance of the findings. Another strength is the stratification by trial design (treat-through vs. re-randomization), which addresses methodological heterogeneity and improves comparability within trial types. Additionally, subgroup analyses based on biologic exposure status reflect real-world clinical scenarios and enhance the external validity of our findings.

Our results are broadly consistent with the recent network meta-analysis by Estevinho et al. [57], particularly for histologic outcomes. Minor differences between the two studies likely reflect variability in how individual trials defined endpoints such as histo-endoscopic improvement and mucosal healing, which led to small differences in study inclusion for each outcome. Our pairwise meta-analysis adds complementary value by relying on direct evidence with clearly defined endoscopic and histologic endpoints.

A recent study by Shehab et al. [58] provided a comprehensive network meta-analysis of advanced therapies for ulcerative colitis, and we are pleased that our findings are largely consistent with theirs. Our analysis complements this work by incorporating the endpoint of mucosal healing, which combines endoscopic and histologic endpoints. This endpoint should be systematically incorporated into all future randomized trials, and it should be studied whether patients achieving both histologic and endoscopic remission experience more durable disease control and lower relapse rates than those achieving endoscopic improvement alone.

Our findings have immediate clinical implications. The comparative efficacy data can support evidence-based treatment selection, particularly when considering earlier introduction of highly effective therapies like upadacitinib in biologic-naive patients. Stratified efficacy outcomes emphasize the need to tailor therapy based on prior biologic exposure—a critical consideration in real-world practice where treatment sequencing remains a major challenge. The demonstrated efficacy of newer agents in both biologic-naive and biologic-experienced populations underscores their potential role in future treatment algorithms. The strong induction and maintenance data for upadacitinib across multiple endpoints suggest it may be an optimal early-line agent in high-risk patients. Similarly, the findings may guide de-escalation or step-up strategies based on therapeutic goals—whether the aim is symptom relief or endoscopic and histologic remission.

The efficacy of advanced therapies for UC is well established from clinical trial data, but real-world experience with newer IL-23p19 inhibitors (guselkumab, risankizumab, mirikizumab) is still limited. Guselkumab has only recently become available in clinical practice, having approximately one year of use in the United States and even more recent approval in Japan and Germany. As experience grows, real-world outcomes are expected to align closely with the promising efficacy observed in pivotal trials. Early real-world data for mirikizumab are emerging; a recent prospective observational study involving 20 patients with longstanding, treatment-resistant UC reported rapid improvement in symptoms and increased steroid-free remission rates. These encouraging initial results suggest that sustained improvements in endoscopic and histologic outcomes may also be observed over time [59]. Similarly, risankizumab has demonstrated sustained clinical and endoscopic responses in refractory CD, and comparable outcomes are anticipated as its use expands in UC [60]. Collectively, these initial findings underscore the considerable promise of IL-23p19 inhibition in UC management, although robust, region-specific real-world studies remain necessary to confirm long-term effectiveness, clarify safety profiles, and define optimal therapeutic positioning.

Future research should prioritize head-to-head comparative trials to further elucidate differences among therapies. Additionally, prospective, real-world studies are necessary to validate these findings outside controlled trial environments, ensuring their broader applicability and informing clinical guidelines more effectively. Standardization of endpoint definitions in future trials will further enhance the comparability and practical utility of clinical research in UC.

## 5. Limitations

Despite the robust methodology employed in our systematic review and meta-analysis, several limitations must be acknowledged. Firstly, we included some phase 2 randomized controlled trials, which typically involve smaller sample sizes. This limitation could potentially reduce the statistical power and generalizability of our findings, highlighting the necessity for larger phase 3 trials to confirm these preliminary outcomes. Additionally, for certain newer small-molecule therapies, complete datasets were not available in published manuscripts and could only be sourced from conference abstracts. Reliance on abstracts restricts the depth of data analysis and may omit important details critical to fully understanding treatment efficacy.

Secondly, our analysis integrated results from trials with varied patient populations based on prior exposure to biologic therapies. Specifically, we included trials that stratified outcomes according to biologic-naive versus biologic-experienced participants, or focused exclusively on patients with previous biologic treatment failure. Although this approach mirrors real-world scenarios, it may also introduce variability affecting the overall interpretation of comparative efficacy.

Thirdly, although we adopted standardized definitions for mucosal healing wherever possible, significant variability persisted in the reporting of endoscopic and histologic endpoints across included studies. For example, some trials assessed outcomes using “endoscopic improvement,” whereas others utilized “endoscopic remission.” Furthermore, there was variability in histological scoring systems, with some studies applying the Geboes score and others using the Robarts Histopathology Index. This methodological heterogeneity underscores the critical need for consensus among researchers to standardize endpoint definitions, facilitating better comparability across future studies and translating findings more effectively into clinical practice. Until such consensus is achieved, our results must be interpreted cautiously in the context of these differing assessment methodologies. Histologic endpoints also warrant cautious interpretation owing to the limited number of eligible studies, variability in outcome definitions, and substantial between-study heterogeneity. These constraints reduce the precision of pooled estimates and limit the generalizability of histologic findings in the present meta-analysis.

In addition, this meta-analysis did not include a pooled evaluation of adverse events. Future systematic reviews and meta-analyses focusing specifically on safety are warranted to provide a comprehensive understanding of adverse event profiles and to better guide individualized therapeutic decision-making.

Lastly, the geographic distribution of participants in the included trials represents another important limitation. The majority of enrolled patients originated from studies conducted in North America and Europe, with a smaller but still meaningful proportion from Asian populations (primarily Japan and China). However, representation from regions such as South America, Australia, and particularly Africa was very limited. This imbalance could affect the global applicability of our findings, as regional differences in healthcare systems, access to specific therapies, and patient characteristics may substantially influence therapeutic outcomes in clinical practice.

## 6. Conclusions

In conclusion, this systematic review and meta-analysis offers a comprehensive comparison of biologic and small-molecule therapies for moderate-to-severe UC, using standardized endoscopic and histologic outcomes. Most therapies demonstrated significant superiority over placebo, with upadacitinib emerging as the most efficacious agent across several endpoints. The inclusion of mucosal healing strengthens the clinical relevance of the findings and supports its use as a treatment target. Stratified analyses further highlight the importance of individualized treatment approaches based on patient history and therapeutic class. These insights can guide clinical decision-making and set the stage for more targeted and standardized research in the future.

## Figures and Tables

**Table 1:** Efficacy rank for induction of endoscopic improvement.

Rank	Drug	Comparator	Total RR (95% CI)	Category
1	Upadacitinib	Placebo	5.53 (95% CI: 3.78–8.09)	Highly efficacious
2	Risankizumab	Placebo	3.72 (95% CI: 2.65–5.22)	Highly efficacious
3	Tofacitinib	Placebo	2.45 (95% CI: 1.73–3.48)	Highly efficacious
4	Ozanimod	Placebo	2.45 (95% CI: 1.73–3.48)	Highly efficacious
5	Guselkumab	Placebo	2.44 (95% CI: 1.79–3.32)	Highly efficacious
6	Etrasimod	Placebo	2.35 (95% CI: 1.72–3.20)	Highly efficacious
7	Ustekinumab	Placebo	1.96 (95% CI: 1.41–2.72)	Moderately efficacious
8	Infliximab	Placebo	1.88 (95% CI: 1.59–2.23)	Moderately efficacious
9	Filgotinib 200 mg	Placebo	1.81 (95% CI: 1.30–2.51)	Moderately efficacious
10	Vedolizumab	Placebo	1.43 (95% CI: 1.05–1.96)	Moderately efficacious
11	Golimumab	Placebo	1.42 (95% CI: 1.15–1.75)	Moderately efficacious
12	Adalimumab	Placebo	1.26 (95% CI: 1.08–1.49)	Moderately efficacious
13	Mirikizumab	Placebo	2.55 (95% CI: 0.95–6.82)	Not efficacious (CI includes 1)
14	Filgotinib 100 mg	Placebo	1.40 (95% CI: 1.00–1.94)	Not efficacious (CI includes 1)

Cutoffs: Highly efficacious: RR > 2.0; Moderately efficacious: 1.0 < RR ≤ 2.0 and 95% CI excludes 1; Not efficacious: RR ≤ 1.0 or 95% CI includes 1. RR: risk ratio; CI: confidence interval.

**Table 2:** Efficacy rank for induction of mucosal healing.

Rank	Drug	Comparator	Total RR (95% CI)	Category
1	Risankizumab	Placebo	10.25 (95% CI: 2.49–42.11)	Highly efficacious
2	Upadacitinib	Placebo	7.97 (95% CI: 3.26–19.49)	Highly efficacious
3	Etrasimod	Placebo	3.56 (95% CI: 1.81–7.02)	Highly efficacious
4	Ozanimod	Placebo	3.40 (95% CI: 1.65–7.01)	Highly efficacious
5	Guselkumab	Placebo	2.43 (95% CI: 1.17–5.04)	Highly efficacious
6	Filgotinib 200 mg	Placebo	2.17 (95% CI: 1.38–3.43)	Highly efficacious
7	Mirikizumab	Placebo	1.94 (95% CI: 1.43–2.63)	Moderately efficacious
8	Filgotinib 100 mg	Placebo	1.39 (95% CI: 0.87–2.25)	Not efficacious

Cutoffs: Highly efficacious: RR > 2.0; Moderately efficacious: 1.0 < RR ≤ 2.0 and 95% CI excludes 1; Not efficacious: RR ≤ 1.0 or 95% CI includes 1. RR: risk ratio; CI: confidence interval.

## Data Availability

All data are provided in the article and its appendix.

## Supplementary Materials

The following supporting information can be downloaded at https://www.mdpi.com/article/10.3390/jcm14165789/s1.
