# Supplementary material for: Efficacy of Biologic Agents and Small Molecules for Endoscopic Improvement and Mucosal Healing in Patients with Moderate-to-Severe Ulcerative Colitis: Systematic Review and Meta-Analysis
Source: J Clin Med. 2025 Aug 15;14(16):5789. doi: 10.3390/jcm14165789 (PMC12386633; doi:10.3390/jcm14165789)
Supplement: Supplementary file 1 [file jcm-14-05789-s001.zip › jcm-3776452-supplementary.pdf]

Table of Contents

Supplementary Table S1: Characteristics of included studies evaluating induction of endoscopic improvement, and/or endoscopic remission, and/or histologic improvement and/or histologic remission, and/or mucosal healing in ulcerative colitis. 3

Supplementary Table S2: Characteristics of included studies evaluating maintenance of endoscopic improvement, and/or endoscopic remission, and/or histologic improvement and/or histologic remission, and/or mucosal healing in ulcerative colitis. 10

Supplementary Table S3: Biologic molecules compared to placebo or other molecules for moderate to severe ulcerative colitis 16

Supplementary Figure S1: Risk of Bias assessment 17

Supplementary Figure S2: Risk of Bias assessment 18

Supplementary Figure S3: pair-wise meta-analysis for induction of endoscopic improvement (only drugs compared to placebo) 19

Supplementary Figure S4: pair-wise meta-analysis for maintenance of endoscopic improvement in treat straight through trials (only drugs compared to placebo) 20

Supplementary Figure S5: pair-wise meta-analysis for maintenance of endoscopic improvement in treat straight through trials 21

Supplementary Figure S6: pair-wise meta-analysis for maintenance of endoscopic improvement in re-randomization only responders trials (only drugs compared to placebo) 22

Supplementary Figure S7: pair-wise meta-analysis for maintenance of endoscopic improvement in re-randomization only responders trials 23

Supplementary Figure S8: pair-wise meta-analysis for induction of mucosal healing (only drugs compared to placebo) 24

Supplementary Figure S9: pair-wise meta-analysis for maintenance of mucosal healing (only drugs compared to placebo) 25

Supplementary Figure S10: pair-wise meta-analysis for maintenance of mucosal healing in treat-straight-through trials(all drugs) 26

Supplementary Figure S11: pair-wise meta-analysis for maintenance of mucosal healing in re-randomization only responders (all drugs) 27

Supplementary Figure S12: pair-wise meta-analysis for induction of endoscopic remission (only drugs compared to placebo) 28

Supplementary Figure S13: pair-wise meta-analysis for induction of endoscopic remission (all drugs) 29

Supplementary Figure S14: pair-wise meta-analysis for maintenance of endoscopic remission (only drugs compared to placebo) 30

Supplementary Figure S15: pair-wise meta-analysis for maintenance of endoscopic remission (all drugs) 31

Supplementary Figure S16: pair-wise meta-analysis for induction of histologic improvement......32
Supplementary Figure S17: pair-wise meta-analysis for maintenance of histologic improvement......33
Supplementary Figure S18: pair-wise meta-analysis for induction of histologic remission......34
Supplementary Figure S19: pair-wise meta-analysis for maintenance of histologic remission......35
Supplementary Figure S20: pair-wise meta-analysis for induction of endoscopic improvement in biologic-naïve and no previous biologic failure status patients......36
Supplementary Figure S21: pair-wise meta-analysis for induction of endoscopic improvement in biologic-exposed and with previous biologic failure status patients......37
Supplementary Figure S22: pair-wise meta-analysis for maintenance of endoscopic improvement in biologic-naïve and no previous biologic failure status patients......38
Supplementary Figure S23: pair-wise meta-analysis for maintenance of endoscopic improvement in biologic-exposed and with previous biologic failure status patients 39
Supplementary Figure S24: pair-wise meta-analysis for induction of mucosal healing in biologic-naïve and no previous biologic failure status patients......40
Supplementary Figure S25: pair-wise meta-analysis for induction of mucosal healing in biologic-exposed and with previous biologic failure status patients......41
Supplementary Figure S24: pair-wise meta-analysis for maintenance of mucosal healing in biologic-naïve and no previous biologic failure status patients......42
Supplementary Figure S25: pair-wise meta-analysis for maintenance of mucosal healing in biologic-exposed and with previous biologic failure status patients......43
Supplementary Table S4: PRISMA checklist......44
Search Algorithm......46

Supplementary Table S1: Characteristics of included studies evaluating induction of endoscopic improvement, and/or endoscopic remission, and/or histologic improvement and/or histologic remission, and/or mucosal healing in ulcerative colitis.

AUTHOR	NCT NUMBER, NAME OF STUDY	TRIAL AND INTERVATION CHARACTERISTICS	AGE(MEAN)	MALE SEX (NUMBER %)	DISEASE DURATION (YEARS, MEAN (SD)	BIOLOGIC OR SMALL MOLECULE EXPERIENCE	ORAL GLUCOCORTICOSTEROIDS	DEFINITION OF OUTCOME
ADALIMUMAB								
Suzuki et. al	NCT00853099, ULTRA-JAPAN, INDUCTION and MAINTENANCE	Induction :ADA-90,P-96 Maintenance:ADA-177,P-96 , treat straight through design	ADA:42.6 P:41.3	ADA:61(67.8%),P:70(72.9%)	ADA:7.8±7.1, P:7.8±6.6	ADA-0, P-0	ADA-57(63.3%) P-58(60.4%)	Endoscopic Improvement MES ≤1
Reinisch et. al	NCT00385736 , ULTRA-1,INDUCTION	Induction: ADA -130, P-130	ADA:36.5 P:37	ADA:63.8% P:63.1%	ADA:6.06 P:5.35	ADA-0, P-0	ADA-48(36.9%) P-55 (41.5%)	Endoscopic Improvement MES ≤1
Sandborn et. al	NCT00408629, ULTRA -2,INDUCTION and MAINTENANCE	Induction:ADA-248 , P-246, Maintenance :ADA-248,P-246 ,treat straight through design	ADA :39.6,P:41.3	ADA:142(57.3%) ,P:152(61.8%)	ADA:8.1±7.09, P:8.5±7.37	ADA-98(39.1%) P-101(41.1%)	ADA-150(60.5%), P-140 (56.9%)	Endoscopic Improvement MES ≤1
ETRASIMOD								
Sandborn et. al	NCT03996369, ELEVATE UC12, INDUCTION	Induction: ETR-238 P-116	ETR: 40.3 ,P:40.4	ETR:135(57%),P:73(63%)	ETR:7.3, P:7.7	ETR-89(37%), P-43(37%)	ETR-177(74%),P-98(84%),	Endoscopic Improvement MES ≤1, Ensoscopic Remission MES=0, Mucosal Healing= Endoscopic Improvement and Histologic Remission by Geboes<2
Sandborn et. al	NCT03945188,ELEVATE UC 52 , INDUCTION and MAINTENANCE	Maintenance :ETR-289 , P-144, treat straight through design	ETR:41.2, P:38.9	ETR:152(53%), P:88(61%)	ETR:7.5(8.0),P:5.9(5.5)	ETR-108(37%), P- 55(38%)	ETR-224(78%), P-101(70%)	Endoscopic Improvement MES ≤1, Ensoscopic Remission MES=0, Mucosal Healing= Endoscopic Improvement and Histologic Remission by Geboes<2

Wu et.al	NCT04176588-INDUCTION and MAINTENANCE	Induction: ETR-228, P-112	NA	NA	NA	NA	NA	Endoscopic Improvement MES ≤1, Endoscopic Remission MES=0, Mucosal Healing= Endoscopic Improvement and Histologic Remission by Geboes<2
Takeuchi et.al	NCT04706793-ELEVATE JAPAN, INDUCTION and MAINTENANCE	Induction: ETR-32, P-16 Maintenance : ETR-32, P-16 treat straight through design	ETR:43.7, P:43,3	ETR: 21(55.6%), P: 8(50%)	ETR:6.2, P: 6.0	ETR-4(12.5%), P-9(56.3%)	NA	Endoscopic Improvement MES ≤1, Mucosal Healing= Endoscopic Improvement and Histologic Remission by Geboes<2
Sandborn et. al	NCT02447302,OASIS , INDUCTION	Induction:ETR-54,P-50	ETR:40.4, P:44.8,	ETR:27(54.0%), P: 32(59.3%)	ETR:6.2, P:8.6	ETR:24(48%), P:30(55.5%)	ETR:18(36%), P:16(29.6%)	Endoscopic Improvement MES ≤1, Histologic Improvement Geboes <3.1, Histologic Remission Geboes <2
FILGOTINIB								
Feagan et. al	NCT02914522,SELECTION, INDUCTIO N and MAINTENANCE	Induction A P-137, Filgotinib 100 mg -277,Filgotinib 200 mg-245 Induction B P-142, Filgotinib 100 mg -285,Filgotinib 200 mg-262 Maintenance :Placebo 3 groups-93,91,99, Filgotinib 100 mg -179, Filgotinib 200 mg -202. Re-randomisation design	Induction A:P -41, Filgotinib 100 mg-42, Filgotinib 200 mg-42 Induction B:P-44, Filgotinib 100 mg-43, Filgotinib 200 mg-43	Induction A:P -87(63.5%), Filgotinib 100 mg-157(56.7%),Filgotinib 200 mg-123(50.2%) Induction B: P-86(60.6%), Filgotinib 100 mg-186(65.3%), Filgotinib 200 mg-148(56.5%)	Induction A:P -6.4, Filgotinib 100 mg-6.7, Filgotinib 200 mg-7.2 Induction B:P-10.2, Filgotinib 100 mg-9.7, Filgotinib 200 mg-9.8	Induction A:P -0, Filgotinib 100 mg-2, Filgotinib 200 mg-0 Induction B:P-139, Filgotinib 100 mg-283, Filgotinib 200 mg-259	Induction A:P- 34, Filgotinib 100 mg-67, Filgotinib 200 mg-54 Induction B:P-51, Filgotinib 100 mg-103, Filgotinib 200 mg-94	Endoscopic Improvement MES≤1 ,Ensoscopic Remission MES=0, Histologic Remission Geboes <2, Mucosal Healing =Endoscopic Improvement and Histologic Remission
GOLIMUMAB								
Sandborn et. al	NCT00487539, PURSUIT -SC, INDUCTION	Induction: GOL-331 ,P-331	GOL:40.0, P:39.0	GOL:180(54.4%), P:175(52.9%)	GOL:6.4 ,P:6.0	GOL-0, P-0	GOL:47%, P:43%	Endoscopic Improvement MES ≤1
INFLIXIMAB								
Probert et. al	INDUCTION	Induction:IFX-23, P-20	IFX:41, P:40	NA	IFX:6.2,P:4.9	IFX=0, P=0	All patients	Endoscopic Remission -Baron score=0

Jiang et. al	INDUCTION and MAINTENANCE	Induction:IFX-41(34completed),P-41(22 completed) Maintenance:IFX-33, P-21 treat straight through design	IFX:34.3, P: 34.5	IFX:26(63.4%),P:25(60.9%)	IFX:4.4,P:4.4 ,2	IFX=0, P=0	IFX:22(53.6%),P:21(51.2%)	Endoscopic Improvement MES ≤1
CNTO312	NCT01551290,INDUCTION and MAINTENANCE	Induction :IFX-50, P-49 Maintenance:IFX-50, P-49, treat straight through design	IFX:37, P: 37	NA	IFX:3.7,P:3.7	IFX=0, P=0	IFX:30(60%), P:39(79.5%)	Endoscopic Improvement MES ≤1
Rutgeerts et.al	NCT00036439-ACT1,INDUCTION and MAINTENANCE	Induction: IFX-121,P-121 treat straight through design	IFX:42.4,P:41.4	IFX:78(64.5%) P:72(59.5%)	IFX:5.9, P:6.2,	IFX=0, P=0	IFX:70(57.8%), P:79(65.2%)	Endoscopic Improvement MES ≤1
Rutgeerts et.al	NCT00096655-ACT2,INDUCTION and MAINTENANCE	Induction :IFX-121,1-P-123 treat straight through design	IFX:40.5, P:39	IFX:76(62.8%)1, P:71(57.7%)	IFX:6.7, P:6.5	IFX=0, P=0	IFX:60(49.5%), P:60(48.7%)	Endoscopic Improvement MES ≤1
Kobayashi et. al	INDUCTION and MAINTENANCE	Induction :IFX-104,P-104 treat straight through design	IFX:40.0,P:37.8	IFX:66(63.5%),P:67(64.4%)	IFX:8.1,P:7.1	IFX=0, P=0	IFX:68(65.4%), P:69(66.3%)	Endoscopic Improvement MES ≤1
MIRIKIZUMAB								
Haens et.al	NCT03518086-LUCENT 1,INDUCTION	Induction: MIRI-868, P-294	MIRI:42.9, P:41.3	MIRI:530(61.1%), P:165(56.1%)	MIRI:7.2, P:6.9	MIRI:361(41.6%), P:118(40.1%)	MIRI:351(40.4%), P:113(38.4%)	Endoscopic Improvement MES ≤1, Mucosal Healing= Endoscopic - Histologic Improvement MES ≤1 and Geboes scoring system with neutrophil infiltration in <5% of crypts, no crypt destruction, and no erosions, ulcerations, or granulation tissue;

Sandborn et. al	NCT02589665, INDUCTION and MAINTENACE	Induction:MIRI-62, P-63 Maintenance Re-randomisation design	MIRI:43.4, P:42.6	MIRI:37(59.7%), P:36(57.1%)	MIRI:9.0,P:9.5	MIRI:40(64.5%), P:38(60%)	MIRI:25(40.3%), P:33(52.4%),	Endoscopic Improvement MES ≤1, Endoscopic Remission MES=0, Histologic Remission Geboes scoring system 0 for the neutrophils in lamina propria, neutrophils in epithelium, and erosion or ulceration parameters)
OZANIMOD								
Sandborn et. al	NCT01647516-TOUCHSTONE, INDUCTION and MAINTENANCE	Induction:OZA-67, P-65, Maintenance treat straight through design	OZA:41.8, P:41.9	OZA:48(72%), P:35(54%)	OZA:6.7, P:6.1	OZA:13(19%), P:10(15%)	OZA:27(40%), P:24(37%)	Endoscopic Improvement MES ≤1, Histologic Remission Geboes<2
Sandborn et. al	NCT02435992-TRUE NORTH, INDUCTION and MAINTENANCE	Induction:OZA-429, P-216, Maintenance Re-randomisation design	OZA:41.4, P:41.9	OZA:245(57.1%), P:143(66.2%)	OZA:6.9, P:6.8	OZA:204(47.5%), P:107(49.5%)	OZA:322(75.1%), P:62(75%)	Endoscopic Improvement MES ≤1, Mucosal Healing= Endoscopic Improvement and Histologic Remission by Geboes<2
TOFACITINIB								
Sandborn et. al	NCT00787202, INDUCTION	Induction: TOF-33, P-48	TOF:43.2, P:42.5	TOF:21(64%), P:23(48%)	TOF:10.9, P:8.8	TOF:10(30%), P:15(31%)	TOF:19(58%), P:13(27%)	Endoscopic Remission MES=0
Sandborn et. al	NCT01465763-OCTAVE 1, INDUCTION	Induction:TOF-476, P-122	TOF:41.3, P:41.8	TOF:277(58.2%), P:77(63.1%)	TOF:6.5,P:6.0	TOF:254(53.4%), P:65(53.3%)	TOF:214(45.0%),P:58(47.5%)	Endoscopic Improvement MES≤1 Endoscopic Remission MES=0
Sandborn et. al	NCT01458951-OCTAVE 2, INDUCTION	Induction:TOF-429, P-112	TOF:41.1,P:40.4	TOF:259(60.4%),P:55(49.1%)	TOF:6.0,P:6.2	TOF:234(54.5%), P:65(58.0%)	TOF:198(46.2%),P:55(49.1%)	Endoscopic Improvement MES≤1 Endoscopic Remission MES=0
UPADACITINIB								

Sandborn et. al	NCT02819635, U-ACHIEVE-Induction-SUBSTUDY 1	Induction:UPA-56, P-46	UPA:37, P:40	UPA:37(66.1%),P:29(63%)	UPA:6.46,P:5.19	UPA:43(76.8%),P:35(76.1%)	UPA:29(51.8%),P:30(65.2%)	Endoscopic Improvement MES≤1 Endoscopic Remission MES=0, Histologic Improvement Any decrease in Geboes scoring system
Danese et.al	NCT02819635, U-ACHIEVE-Induction-SUBSTUDY 2	Induction:UPA-319, P-154	UPA:43.0,P:44.5	UPA:198(62%),P:97(63%)	UPA:6.6, P:6.0	UPA:168(53%), P:78(51%)	UPA:124(39%),P:61(40%)	Endoscopic Improvement MES ≤1, Endoscopic Remission MES=0, Histologic Improvement Any decrease in Geboes scoring system, Mucosal Healing= Endoscopic Remission and Histologic Remission by Geboes<2
Danese et.al	NCT03653026-U-ACCOMPLISH, INDUCTION	Induction:UPA-341, P-174	UPA:40.0, P:42.0	UPA:214(63%), P:107(61%)	UPA:5.6, P:4.9	UPA:172(50%),P:89(51%)	UPA:120(35%),P:72(41%)	Endoscopic Improvement MES ≤1, Ensoscopic Remission MES=0, Histologic Improvement Any decrease in Geboes scoring system, Mucosal Healing= Endoscopic Remission and Histologic Remission by Geboes<2
USTEKINUMAB								
Sands et .al	NCT02407236-UNIFI, INDUCTION and MAINTENANCE	Induction:UST-322, P-319 Maintenance :UST-176,P-175 Re-randomisation design	UST:41.7, P:41.2	UST:195(60.6%), P:197(61.8%)	UST:8.2, P:8.0	UST:166(51.6%),P:161(50.5%)	UST:168(52.2%),P:157(49.2%)	Endoscopic Improvement MES ≤1, Histologic improvement Geboes scoring system 0-<5% neutrophils in epithelium, no crypt destruction, and no erosions or ulcerations or granulations

VEDOLIZUMAB								
Feagan et. al	NCT00783718-GEMINI I, INDUCTION and MAINTENANCE	Induction :VEDO-225,P-149, Maintenance:VEDO-122,P-126 Re-randomisation design	VEDO:40.1,P:41	VEDO:132(58.7%),P:92(61.7%)	VEDO:6.1,P:7.1	VEDO:95(42.2%),P:73(49%)	VEDO:79(35.1%),P:58(38.9%)	Endoscopic Improvement MES ≤1
Motoya et.al	NCT02039505, INDUCTION and MAINTENANCE	Induction :VEDO-164, P-82, Maintenance:VEDO-41, P-42 Re-randomisation design	VEDO:42.3, P:44.0	VEDO:99(60.4%), P:55(67.1%)	VEDO:7.2, P:8.6	VEDO:85(51.8%), P:41(50.0%)	VEDO:31(18.9%), P:11(13.4%)	Endoscopic Improvement MES ≤1
RISANKIZUMAB								
Louis et.al	NCT03398148-INSPIRE, INDUCTION	Induction: RIS-650, P-325	RIS:41.8, P;42.8	RIS:385(59.2%), P:201(61.8%)	RIS:7.7, P:8.1	NA	RIS:236(36.3%), P:112(34.5%)	Endoscopic Improvement MES ≤1, Endoscopic Remission MES=0, Mucosal Healing= Endoscopic Remission and Histologic Remission by Geboes<2
GOLIMUMAB AND GUSELKUMAB								
Feagan et .al	NCT03662542-VEGA, INDUCTION and MAINTENANCE	Induction:GOL-72, GUS-71, GOL/GUS-71, Maintenance treat straight through strategy	GOL:38.1, GUS:39.1, GOL/GUS:37.8	GOL:42(58%), GUS:40(56%), GOL/GUS:34(48%)	GOL:4.7,GUS:5.4, GOL/GUS:4.6	NA	NA	Endoscopic Improvement MES ≤1, Ensoscopic Remission MES=0, Histologic Remission Geboes<2, Mucosal Healing= Endoscopic Remission and Histologic Remission by Geboes<2
GUSELKUMAB								

Rubin et.al	NCT04033445-Quasar phase 3, INDUCTION and MAINTENANCE	Induction :Guselkumab 200mg-190, Guselkumab 100mg-188, P-190, Maintenance Re-randomisation design	NA	NA	NA	NA	NA	Endoscopic Improvement MES ≤1, Endoscopic Remission MES=0, Histologic Remission Geboes≤2, Histologic Improvement Geboes ≤3.1, Mucosal Healing= Endoscopic Remission and Histologic Remission by Geboes ≤2
Peyrin-Biroulet et.al	NCT04033435-QUASAR 2b, INDUCTION	Induction: GUS-101, P-105	GUS:43.3, P:41.2	GUS:60(59.4%),P:66(62.9%)	GUS:7.0,P:7.7	GUS:53(52.4%), P:54(51.4%)	GUS:41(40.6%),P:40(38.1%)	Endoscopic Improvement MES ≤1, Endoscopic Remission MES=0, Mucosal healing = Endoscopic improvement and Histologic improvement Geboes score ≤3.1)

ADA=Adalimumab, Baron score: 0- normal, 1- abnormal mucosal but not hemorrhagic, 2- friability, easy to bleeding , 3 -spontaneous bleeding, ETR=Etrasimod GOL=Golimumab, GUS=Guselkumab, IFX=Infliximab, MES=Mayo Endoscopic Score, MIRI=Mirikizumab, P=Placebo, OZA=Ozanimod, , RIS= Risankizumab TOF=Tofacitinib, UPA=Upadacitinib, , UST= Ustekinumab, VEDO=Vedolizumab

Supplementary Table S2: Characteristics of included studies evaluating maintenance of endoscopic improvement, and/or endoscopic remission, and/or histologic improvement and/or histologic remission, and/or mucosal healing in ulcerative colitis.

AUTHOR	NCT NUMBER, NAME OF STUDY	TRIAL AND INTERVATION CHARACTERISTICS	AGE(MEAN)	MALE SEX (NUMBER %)	DISEASE DURATION (YEARS, MEAN (SD)	BIOLOGIC OR SMALL MOLECULE EXPERIENCE	ORAL GLUCOCORTICOSTEROIDS	DEFINITION OF OUTCOME
ADALIMUMAB								
Suzuki et. al	NCT00853099, ULTRA-JAPAN, INDUCTION and MAINTENANCE	Induction :ADA-90,P-96 Maintenance:ADA-177,P-96 , treat straight through design	ADA:42.6 P:41.3	ADA:61(67.8%),P:70(72.9%)	ADA:7.8±7.1, P:7.8±6.6	ADA-0, P-0	ADA-57(63.3%) P-58(60.4%)	Endoscopic Improvement MES ≤1
Sandborn et. al	NCT00408629, ULTRA -2,INDUCTION and MAINTENANCE	Induction:ADA-248 , P-246, Maintenance :ADA-248,P-246 ,treat straight through design	ADA :39.6,P:41.3	ADA:142(57.3%) ,P:152(61.8%)	ADA:8.1±7.09, P:8.5±7.37	ADA-98(39.1%) P-101(41.1%)	ADA-150(60.5%), P-140 (56.9%)	Endoscopic Improvement MES ≤1
ETRASIMOD								
Sandborn et. al	NCT03945188,ELEVATE UC 52 , INDUCTION and MAINTENANCE	Maintenance :ETR-289 , P-144, treat straight through design	ETR:41.2, P:38.9	ETR:152(53%), P:88(61%)	ETR:7.5(8.0),P:5.9(5.5)	ETR-108(37%), P- 55(38%)	ETR-224(78%), P-101(70%)	Endoscopic Improvement MES ≤1, Ensoscopic Remission MES=0, Mucosal Healing= Endoscopic Improvement and Histologic Remission by Geboes<2
Takeuchi et.al	NCT04706793-ELEVATE JAPAN, INDUCTION and MAINTENANCE	Induction: ETR-32, P-16 Maintenance : ETR-32, P-16 treat straight through design	ETR:43.7, P:43,3	ETR: 21(55.6%), P: 8(50%)	ETR:6.2, P: 6.0	ETR-4(12.5%), P-9(56.3%)	NA	Endoscopic Improvement MES ≤1, Ensoscopic Remission MES=0, Mucosal Healing= Endoscopic Improvement and Histologic Remission by Geboes<2
FILGOTINIB								

Feagan et. al	NCT02914522,SELECTION, INDUCTION and MAINTENANCE	Induction A P-137, Filgotinib 100 mg -277,Filgotinib 200 mg-245 Induction B P-142, Filgotinib 100 mg -285,Filgotinib 200 mg-262 Maintenance :Placebo 3 groups-93,91,99, Filgotinib 100 mg -179, Filgotinib 200 mg -202. Re-randomisation design	Induction A:P -41, Filgotinib 100 mg-42, Filgotinib 200 mg-42 Induction B:P-44, Filgotinib 100 mg-43, Filgotinib 200 mg-43	Induction A:P -87(63.5%), Filgotinib 100 mg-157(56.7%),Filgotinib 200 mg-123(50.2%) Induction B: P-86(60.6%), Filgotinib 100 mg-186(65.3%), Filgotinib 200 mg-148(56.5%)	Induction A:P -6.4, Filgotinib 100 mg-6.7, Filgotinib 200 mg-7.2 Induction B:P-10.2, Filgotinib 100 mg-9.7, Filgotinib 200 mg-9.8	Induction A:P -0, Filgotinib 100 mg-2, Filgotinib 200 mg-0 Induction B:P-139, Filgotinib 100 mg-283, Filgotinib 200 mg-259	Induction A:P- 34, Filgotinib 100 mg-67, Filgotinib 200 mg-54 Induction B:P-51, Filgotinib 100 mg-103, Filgotinib 200 mg-94	Endoscopic Improvement MES≤1 ,Ensoscopic Remission MES=0, Histologic Remission Geboes <2, Mucosal Healing =Endoscopic Improvement and Histologic Remission
GOLIMUMAB								
Hibi et.al	NCT01863771, PURSUIT-J, INDUCTION-OPEN LABEL and MAINTENANCE -DOUBLE BLINDED	Maintenance :GOL -32,P-31	GOL:39.3,P:42.9	GOL:19(59%), P:19(61%)	GOL:5.35, P:5,74	GOL-0, P-0	GOL:9(28%), 2-9(29%)	Endoscopic Improvement MES ≤1
Sandborn et. al	NCT00488631, PURSUIT-M,MAINTENANCE only randomised	Maintenance :GOL-154, P-156, Re-randomisation design	GOL:39,1, P:40.2	GOL:89(57.8%), P:75(48.1%)	GOL:7.2, P:-6.9	GOL-0, P-0	GOL-79(51.3%), P-83(53.2%)	Endoscopic Improvement MES ≤1
INFLIXIMAB								
Jiang et. al	INDUCTION and MAINTENANCE	Induction:IFX-41(34completed),P-41(22 completed) Maintenance:IFX-33, P-21 treat straight through design	IFX:34.3, P: 34.5	IFX:26(63.4%),P:25(60.9%)	IFX:4.4,P:4.4 ,2	IFX=0, P=0	IFX:22(53.6%),P:21(51.2%)	Endoscopic Improvement MES ≤1
CNTO312	NCT01551290,INDUCTION and MAINTENANCE	Induction :IFX-50, P-49 Maintenance:IFX-50, P-49, treat straight through design	IFX:37, P: 37	NA	IFX:3.7,P:3.7	IFX=0, P=0	IFX:30(60%), P:39(79.5%)	Endoscopic Improvement MES ≤1
Rutgeerts et.al	NCT00036439-ACT1,INDUCTION and MAINTENANCE	Induction: IFX-121,P-121 treat straight through design	IFX:42.4,P:41.4	IFX:78(64.5%) P:72(59.5%)	IFX:5.9, P:6.2,	IFX=0, P=0	IFX:70(57.8%), P:79(65.2%)	Endoscopic Improvement MES ≤1
Rutgeerts et.al	NCT00096655-ACT2,INDUCTION and MAINTENANCE	Induction :IFX-121,1-P-123 treat straight through design	IFX:40.5, P:39	IFX:76(62.8%)1, P:71(57.7%)	IFX:6.7, P:6.5	IFX=0, P=0	IFX:60(49.5%), P:60(48.7%)	Endoscopic Improvement MES ≤1
Kobayashi et. al	INDUCTION and MAINTENANCE	Induction :IFX-104,P-104 treat straight through design	IFX:40.0,P:37.8	IFX:66(63.5%),P:67(64.4%)	IFX:8.1,P:7.1	IFX=0, P=0	IFX:68(65.4%), P:69(66.3%)	Endoscopic Improvement MES ≤1

Sands et .all	NCT04205643-LIBERTY-UC, MAINTENANCE	Maintenance :IFX-294, P-144, Re-randomisation design	IFX:37,P:39	IFX:163(55.4%), P:83(57.6%)	IFX:6.09, P:6.8	IFX:29(9.9%), P:13(9.0%)	IFX:120(40.8%), P:61(42.4%)	Endoscopic - Histologic Improvement MES ≤1 and RHI≤3
MIRIKIZUMAB								
Haens et.al	NCT03524092-LUCENT 2, MAINTENANCE	Maintenance:MIRI-365, P-179 Re-randomisation design	MIRI:43.4, P:41.2	MIRI:214(58.6%), P:104(58.1%)	MIRI:6.9, P:6.7	MIRI:128(35.1%), P:64(35.8%)	MIRI:135(37.0%), P:68(38.0%)	Endoscopic Improvement MES ≤1, Endoscopic - Histologic Improvement MES ≤1 and Geboes scoring system with neutrophil infiltration in <5% of crypts, no crypt destruction, and no erosions, ulcerations, or granulation tissue;
Sandborn et. al	NCT02589665, INDUCTION and MAINTENACE	Induction:MIRI-62, P-63 Maintenance Re-randomisation design	MIRI:43.4, P:42.6	MIRI:37(59.7%), P:36(57.1%)	MIRI:9.0,P:9.5	MIRI:40(64.5%), P:38(60%)	MIRI:25(40.3%), P:33(52.4%),	Endoscopic Improvement MES ≤1, Endoscopic Remission MES=0, Histologic Remission Geboes scoring system 0 for the neutrophils in lamina propria, neutrophils in epithelium, and erosion or ulceration parameters)
OZANIMOD								
Sandborn et. al	NCT01647516-TOUCHSTONE, INDUCTION and MAINTENANCE	Induction:OZA-67, P-65, Maintenance treat straight through design	OZA:41.8, P:41.9	OZA:48(72%), P:35(54%)	OZA:6.7, P:6.1	OZA:13(19%), P:10(15%)	OZA:27(40%), P:24(37%)	Endoscopic Improvement MES ≤1, Histologic Remission Gebos<2
Sandborn et. al	NCT02435992-TRUE NORTH, INDUCTION and MAINTENANCE	Induction:OZA-429, P-216, Maintenance Re-randomisation design	OZA:41.4, P:41.9	OZA:245(57.1%), P:143(66.2%)	OZA:6.9, P:6.8	OZA:204(47.5%), P:107(49.5%)	OZA:322(75.1%), P:62(75%)	Endoscopic Improvement MES ≤1, Mucosal Healing= Endoscopic Improvement and Histologic Remission by Geboes<2

TOFACITINIB								
Sandborn et. al	NCT01458574-OCTAVE Sustain, MAINTENANCE	Maintenance:Tofacitinib 5mg-198, Tofacitinib 10mg-197, P-198, Re-randomisation design	Tofacitinib 5mg :41.9, Tofacitinib 10 mg:42.9, P:43.4	Tofacitinib 5mg:103(52%), Tofacitinib 10 mg :110(55.8%), P:116(58.6%)	Tofacitinib 5mg:6.5, Tofacitinib 10mg:6.8,P:7.2	Tofacitinib 5mg:90(45.5%), Tofacitinib 10 mg:101(51.3%),P:92(46.5%)	Tofacitinib 5mg :101(51.0%), Tofacitinib 10mg:87(44.2%),P:100(50.5%)	Endoscopic Improvement MES≤1 Endoscopic Remission MES=0
UPADACITINIB								
Danese et.al	NCT02819635, U-ACHIEVE-Maintenance -SUBSTUDY 3	Maintenance:Upadacitinib 15mg-148,Upadacitinib 30mg-154, P-149 Re-randomisation design	Upadacitinib 15mg:40, Upadacitinib 30mg:41,P:40	Upadacitinib 15mg:95(64%), Upadacitinib 30mg:86(56%), P:85(57%)	Upadacitinib 15mg:6.4, Upadacitinib 30mg:6.0, P:6.2	Upadacitinib 15mg:71(48%), Upadacitinib 30mg:73(47%), P:81(54%)	Upadacitinib 15mg55(37%), Upadacitinib 30mg:57(37%), P:60(40%)	Endoscopic Improvement MES ≤1, Ensoscopic Remission MES=0, Mucosal Healing= Endoscopic Remission and Histologic Remission by Geboes<2
USTEKINUMAB								
Sands et .al	NCT02407236-UNIFI, INDUCTION and MAINTENANCE	Induction:UST-322, P-319 Maintenance :UST-176,P-175 Re-randomisation design	UST:41.7, P:41.2	UST:195(60.6%), P:197(61.8%)	UST:8.2, P:8.0	UST:166(51.6%),P:161(50.5 %)	UST:168(52.2%),P:157(49.2%)	Endoscopic Improvement MES ≤1, Histologic improvement Geboes scoring system 0-<5% neutrophils in epithelium, no crypt destruction, and no erosions or ulcerations or granulations
VEDOLIZUMAB								
Sands et .al	NCT02497469-VARSITY, INDUCTION and MAINTENANCE	ADA-386, VEDO-385 treat straight through design	ADA:40.5,VEDO:40.8	ADA:216(56%), VEDO:234(60.8%)	ADA:6.4, VEDO:7.3	ADA:81(21%), VEDO:80(20.8%)	ADA:140(36.3%), VEDO:139(36.1%)	Endoscopic Improvement MES ≤1, Histologic Remission Geboes Scoring System <2, Histologic Improvement Geboes Scoring System <3.2

Feagan et. al	NCT00783718-GEMINI I, INDUCTION and MAINTENANCE	Induction :VEDO-225,P-149, Maintenance:VEDO-122,P-126 Re-randomisation design	VEDO:40.1,P:41	VEDO:132(58.7%),P:92(61.7%)	VEDO:6.1,P:7.1	VEDO:95(42.2%),P:73(49%)	VEDO:79(35.1%),P:58(38.9%)	Endoscopic Improvement MES ≤1
Motoya et.al	NCT02039505, INDUCTION and MAINTENANCE	Induction :VEDO-164, P-82, Maintenance:VEDO-41, P-42 Re-randomisation design	VEDO:42.3, P:44.0	VEDO:99(60.4%), P:55(67.1%)	VEDO:7.2, P:8.6	VEDO:85(51.8%), P:41(50.0%)	VEDO:31(18.9%), P:11(13.4%)	Endoscopic Improvement MES ≤1
Sandborn et. al	NCT02611830-VISIBLE I, INDUCTION and MAINTENANCE	Maintenance:Vedolizumab SC-106, Vedolizumab IV-54,P-56 Re-randomisation design	Vedolizumab SC:38.1, Vedolizumab IV:41.6, P:39.4	Vedolizumab SC:65(61.3%), Vedolizumab IV:31(57.4%), P:34(60.7%)	Vedolizumab SC:8.0, Vedolizumab IV:8.2, P:7.4	Vedolizumab SC :40(37.7%), Vedolizumab IV:24(44.4%), P:20(35.7%)	Vedolizumab SC:28(26.4%), Vedolizumab IV:21(38.9%), P:22(39.3%)	Endoscopic Improvement MES ≤1
RISANKIZUMAB								
Louis et.al	NCT03398135-COMMAND, MAINTENANCE	Maintenance :Risankizumab 180mg-179, Risankizumab 360mg-186, P-183 Re-randomisation design	Risankizumab 180 mg:40.9, Risankizumab 360mg:42.5, P:39.2	Risankizumab 180mg:105(58.7%), Risankizumab 360mg :107(57.5%), P;101(55.2%)	Risankizumab180mg:8.5, Risankizumab 360mg:9.3, P:8.2	NA	Risankizumab180mg:74(41.3%), Risankizumab360mg :59(31.7%), P:68(37.2%)	Endoscopic Improvement MES ≤1, Ensoscopic Remission MES=0, Mucosal Healing= Endoscopic Remission and Histologic Remission by Geboes<2
GOLIMUMAB AND GUSELKUMAB								
Feagan et .al	NCT03662542-VEGA, INDUCTION and MAINTENANCE	Induction:GOL-72, GUS-71, GOL/GUS-71, Maintenance treat straight through strategy	GOL:38.1, GUS:39.1, GOL/GUS:37.8	GOL:42(58%), GUS:40(56%), GOL/GUS:34(48%)	GOL:4.7,GUS:5.4, GOL/GUS:4.6	NA	NA	Endoscopic Improvement MES ≤1, Ensoscopic Remission MES=0, Histologic Remission Geboes<2, Mucosal Healing= Endoscopic Remission and Histologic Remission by Geboes<2
GUSELKUMAB								

Rubin et.al	NCT04033445-Quasar phase 3, INDUCTION and MAINTENANCE	Induction :Guselkumab 200mg-190, Guselkumab 100mg-188, P-190, Maintenance Re-randomisation design	NA	NA	NA	NA	NA	Endoscopic Improvement MES ≤1, Ensoscopic Remission MES=0, Histologic Remission Geboes≤2, Mucosal Healing= Endoscopic Remission and HistologicRemission by Geboes ≤2

ADA=Adalimumab, Baron score: 0- normal, 1- abnormal mucosal but not hemorrhagic, 2- friability, easy to bleeding , 3 -spontaneous bleeding, ETR=Etrasimod GOL=Golimumab, GUS=Guselkumab, IFX=Infliximab, MES=Mayo Endoscopic Score, MIRI=Mirikizumab, P=Placebo, OZA=Ozanimod, , RIS= Risankizumab TOF=Tofacitinib, UPA=Upadacitinib, , UST= Ustekinumab

Supplementary Table S3: Biologic molecules compared to placebo or other molecules for moderate to severe ulcerative colitis

Patient or population: moderate to severe ulcerative colitis
Setting: outpatient
Intervention: biologic molecules
Comparison: placebo or other molecules

Outcomes	№ of participants (studies) Follow-up	Certainty of the evidence (GRADE)	Relative effect (95% CI)	Anticipated absolute effects	
				Risk with placebo or other molecules	Risk difference with biologic molecules
INDUCTION ENDOSCOPIC IMPROVEMT	14369 (30 RCTs)	⊕⊕◯◯ Low[a,b]	**RR 1.90** (1.67 to 2.13)	191 per 1,000	**172 more per 1,000** (128 more to 216 more)
INDUCTION MUCOSAL HEALING	8225 (12 RCTs)	⊕⊕◯◯ Low[c,d]	**RR 3.12** (2.22 to 4.23)	77 per 1,000	**163 more per 1,000** (94 more to 248 more)
MAINTENACE ENDOSCOPIC IMPROVEMENT TST	3473 (13 RCTs)	⊕⊕⊕◯ Moderate[e]	**RR 1.68** (1.28 to 2.10)	252 per 1,000	**171 more per 1,000** (71 more to 277 more)
MAINTENANCE ENDOSCOPIC IMPROVENT RR	7738 (13 RCTs)	⊕⊕◯◯ Low[f,g]	**RR 1.77** (1.53 to 2.01)	287 per 1,000	**221 more per 1,000** (152 more to 289 more)
MAINTENANCE MUCOSAL HEALING TST	867 (3 RCTs)	⊕⊕⊕◯ Moderate[h]	**RR 2.09** (1.02 to 3.81)	96 per 1,000	**105 more per 1,000** (2 more to 271 more)
MAINTENANCE MUCOSAL HEALING RR	5511 (7 RCTs)	⊕⊕⊕◯ Moderate[i]	**RR 1.92** (1.35 to 2.60)	159 per 1,000	**147 more per 1,000** (56 more to 255 more)

***The risk in the intervention group** (and its 95% confidence interval) is based on the assumed risk in the comparison group and the **relative effect** of the intervention (and its 95% CI).

CI: confidence interval; **RR:** risk ratio ; **TST**: Treat straight through ; **RR**: Re-Randomization

GRADE Working Group grades of evidence
High certainty: we are very confident that the true effect lies close to that of the estimate of the effect.
Moderate certainty: we are moderately confident in the effect estimate: the true effect is likely to be close to the estimate of the effect, but there is a possibility that it is substantially different.
Low certainty: our confidence in the effect estimate is limited: the true effect may be substantially different from the estimate of the effect.
Very low certainty: we have very little confidence in the effect estimate: the true effect is likely to be substantially different from the estimate of effect.

Explanations

a. Rated down for one level for inconsistency (I^2=71%)
b. Funnel plot asymmetry, p-value <0.05 in Eager's test
c. Rated down for one level for inconsistency (I^2=82%)
d. Funnel plot asymmetry , p-value <0.05 in Eager's test
e. Funnel plot asymmetry, p-value <0.05 in Eager's test
f. Rated down for one level for inconsistency(I^2=61%).
g. Funnel plot asymmetry , p-value <0.05 in Eager's test
h. Lack of sufficient statistical power. Downgraded by 1 level for imprecision
i. Rated down due to Inconsistency (I^2=81%)

Supplementary Figure S1: Risk of Bias assessment

Risk of bias domains

Study	D1	D2	D3	D4	D5	Overall
ULTRA-JAPAN-2014	+	+	+	+	+	+
ULTRA 1-2012	+	+	+	+	+	+
ULTRA -2-2011	+	+	+	+	+	+
ELEVATE UC 52-2023	+	+	+	+	+	+
ELEVATE UC 12-2023	+	+	+	+	+	+
OASIS-2020	+	+	+	+	+	+
ELEVATE JAPAN-2024	+	+	+	+	+	+
Wu-2024	+	+	+	+	+	+
PURSUIT-M-2014	+	+	+	+	+	+
PURSUIT-J-2017	+	+	+	+	+	+
PURSUIT -SC phase 2 A-2014	+	+	+	+	+	+
PURSUIT -SC phase 3-2014	+	+	+	+	+	+
PURSUIT -SC phase 2 B-2014	+	+	+	+	+	+
Probert et. al-2003	+	+	+	-	+	-
Jiang et. al-2015	+	+	-	+	+	-
CNTO312-2012	+	+	+	+	+	+
ACT1-2005	+	+	+	+	+	+
ACT2-2005	+	+	+	+	+	+
LIBERTY-UC-2023	+	+	-	+	+	-
Kobayashi-2016	+	+	+	+	+	+
Sandborn-2020	+	+	+	+	+	+
LUCENT 2-2024	+	+	+	+	+	+
LUCENT 1-2024	+	+	+	+	+	+
TOUCHSTONE-2016	+	+	-	+	+	-
TRUE NORTH-2021	+	+	+	+	+	+
Sandborn -2012	+	+	+	+	+	+
OCTAVE 1-2017	+	+	+	+	+	+
OCTAVE Sustain-2017	+	+	+	+	+	+
OCTAVE 2-2017	+	+	+	+	+	+
U-ACHIEVE-induction-SUBSTUDY 1-2020	+	+	+	+	+	+
U-ACHIEVE-induction-SUBSTUDY 2-2022	+	+	+	+	+	+
U-ACCOMPLISH-2022	+	+	+	+	+	+
U-ACHIEVE-Maintenance -SUBSTUDY 3-2022	+	+	+	+	+	+
UNIFI-2019	+	+	+	+	+	+
GEMINI I-2013	+	+	+	+	+	+
Motoya-2019	+	+	+	+	+	+
VARSITY-2019	+	+	+	+	+	+
VISIBLE I-2020	+	+	+	+	+	+
INSPIRE-2024	+	+	+	+	+	+
Louis- Phase 2-2022	+	+	+	+	+	+
COMMAND-2024	+	+	+	+	+	+
VEGA-2023	+	+	+	+	+	+
QUASAR 2b-2023	+	+	+	+	+	+
QUASAR 3-2024	+	+	+	+	+	+
SELECTION-2021	+	+	+	+	+	+

Domains:
D1: Bias arising from the randomization process.
D2: Bias due to deviations from intended intervention.
D3: Bias due to missing outcome data.
D4: Bias in measurement of the outcome.
D5: Bias in selection of the reported result.

Judgement
- Some concerns
+ Low

Supplementary Figure S2: Risk of Bias assessment

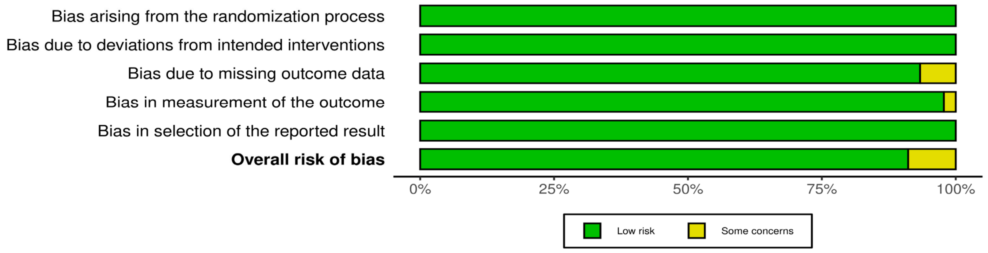

Supplementary Figure S3: pair-wise meta-analysis for induction of endoscopic improvement (only drugs compared to placebo)

Study or Subgroup	Drug Events	Total	Comparator Events	Total	Weight	Risk Ratio MH, Random, 95% CI
**subgroup = Adalimumab vs Placebo**						
Suzuki et. al,NCT00853099, ULTRA-JAPAN	40	90	29	96	3.5%	1.47 [1.00; 2.16]
Reinisch et. al,NCT00385736 , ULTRA 1	61	130	54	130	4.0%	1.13 [0.86; 1.49]
Sandborn et. al,NCT00408629, ULTRA -2	102	248	78	246	4.2%	1.30 [1.02; 1.64]
**Total (95% CI)**		**468**		**472**	**11.7%**	**1.26 [1.08; 1.49]**
Heterogeneity: $Tau^2 = 0$; $Chi^2 = 1.3$, df = 2 (P = 0.52); $I^2 = 0\%$						
**subgroup = Etrasimod vs Placebo**						
Sandborn et. al,NCT03945188, ELEVATE UC 52	96	274	19	135	3.2%	2.49 [1.59; 3.89]
Sandborn et. al,NCT 03996369, ELEVATE UC 12	68	222	21	112	3.2%	1.63 [1.06; 2.52]
Sandborn et. al,NCT02447302,OASIS	21	50	10	54	2.3%	2.27 [1.19; 4.33]
Wu et.al ,NCT04176588	85	228	11	112	2.5%	3.80 [2.11; 6.82]
Takeuchi et.al,NCT04706793-ELEVATE JAPAN	5	28	1	14	0.4%	2.50 [0.32; 19.40]
**Total (95% CI)**		**802**		**427**	**11.7%**	**2.35 [1.72; 3.20]**
Heterogeneity: $Tau^2 = 0.03$; $Chi^2 = 5.44$, df = 4 (P = 0.24); $I^2 = 27\%$						
**subgroup = Golimumab vs Placebo**						
Sandborn et. al,NCT00487539, PURSUIT -SC phase 2 B	15	30	12	28	2.7%	1.17 [0.67; 2.04]
Sandborn et. al,NCT00487539, PURSUIT -SC phase 2 A	14	41	10	41	2.2%	1.40 [0.70; 2.78]
Sandborn et. al,NCT00487539, PURSUIT -SC phase 3	107	253	72	251	4.2%	1.47 [1.16; 1.88]
**Total (95% CI)**		**324**		**320**	**9.0%**	**1.42 [1.15; 1.75]**
Heterogeneity: $Tau^2 = 0$; $Chi^2 = 0.57$, df = 2 (P = 0.75); $I^2 = 0\%$						
**subgroup = Infliximab vs Placebo**						
Jiang et. al	24	41	10	41	2.5%	2.40 [1.32; 4.36]
CNTO312, NCT01551290	17	50	8	49	2.0%	2.08 [0.99; 4.37]
Rutgeerts et.al,NCT00036439-ACT1	75	121	41	121	4.0%	1.83 [1.38; 2.43]
Rutgeerts et.al,NCT00096655-ACT2	73	121	38	123	3.9%	1.95 [1.44; 2.64]
Kobayashi et.al	48	104	29	104	3.5%	1.66 [1.14; 2.40]
**Total (95% CI)**		**437**		**438**	**15.8%**	**1.88 [1.59; 2.23]**
Heterogeneity: $Tau^2 = 0$; $Chi^2 = 1.26$, df = 4 (P = 0.87); $I^2 = 0\%$						
**subgroup = Mirikizumab vs Placebo**						
Haens et.al,NCT03518086-LUCENT 1	315	868	62	294	4.2%	1.72 [1.36; 2.18]
Sandborn et. al,NCT02589665	19	62	4	63	1.3%	4.83 [1.74; 13.38]
**Total (95% CI)**		**930**		**357**	**5.5%**	**2.55 [0.95; 6.82]**
Heterogeneity: $Tau^2 = 0.39$; $Chi^2 = 3.75$, df = 1 (P = 0.05); $I^2 = 73\%$						
**subgroup = Ozanimod vs Placebo**						
Sandborn et. al,NCT01647516-TOUCHSTONE	23	67	8	65	2.0%	2.79 [1.35; 5.78]
Sandborn et. al,NCT02435992-TRUE NORTH	117	429	25	216	3.4%	2.36 [1.58; 3.51]
**Total (95% CI)**		**496**		**281**	**5.4%**	**2.45 [1.73; 3.48]**
Heterogeneity: $Tau^2 = 0$; $Chi^2 = 0.16$, df = 1 (P = 0.69); $I^2 = 0\%$						
**subgroup = Tofacitinib vs Placebo**						
Sandborn et. al,NCT01465763-OCTAVE 1	149	476	19	122	3.2%	2.01 [1.30; 3.10]
Sandborn et. al,NCT01458951-OCTAVE 2	122	429	13	112	2.8%	2.45 [1.44; 4.17]
**Total (95% CI)**		**905**		**234**	**6.0%**	**2.18 [1.55; 3.05]**
Heterogeneity: $Tau^2 = 0$; $Chi^2 = 0.32$, df = 1 (P = 0.57); $I^2 = 0\%$						
**subgroup = Upadacitinib vs Placebo**						
Sandborn et. al,NCT02819635, U-ACHIEVE-induction-SUBSTUDY 1	20	56	1	46	0.4%	16.43 [2.29; 117.82]
Sandborn et. al,NCT02819635, U-ACHIEVE-induction-SUBSTUDY 2	116	319	11	154	2.5%	5.09 [2.83; 9.16]
Sandborn et. al,NCT03653026-U-ACCOMPLISH	150	341	14	174	2.8%	5.47 [3.26; 9.16]
**Total (95% CI)**		**716**		**374**	**5.8%**	**5.53 [3.78; 8.09]**
Heterogeneity: $Tau^2 = 0$; $Chi^2 = 1.26$, df = 2 (P = 0.53); $I^2 = 0\%$						
**subgroup = Ustekinumab vs Placebo**						
Sands et .al,NCT02407236-UNIFI	87	322	44	319	3.7%	1.96 [1.41; 2.72]
**subgroup = Vedolizumab vs Placebo**						
Feagan et. al,NCT00783718-GEMINI I	92	225	37	149	3.8%	1.65 [1.20; 2.27]
Motoya et.al,NCT02039505	60	164	25	82	3.5%	1.20 [0.82; 1.76]
**Total (95% CI)**		**389**		**231**	**7.3%**	**1.43 [1.05; 1.95]**
Heterogeneity: $Tau^2 = 0.02$; $Chi^2 = 1.54$, df = 1 (P = 0.21); $I^2 = 35\%$						
**subgroup = Guselkumab vs Placebo**						
Peyrin-Biroulet et.al,NCT04033435-QUASAR 2b	31	101	13	105	2.5%	2.48 [1.38; 4.46]
Peyrin-Biroulet et.al,NCT04033445-QUASAR 3	113	421	31	280	3.6%	2.42 [1.68; 3.50]
**Total (95% CI)**		**522**		**385**	**6.1%**	**2.44 [1.79; 3.33]**
Heterogeneity: $Tau^2 = 0$; $Chi^2 = 0$, df = 1 (P = 0.95); $I^2 = 0\%$						
**subgroup = Risankizumab vs Placebo**						
Louis et.al,NCT03398148-INSPIRE	237	650	31	325	3.6%	3.82 [2.69; 5.42]
Louis et. al, Phase 2	8	61	3	60	0.9%	2.62 [0.73; 9.42]
**Total (95% CI)**		**711**		**385**	**4.6%**	**3.72 [2.66; 5.22]**
Heterogeneity: $Tau^2 = 0$; $Chi^2 = 0.31$, df = 1 (P = 0.58); $I^2 = 0\%$						
**subgroup = Filgotinib 100 mg vs Placebo**						
Feagan et al,NCT02914522,SELECTION	110	562	39	279	3.7%	1.40 [1.00; 1.96]
**subgroup = Filgotinib 200 mg vs Placebo**						
Feagan et al,NCT02914522,SELECTION	128	507	39	279	3.8%	1.81 [1.30; 2.51]
**Total (95% CI)**		**8091**		**4781**	**100.0%**	**2.02 [1.76; 2.31]**
Heterogeneity: $Tau^2 = 0.10$; $Chi^2 = 114.00$, df = 33 (P < 0.01); $I^2 = 71\%$						
Test for subgroup differences: $Chi^2 = 90.06$, df = 13 (P < 0.01)						

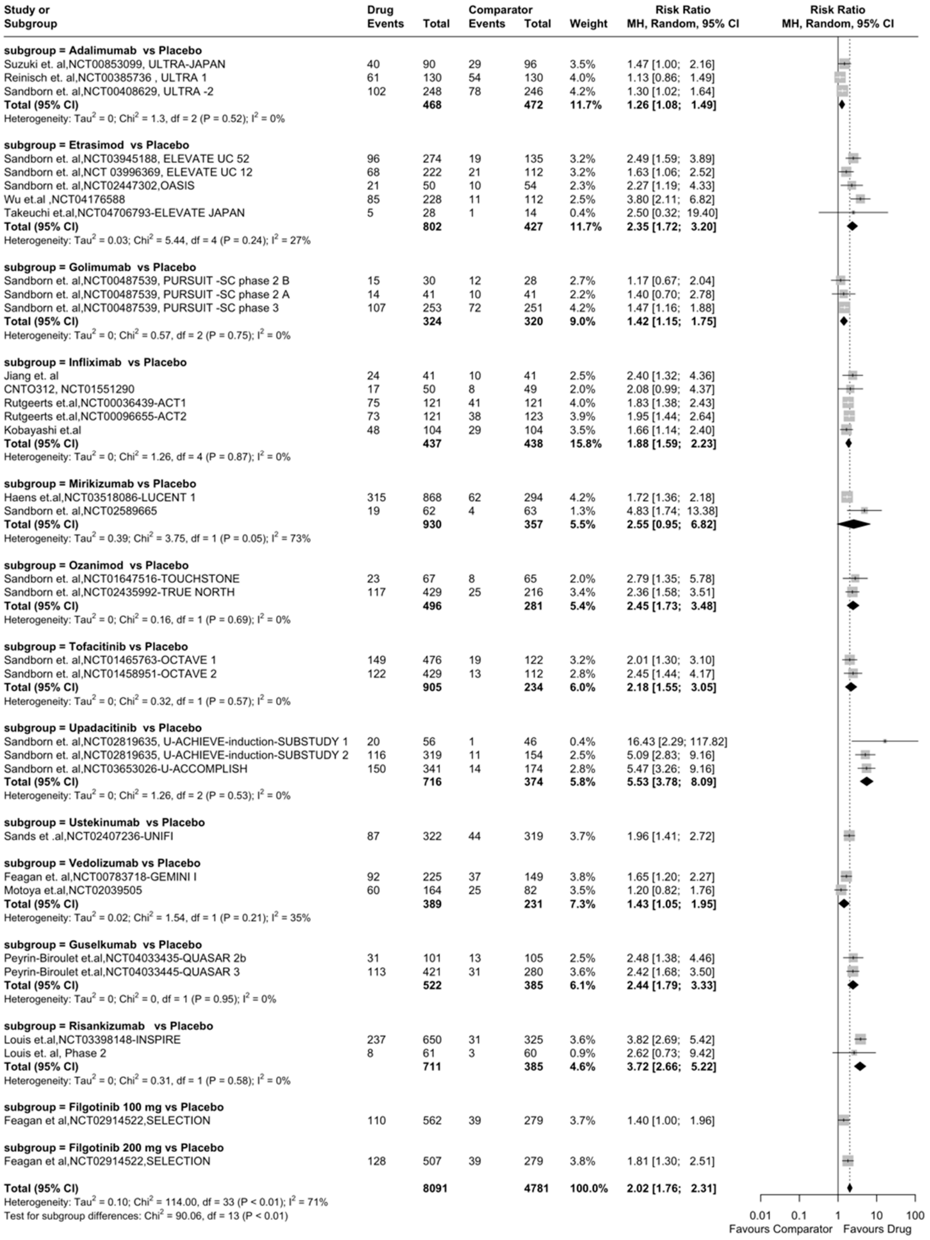

Supplementary Figure S4: pair-wise meta-analysis for maintenance of endoscopic improvement in treat straight through trials (only drugs compared to placebo)

Study or Subgroup	Drug Events	Drug Total	Comparator Events	Comparator Total	Weight	Risk Ratio MH, Random, 95% CI
**subgroup = Adalimumab vs Placebo**						
Suzuki et. al,NCT00853099, ULTRA-JAPAN	51	177	15	96	9.2%	1.84 [1.10; 3.10]
Sandborn et. al,NCT00408629, ULTRA -2	62	248	38	246	14.0%	1.62 [1.13; 2.33]
**Total (95% CI)**		**425**		**342**	**23.2%**	**1.69 [1.25; 2.28]**
Heterogeneity: $Tau^2 = 0$; $Chi^2 = 0.16$, df = 1 (P = 0.69); $I^2 = 0\%$						
**subgroup = Etrasimod vs Placebo**						
Sandborn et. al,NCT03945188, ELEVATE UC 52	99	262	16	135	10.0%	3.19 [1.96; 5.18]
Takeuchi et.al,NCT04706793-ELEVATE JAPAN	8	28	1	14	0.9%	4.00 [0.55; 28.90]
**Total (95% CI)**		**290**		**149**	**10.9%**	**3.23 [2.02; 5.18]**
Heterogeneity: $Tau^2 = 0$; $Chi^2 = 0.05$, df = 1 (P = 0.83); $I^2 = 0\%$						
**subgroup = Golimumab vs Placebo**						
Hibi et.al,NCT01863771, PURSUIT-J	20	32	5	31	4.3%	3.88 [1.66; 9.03]
**subgroup = Infliximab vs Placebo**						
Jiang et. al	22	41	9	41	6.8%	2.44 [1.28; 4.65]
CNTO312, NCT01551290	26	50	13	49	8.8%	1.96 [1.15; 3.35]
Rutgeerts et.al,NCT00036439-ACT1	55	121	22	121	11.8%	2.50 [1.63; 3.83]
Rutgeerts et.al,NCT00096655-ACT2	56	121	37	123	15.3%	1.54 [1.11; 2.14]
Kobayashi et.al	43	104	30	104	13.4%	1.43 [0.98; 2.09]
**Total (95% CI)**		**437**		**438**	**56.0%**	**1.82 [1.45; 2.30]**
Heterogeneity: $Tau^2 = 0.02$; $Chi^2 = 5.55$, df = 4 (P = 0.24); $I^2 = 28\%$						
**subgroup = Ozanimod vs Placebo**						
Sandborn et. al,NCT01647516-TOUCHSTONE	22	67	8	65	5.5%	2.67 [1.28; 5.56]
**Total (95% CI)**		**1251**		**1025**	**100.0%**	**2.03 [1.67; 2.46]**
Heterogeneity: $Tau^2 = 0.03$; $Chi^2 = 15.35$, df = 10 (P = 0.12); $I^2 = 35\%$						
Test for subgroup differences: $Chi^2 = 8.75$, df = 4 (P = 0.07)						

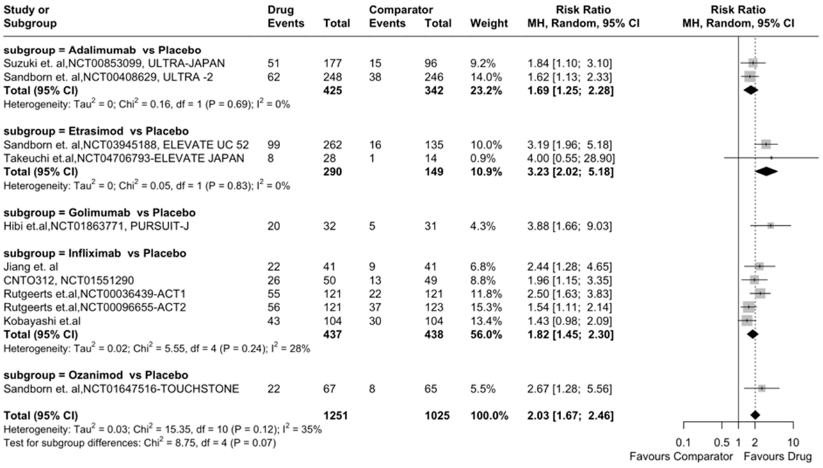

Supplementary Figure S5: pair-wise meta-analysis for maintenance of endoscopic improvement in treat straight through trials

Study or Subgroup	Drug Events	Total	Comparator Events	Total	Weight	Risk Ratio MH, Random, 95% CI
**subgroup = Adalimumab vs Placebo**						
Suzuki et. al,NCT00853099, ULTRA-JAPAN	51	177	15	96	6.9%	1.84 [1.10; 3.10]
Sandborn et. al,NCT00408629, ULTRA -2	62	248	38	246	7.7%	1.62 [1.13; 2.33]
**Total (95% CI)**		**425**		**342**	**14.6%**	**1.69 [1.25; 2.28]**
Heterogeneity: $Tau^2 = 0$; $Chi^2 = 0.16$, df = 1 (P = 0.69); $I^2 = 0\%$						
**subgroup = Etrasimod vs Placebo**						
Sandborn et. al,NCT03945188, ELEVATE UC 52	99	262	16	135	7.1%	3.19 [1.96; 5.18]
Takeuchi et.al,NCT04706793-ELEVATE JAPAN	8	28	1	14	1.8%	4.00 [0.55; 28.90]
**Total (95% CI)**		**290**		**149**	**8.9%**	**3.23 [2.02; 5.18]**
Heterogeneity: $Tau^2 = 0$; $Chi^2 = 0.05$, df = 1 (P = 0.83); $I^2 = 0\%$						
**subgroup = Golimumab vs Placebo**						
Hibi et.al,NCT01863771, PURSUIT-J	20	32	5	31	5.1%	3.88 [1.66; 9.03]
**subgroup = Infliximab vs Placebo**						
Jiang et. al	22	41	9	41	6.2%	2.44 [1.28; 4.65]
CNTO312, NCT01551290	26	50	13	49	6.8%	1.96 [1.15; 3.35]
Rutgeerts et.al,NCT00036439-ACT1	55	121	22	121	7.4%	2.50 [1.63; 3.83]
Rutgeerts et.al,NCT00096655-ACT2	56	121	37	123	7.9%	1.54 [1.11; 2.14]
Kobayashi et.al	43	104	30	104	7.7%	1.43 [0.98; 2.09]
**Total (95% CI)**		**437**		**438**	**36.0%**	**1.82 [1.45; 2.30]**
Heterogeneity: $Tau^2 = 0.02$; $Chi^2 = 5.55$, df = 4 (P = 0.24); $I^2 = 28\%$						
**subgroup = Ozanimod vs Placebo**						
Sandborn et. al,NCT01647516-TOUCHSTONE	22	67	8	65	5.7%	2.67 [1.28; 5.56]
**subgroup = Adalimumab vs Vedolizumab**						
Sands et .al,NCT02497469-VARSITY	107	386	152	383	8.4%	0.70 [0.57; 0.86]
**subgroup = Golimumab/Guselkumab vs Golimumab**						
Sands et .al,NCT03662542-VEGA	33	71	16	72	7.0%	2.09 [1.27; 3.45]
**subgroup = Golimumab/Guselkumab vs Guselkumab**						
Sands et .al,NCT03662542-VEGA	33	71	23	71	7.5%	1.43 [0.94; 2.18]
**subgroup = Golimumab vs Guselkumab**						
Sands et .al,NCT03662542-VEGA	16	72	23	71	6.8%	0.69 [0.40; 1.19]
**Total (95% CI)**		**1851**		**1622**	**100.0%**	**1.76 [1.31; 2.38]**
Heterogeneity: $Tau^2 = 0.27$; $Chi^2 = 89.02$, df = 14 (P < 0.01); $I^2 = 84\%$						
Test for subgroup differences: $Chi^2 = 80.45$, df = 8 (P < 0.01)						

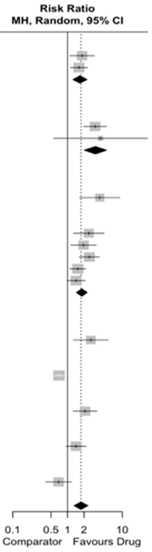

Supplementary Figure S6: pair-wise meta-analysis for maintenance of endoscopic improvement in re-randomization only responders trials (only drugs compared to placebo)

Study or Subgroup	Drug Events	Total	Comparator Events	Total	Weight	Risk Ratio MH, Random, 95% CI
**subgroup = Golimumab vs Placebo**						
Sandborn et. al,NCT00488631, PURSUIT-M,Golimumab responders	64	151	41	154	6.1%	1.59 [1.15; 2.20]
**subgroup = Mirikizumab vs Placebo**						
Haens et.al,NCT03524092-LUCENT 2	214	365	52	179	7.0%	2.02 [1.58; 2.58]
Sandborn et. al,NCT02589665	27	47	2	13	1.1%	3.73 [1.02; 13.68]
**Total (95% CI)**		**412**		**192**	**8.1%**	**2.06 [1.62; 2.62]**
Heterogeneity: $Tau^2 = 0$; $Chi^2 = 0.85$, df = 1 (P = 0.36); $I^2 = 0\%$						
**subgroup = Ozanimod vs Placebo**						
Sandborn et. al,NCT02435992-TRUE NORTH	105	230	60	227	6.9%	1.73 [1.33; 2.24]
**subgroup = Tofacitinib 10 mg vs Placebo**						
Sandborn et. al,NCT01458574-OCTAVE Sustain	90	197	26	198	5.3%	3.48 [2.36; 5.13]
**subgroup = Tofacitinib 5 mg vs Placebo**						
Sandborn et. al,NCT01458574-OCTAVE Sustain	74	198	26	198	5.2%	2.85 [1.91; 4.25]
**subgroup = Upadacitinib 15 mg vs Placebo**						
Sandborn et. al,NCT02819635, U-ACHIEVE-Maintenance -SUBSTUDY 3	72	148	22	149	5.0%	3.29 [2.17; 5.01]
**subgroup = Upadacitinib 30 mg vs Placebo**						
Sandborn et. al,NCT02819635, U-ACHIEVE-Maintenance -SUBSTUDY 3	95	154	22	149	5.2%	4.18 [2.79; 6.27]
**subgroup = Ustekinumab 90 mg vs Placebo**						
Sands et .al,NCT02407236-UNIFI	90	176	50	175	6.7%	1.79 [1.36; 2.36]
**subgroup = Vedolizumab vs Placebo**						
Feagan et. al,NCT00783718-GEMINI I	63	122	25	126	5.3%	2.60 [1.76; 3.85]
Motoya et.al,NCT02039505	26	41	14	42	4.3%	1.90 [1.17; 3.10]
**Total (95% CI)**		**163**		**168**	**9.7%**	**2.30 [1.70; 3.12]**
Heterogeneity: $Tau^2 = 0$; $Chi^2 = 0.99$, df = 1 (P = 0.32); $I^2 = 0\%$						
**subgroup = Vedolizumab SC vs Placebo**						
Sandborn et. al,NCT02611830-VISIBLE I	60	106	12	56	4.0%	2.64 [1.56; 4.48]
**subgroup = Vedolizumab IV vs Placebo**						
Sandborn et. al,NCT02611830-VISIBLE I	29	54	12	56	3.7%	2.51 [1.43; 4.38]
**subgroup = Risankizumab 180 mg SC vs Placebo**						
Louis et.al,NCT03398135-COMMAND	91	179	58	183	6.9%	1.60 [1.24; 2.07]
**subgroup = Risankizumab 360 mg vs Placebo**						
Louis et.al,NCT03398135-COMMAND	90	186	58	183	6.9%	1.53 [1.18; 1.98]
**subgroup = Guselkumab 200mg vs Placebo**						
Peyrin-Biroulet et.al,NCT04033445-QUASAR 3	98	190	36	190	6.1%	2.72 [1.97; 3.77]
**subgroup = Guselkumab 100 mg vs Placebo**						
Peyrin-Biroulet et.al,NCT04033445-QUASAR 3	93	188	36	190	6.0%	2.61 [1.88; 3.62]
**subgroup = Filgotinib 100 mg vs Placebo**						
Feagan et al,NCT02914522,SELECTION Maintenance	46	172	15	98	4.0%	1.75 [1.03; 2.96]
**subgroup = Filgotinib 200 mg vs Placebo**						
Feagan et al,NCT02914522,SELECTION Maintenance	81	199	15	98	4.3%	2.66 [1.62; 4.36]
**Total (95% CI)**		**3103**		**2664**	**100.0%**	**2.27 [1.97; 2.61]**
Heterogeneity: $Tau^2 = 0.06$; $Chi^2 = 50.18$, df = 18 (P < 0.01); $I^2 = 64\%$						
Test for subgroup differences: $Chi^2 = 47.36$, df = 16 (P < 0.01)						

Risk Ratio MH, Random, 95% CI

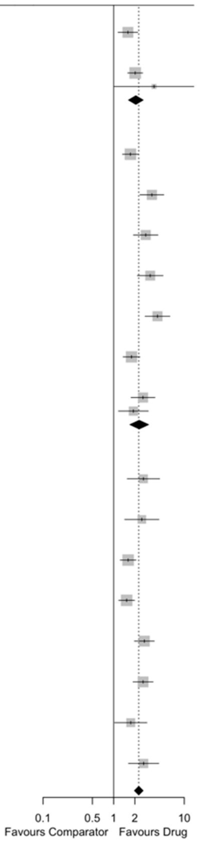

Supplementary Figure S7: pair-wise meta-analysis for maintenance of endoscopic improvement in re-randomization only responders trials

Study or Subgroup	Drug Events	Total	Comparator Events	Total	Weight	Risk Ratio MH, Random, 95% CI
**subgroup = Golimumab vs Placebo**						
Sandborn et. al,NCT00488631, PURSUIT-M,Golimumab responders	64	151	41	154	4.2%	1.59 [1.15; 2.20]
**subgroup = Mirikizumab vs Placebo**						
Haens et.al,NCT03524092-LUCENT 2	214	365	52	179	4.4%	2.02 [1.58; 2.58]
Sandborn et. al,NCT02589665	27	47	2	13	1.5%	3.73 [1.02; 13.68]
**Total (95% CI)**		**412**		**192**	**6.0%**	**2.06 [1.62; 2.62]**
Heterogeneity: Tau^2 = 0; Chi^2 = 0.85, df = 1 (P = 0.36); I^2 = 0%						
**subgroup = Ozanimod vs Placebo**						
Sandborn et. al,NCT02435992-TRUE NORTH	105	230	60	227	4.4%	1.73 [1.33; 2.24]
**subgroup = Tofacitinib 10 mg vs Placebo**						
Sandborn et. al,NCT01458574-OCTAVE Sustain	90	197	26	198	4.0%	3.48 [2.36; 5.13]
**subgroup = Tofacitinib 5 mg vs Placebo**						
Sandborn et. al,NCT01458574-OCTAVE Sustain	74	198	26	198	4.0%	2.85 [1.91; 4.25]
**subgroup = Tofacitinib 10 mg vs Tofacitinib 5 mg**						
Sandborn et. al,NCT01458574-OCTAVE Sustain	90	197	74	198	4.4%	1.22 [0.97; 1.55]
**subgroup = Upadacitinib 15 mg vs Placebo**						
Sandborn et. al,NCT02819635, U-ACHIEVE-Maintenance -SUBSTUDY 3	72	148	22	149	3.9%	3.29 [2.17; 5.01]
**subgroup = Upadacitinib 30 mg vs Placebo**						
Sandborn et. al,NCT02819635, U-ACHIEVE-Maintenance -SUBSTUDY 3	95	154	22	149	3.9%	4.18 [2.79; 6.27]
**subgroup = Upadacitinib 15 mg vs Upadacitinib 30mg**						
Sandborn et. al,NCT02819635, U-ACHIEVE-Maintenance -SUBSTUDY 3	72	148	95	154	4.5%	0.79 [0.64; 0.97]
**subgroup = Ustekinumab 90 mg vs Placebo**						
Sands et .al,NCT02407236-UNIFI	90	176	50	175	4.3%	1.79 [1.36; 2.36]
**subgroup = Vedolizumab vs Placebo**						
Feagan et. al,NCT00783718-GEMINI I	63	122	25	126	4.0%	2.60 [1.76; 3.85]
Motoya et.al,NCT02039505	26	41	14	42	3.7%	1.90 [1.17; 3.10]
**Total (95% CI)**		**163**		**168**	**7.7%**	**2.30 [1.70; 3.12]**
Heterogeneity: Tau^2 = 0; Chi^2 = 0.99, df = 1 (P = 0.32); I^2 = 0%						
**subgroup = Vedolizumab SC vs Placebo**						
Sandborn et. al,NCT02611830-VISIBLE I	60	106	12	56	3.5%	2.64 [1.56; 4.48]
**subgroup = Vedolizumab IV vs Placebo**						
Sandborn et. al,NCT02611830-VISIBLE I	29	54	12	56	3.4%	2.51 [1.43; 4.38]
**subgroup = Vedolizumab SC vs Vedolizumab IV**						
Sandborn et. al,NCT02611830-VISIBLE I	60	106	29	54	4.3%	1.05 [0.78; 1.42]
**subgroup = Risankizumab 180 mg SC vs Placebo**						
Louis et.al,NCT03398135-COMMAND	91	179	58	183	4.4%	1.60 [1.24; 2.07]
**subgroup = Risankizumab 360 mg vs Placebo**						
Louis et.al,NCT03398135-COMMAND	90	186	58	183	4.4%	1.53 [1.18; 1.98]
**subgroup = Risankizumab 180mg vs Risankizumab 360mg**						
Louis et.al,NCT03398135-COMMAND	91	179	90	186	4.5%	1.05 [0.85; 1.29]
**subgroup = Guselkumab 200mg vs Placebo**						
Peyrin-Biroulet et.al,NCT04033445-QUASAR 3	98	190	36	190	4.2%	2.72 [1.97; 3.77]
**subgroup = Guselkumab 100 mg vs Placebo**						
Peyrin-Biroulet et.al,NCT04033445-QUASAR 3	93	188	36	190	4.2%	2.61 [1.88; 3.62]
**subgroup = Guselkumab 200mg vs Guselkumab 100 mg**						
Peyrin-Biroulet et.al,NCT04033445-QUASAR 3	98	190	93	188	4.5%	1.04 [0.85; 1.27]
**subgroup = Filgotinib 100 mg vs Placebo**						
Feagan et al,NCT02914522,SELECTION Maintenance	46	172	15	98	3.5%	1.75 [1.03; 2.96]
**subgroup = Filgotinib 200 mg vs Placebo**						
Feagan et al,NCT02914522,SELECTION Maintenance	81	199	15	98	3.6%	2.66 [1.62; 4.36]
**subgroup = Filgotinib 100 mg vs Filgotinib 200 mg**						
Feagan et al,NCT02914522,SELECTION Maintenance	46	172	81	199	4.3%	0.66 [0.49; 0.89]
**Total (95% CI)**		**4095**		**3643**	**100.0%**	**1.83 [1.50; 2.23]**
Heterogeneity: Tau^2 = 0.21; Chi^2 = 229.73, df = 24 (P < 0.01); I^2 = 90%						
Test for subgroup differences: Chi^2 = 218.64, df = 22 (P < 0.01)						

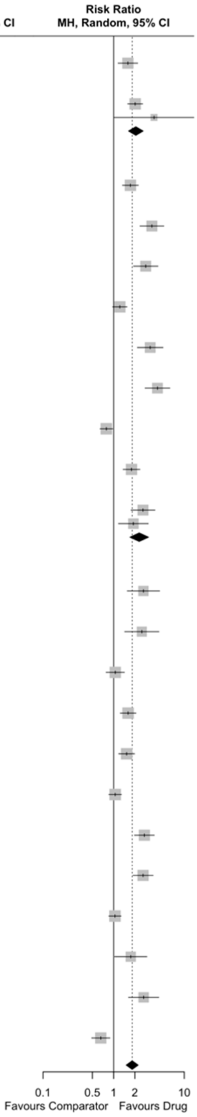

Supplementary Figure S8: pair-wise meta-analysis for induction of mucosal healing (only drugs compared to placebo)

Study or Subgroup	Drug Events	Total	Comparator Events	Total	Weight	Risk Ratio MH, Random, 95% CI
**subgroup = Ozanimod vs Placebo**						
Sandborn et. al,NCT02435992-TRUE NORTH	54	429	8	216	9.4%	3.40 [1.65; 7.01]
**subgroup = Upadacitinib vs Placebo**						
Sandborn et. al,NCT02819635, U-ACHIEVE-induction-SUBSTUDY 2	34	319	2	154	4.2%	8.21 [2.00; 33.72]
Sandborn et. al,NCT03653026-U-ACCOMPLISH	46	341	3	174	5.6%	7.82 [2.47; 24.80]
**Total (95% CI)**		**660**		**328**	**9.9%**	**7.97 [3.26; 19.49]**
Heterogeneity: $Tau^2 = 0$; $Chi^2 = 0$, df = 1 (P = 0.96); $I^2 = 0\%$						
**subgroup = Risankizumab vs Placebo**						
Louis et.al,NCT03398148-INSPIRE	41	650	2	325	4.2%	10.25 [2.49; 42.11]
**subgroup = Etrasimod vs Placebo**						
Sandborn et. al,NCT03945188, ELEVATE UC 52	58	274	6	135	8.4%	4.76 [2.11; 10.76]
Sandborn et. al,NCT 03996369, ELEVATE UC 12	36	222	10	112	10.1%	1.82 [0.94; 3.52]
Wu et.al ,NCT04176588	59	228	5	112	7.8%	5.80 [2.39; 14.04]
Takeuchi et.al,NCT04706793-ELEVATE JAPAN	3	28	0	14	0.9%	5.50 [0.17; 180.06]
**Total (95% CI)**		**752**		**373**	**27.2%**	**3.56 [1.81; 7.02]**
Heterogeneity: $Tau^2 = 0.21$; $Chi^2 = 5.76$, df = 3 (P = 0.12); $I^2 = 48\%$						
**subgroup = Mirikizumab vs Placebo**						
Haens et.al,NCT03518086-LUCENT 1	235	868	41	294	14.7%	1.94 [1.43; 2.63]
**subgroup = Filgotinib 100 mg vs Placebo**						
Feagan et al,NCT02914522,SELECTION	59	562	21	279	12.5%	1.39 [0.87; 2.25]
**subgroup = Filgotinib 200 mg vs Placebo**						
Feagan et al,NCT02914522,SELECTION	83	507	21	279	12.8%	2.17 [1.38; 3.43]
**subgroup = Guselkumab vs Placebo**						
Peyrin-Biroulet et.al,NCT04033435-QUASAR 2b	21	101	9	105	9.3%	2.43 [1.17; 5.04]
**Total (95% CI)**		**4529**		**2199**	**100.0%**	**2.95 [2.11; 4.13]**
Heterogeneity: $Tau^2 = 0.18$; $Chi^2 = 27.90$, df = 11 (P < 0.01); $I^2 = 61\%$						
Test for subgroup differences: $Chi^2 = 19.93$, df = 7 (P < 0.01)						

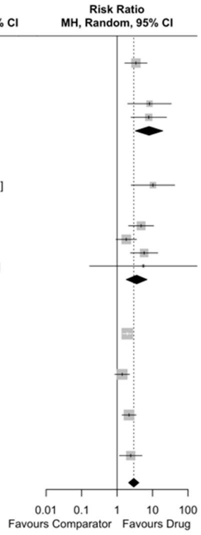

Supplementary Figure S9: pair-wise meta-analysis for maintenance of mucosal healing (only drugs compared to placebo)

Study or Subgroup	Drug Events	Total	Comparator Events	Total	Weight	Risk Ratio MH, Random, 95% CI
**subgroup = Ozanimod vs Placebo**						
Sandborn et. al,NCT02435992-TRUE NORTH	68	230	32	227	8.5%	2.10 [1.44; 3.06]
**subgroup = Upadacitinib 15 mg vs Placebo**						
Sandborn et. al,NCT02819635, U-ACHIEVE-Maintenance -SUBSTUDY 3	26	148	7	149	5.7%	3.74 [1.68; 8.35]
**subgroup = Upadacitinib 30 mg vs Placebo**						
Sandborn et. al,NCT02819635, U-ACHIEVE-Maintenance -SUBSTUDY 3	29	154	7	149	5.7%	4.01 [1.81; 8.87]
**subgroup = Risankizumab 180 mg SC vs Placebo**						
Louis et.al,NCT03398135-COMMAND	23	179	18	183	7.1%	1.31 [0.73; 2.34]
**subgroup = Risankizumab 360 mg vs Placebo**						
Louis et.al,NCT03398135-COMMAND	29	186	18	183	7.3%	1.59 [0.91; 2.75]
**subgroup = Mirikizumab vs Placebo**						
Haens et.al,NCT03524092-LUCENT 2	158	365	39	179	9.0%	1.99 [1.47; 2.69]
**subgroup = Guselkumab 200mg vs Placebo**						
Peyrin-Biroulet et.al,NCT04033445-QUASAR 3	62	190	27	190	8.3%	2.30 [1.53; 3.44]
**subgroup = Guselkumab 100 mg vs Placebo**						
Peyrin-Biroulet et.al,NCT04033445-QUASAR 3	59	188	27	190	8.3%	2.21 [1.47; 3.32]
**subgroup = Guselkumab 200mg vs Guselkumab 100 mg**						
Peyrin-Biroulet et.al,NCT04033445-QUASAR 3	62	190	59	188	9.0%	1.04 [0.77; 1.40]
**subgroup = Filgotinib 100 mg vs Placebo**						
Feagan et al,NCT02914522,SELECTION Maintenance	36	172	10	98	6.6%	2.05 [1.07; 3.95]
**subgroup = Filgotinib 200 mg vs Placebo**						
Feagan et al,NCT02914522,SELECTION Maintenance	65	199	10	98	6.9%	3.20 [1.72; 5.95]
**subgroup = Filgotinib 100 mg vs Filgotinib 200 mg**						
Feagan et al,NCT02914522,SELECTION Maintenance	36	172	65	199	8.7%	0.64 [0.45; 0.91]
**subgroup = Etrasimod vs Placebo**						
Sandborn et. al,NCT03945188, ELEVATE UC 52	71	262	12	135	7.2%	3.05 [1.71; 5.42]
Takeuchi et.al,NCT04706793-ELEVATE JAPAN	7	28	1	14	1.8%	3.50 [0.48; 25.72]
**Total (95% CI)**		**290**		**149**	**8.9%**	**3.08 [1.77; 5.36]**
Heterogeneity: $Tau^2 = 0$; $Chi^2 = 0.02$, df = 1 (P = 0.90); $I^2 = 0\%$						
**Total (95% CI)**		**2663**		**2182**	**100.0%**	**1.94 [1.45; 2.59]**
Heterogeneity: $Tau^2 = 0.22$; $Chi^2 = 64.75$, df = 13 (P < 0.01); $I^2 = 80\%$						
Test for subgroup differences: $Chi^2 = 63.79$, df = 12 (P < 0.01)						

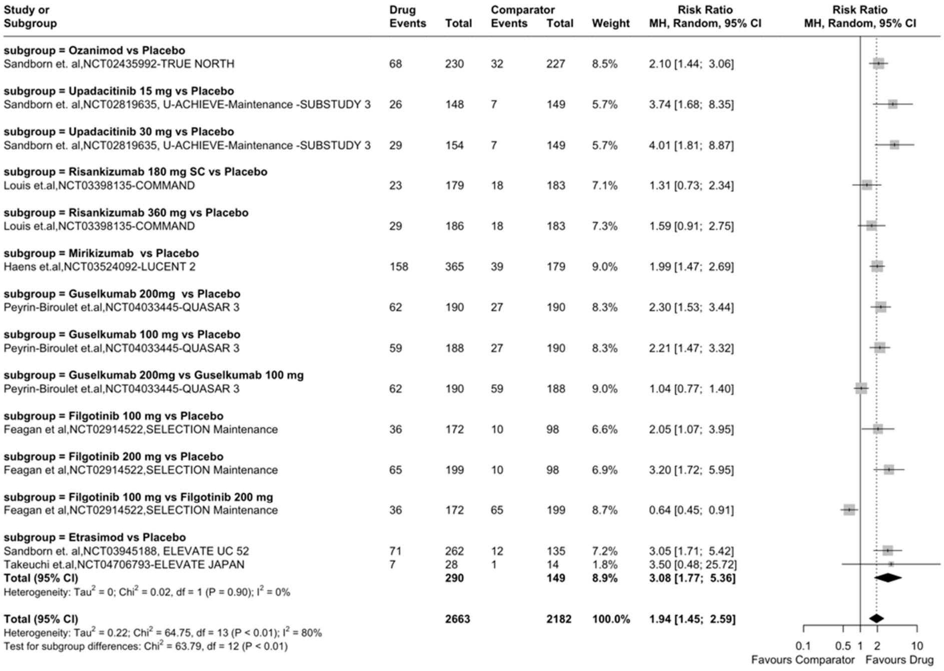

Supplementary Figure S10: pair-wise meta-analysis for maintenance of mucosal healing in treat-straight-through trials(all drugs)

Study or Subgroup	Drug Events	Total	Comparator Events	Total	Weight	Risk Ratio MH, Random, 95% CI
**subgroup = Golimumab/Guselkumab vs Golimumab**						
Sands et .al,NCT03662542-VEGA	17	71	4	72	19.7%	4.31 [1.53; 12.18]
**subgroup = Golimumab/Guselkumab vs Guselkumab**						
Sands et .al,NCT03662542-VEGA	17	71	9	71	24.9%	1.89 [0.90; 3.95]
**subgroup = Golimumab vs Guselkumab**						
Sands et .al,NCT03662542-VEGA	4	72	9	71	18.3%	0.44 [0.14; 1.36]
**subgroup = Etrasimod vs Placebo**						
Sandborn et. al,NCT03945188, ELEVATE UC 52	71	262	12	135	27.9%	3.05 [1.71; 5.42]
Takeuchi et.al,NCT04706793-ELEVATE JAPAN	7	28	1	14	9.2%	3.50 [0.48; 25.72]
**Total (95% CI)**		**290**		**149**	**37.1%**	**3.08 [1.77; 5.36]**
Heterogeneity: $Tau^2 = 0$; $Chi^2 = 0.02$, df = 1 (P = 0.90); $I^2 = 0\%$						
**Total (95% CI)**		**504**		**363**	**100.0%**	**2.06 [1.01; 4.19]**
Heterogeneity: $Tau^2 = 0.38$; $Chi^2 = 11.03$, df = 4 (P = 0.03); $I^2 = 64\%$						
Test for subgroup differences: $Chi^2 = 10.99$, df = 3 (P = 0.01)						

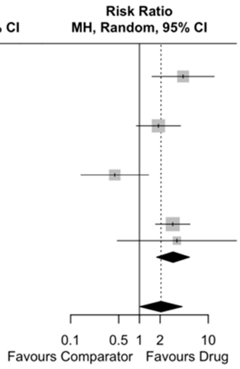

Supplementary Figure S11: pair-wise meta-analysis for maintenance of mucosal healing in re-randomization only responders (all drugs)

Study or Subgroup	Drug Events	Total	Comparator Events	Total	Weight	Risk Ratio MH, Random, 95% CI
**subgroup = Ozanimod vs Placebo**						
Sandborn et. al,NCT02435992-TRUE NORTH	68	230	32	227	7.4%	2.10 [1.44; 3.06]
**subgroup = Upadacitinib 15 mg vs Placebo**						
Sandborn et. al,NCT02819635, U-ACHIEVE-Maintenance -SUBSTUDY 3	26	148	7	149	4.9%	3.74 [1.68; 8.35]
**subgroup = Upadacitinib 30 mg vs Placebo**						
Sandborn et. al,NCT02819635, U-ACHIEVE-Maintenance -SUBSTUDY 3	29	154	7	149	4.9%	4.01 [1.81; 8.87]
**subgroup = Upadacitinib 15 mg vs Upadacitinib 30mg**						
Sandborn et. al,NCT02819635, U-ACHIEVE-Maintenance -SUBSTUDY 3	26	148	29	154	6.8%	0.93 [0.58; 1.51]
**subgroup = Risankizumab 180 mg SC vs Placebo**						
Louis et.al,NCT03398135-COMMAND	23	179	18	183	6.2%	1.31 [0.73; 2.34]
**subgroup = Risankizumab 360 mg vs Placebo**						
Louis et.al,NCT03398135-COMMAND	29	186	18	183	6.4%	1.59 [0.91; 2.75]
**subgroup = Risankizumab 180mg vs Risankizumab 360mg**						
Louis et.al,NCT03398135-COMMAND	23	179	29	186	6.6%	0.82 [0.50; 1.37]
**subgroup = Mirikizumab vs Placebo**						
Haens et.al,NCT03524092-LUCENT 2	158	365	39	179	7.8%	1.99 [1.47; 2.69]
**subgroup = Guselkumab 200mg vs Placebo**						
Peyrin-Biroulet et.al,NCT04033445-QUASAR 3	62	190	27	190	7.3%	2.30 [1.53; 3.44]
**subgroup = Guselkumab 100 mg vs Placebo**						
Peyrin-Biroulet et.al,NCT04033445-QUASAR 3	59	188	27	190	7.2%	2.21 [1.47; 3.32]
**subgroup = Guselkumab 200mg vs Guselkumab 100 mg**						
Peyrin-Biroulet et.al,NCT04033445-QUASAR 3	62	190	59	188	7.9%	1.04 [0.77; 1.40]
**subgroup = CT-P13 vs Placebo**						
Sands et.al,NCT04205643-LIBERTY-UC	105	294	24	144	7.3%	2.14 [1.44; 3.18]
**subgroup = Filgotinib 100 mg vs Placebo**						
Feagan et al,NCT02914522,SELECTION Maintenance	36	172	10	98	5.7%	2.05 [1.07; 3.95]
**subgroup = Filgotinib 200 mg vs Placebo**						
Feagan et al,NCT02914522,SELECTION Maintenance	65	199	10	98	5.9%	3.20 [1.72; 5.95]
**subgroup = Filgotinib 100 mg vs Filgotinib 200 mg**						
Feagan et al,NCT02914522,SELECTION Maintenance	36	172	65	199	7.6%	0.64 [0.45; 0.91]
**Total (95% CI)**		**2994**		**2517**	**100.0%**	**1.69 [1.29; 2.20]**

Heterogeneity: $Tau^2 = 0.21$; $Chi^2 = 72.73$, df = 14 (P < 0.01); $I^2 = 81\%$
Test for subgroup differences: $Chi^2 = 72.05$, df = 14 (P < 0.01)

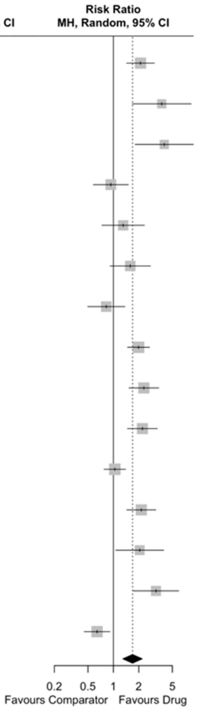

Supplementary Figure S12: pair-wise meta-analysis for induction of endoscopic remission (only drugs compared to placebo)

Study or Subgroup	Drug Events	Total	Comparator Events	Total	Weight	Risk Ratio MH, Random, 95% CI
**subgroup = Filgotinib 100 mg vs Placebo**						
Feagan et al,NCT02914522,SELECTION	53	562	20	279	10.4%	1.32 [0.80; 2.16]
**subgroup = Filgotinib 200 mg vs Placebo**						
Feagan et al,NCT02914522,SELECTION	69	507	20	279	10.6%	1.90 [1.18; 3.06]
**subgroup = Mirikizumab vs Placebo**						
Sandborn et. al,NCT02589665	2	62	1	63	1.8%	2.03 [0.19; 21.84]
**subgroup = Tofacitinib vs Placebo**						
Sandborn et. al,NCT00787202	10	33	1	48	2.4%	14.55 [1.95; 108.26]
Sandborn et. al,NCT01465763-OCTAVE 1	32	476	2	122	4.1%	4.10 [1.00; 16.88]
Sandborn et. al,NCT01458951-OCTAVE 2	30	429	2	112	4.1%	3.92 [0.95; 16.14]
**Total (95% CI)**		**938**		**282**	**10.6%**	**5.18 [2.12; 12.69]**
Heterogeneity: $Tau^2 = 0$; $Chi^2 = 1.27$, df = 2 (P = 0.53); $I^2 = 0\%$						
**subgroup = Upadacitinib vs Placebo**						
Sandborn et. al,NCT02819635, U-ACHIEVE-induction-SUBSTUDY 1	10	56	0	46	1.2%	19.21 [1.00; 368.92]
Sandborn et. al,NCT02819635, U-ACHIEVE-induction-SUBSTUDY 2	44	319	2	154	4.1%	10.62 [2.61; 43.24]
Sandborn et. al,NCT03653026-U-ACCOMPLISH	62	341	3	174	5.4%	10.55 [3.36; 33.11]
**Total (95% CI)**		**716**		**374**	**10.7%**	**11.11 [4.75; 25.98]**
Heterogeneity: $Tau^2 = 0$; $Chi^2 = 0.14$, df = 2 (P = 0.93); $I^2 = 0\%$						
**subgroup = Guselkumab vs Placebo**						
Peyrin-Biroulet et.al,NCT04033435-QUASAR 2b	18	101	7	105	7.5%	2.67 [1.17; 6.13]
Peyrin-Biroulet et.al,NCT04033445-QUASAR 3	63	421	14	280	9.8%	2.99 [1.71; 5.23]
**Total (95% CI)**		**522**		**385**	**17.3%**	**2.89 [1.82; 4.59]**
Heterogeneity: $Tau^2 = 0$; $Chi^2 = 0.05$, df = 1 (P = 0.82); $I^2 = 0\%$						
**subgroup = Risankizumab vs Placebo**						
Louis et.al,NCT03398148-INSPIRE	69	650	11	325	9.3%	3.14 [1.68; 5.84]
Louis et. al, Phase 2	3	61	0	60	1.2%	6.95 [0.36; 133.13]
**Total (95% CI)**		**711**		**385**	**10.5%**	**3.24 [1.76; 5.96]**
Heterogeneity: $Tau^2 = 0$; $Chi^2 = 0.27$, df = 1 (P = 0.61); $I^2 = 0\%$						
**subgroup = Etrasimod vs Placebo**						
Sandborn et. al,NCT03945188, ELEVATE UC 52	40	274	6	135	7.5%	3.28 [1.43; 7.56]
Sandborn et. al,NCT 03996369, ELEVATE UC 12	38	223	9	103	8.7%	1.95 [0.98; 3.88]
Wu et.al ,NCT04176588	35	228	3	112	5.3%	5.73 [1.80; 18.23]
**Total (95% CI)**		**725**		**350**	**21.5%**	**2.92 [1.63; 5.24]**
Heterogeneity: $Tau^2 = 0.08$; $Chi^2 = 2.76$, df = 2 (P = 0.25); $I^2 = 28\%$						
**subgroup = Infliximab vs Placebo**						
Probert et. al,	6	23	6	20	6.5%	0.87 [0.33; 2.27]
**Total (95% CI)**		**4766**		**2417**	**100.0%**	**2.97 [2.11; 4.19]**
Heterogeneity: $Tau^2 = 0.23$; $Chi^2 = 34.94$, df = 16 (P < 0.01); $I^2 = 54\%$						
Test for subgroup differences: $Chi^2 = 28.19$, df = 8 (P < 0.01)						

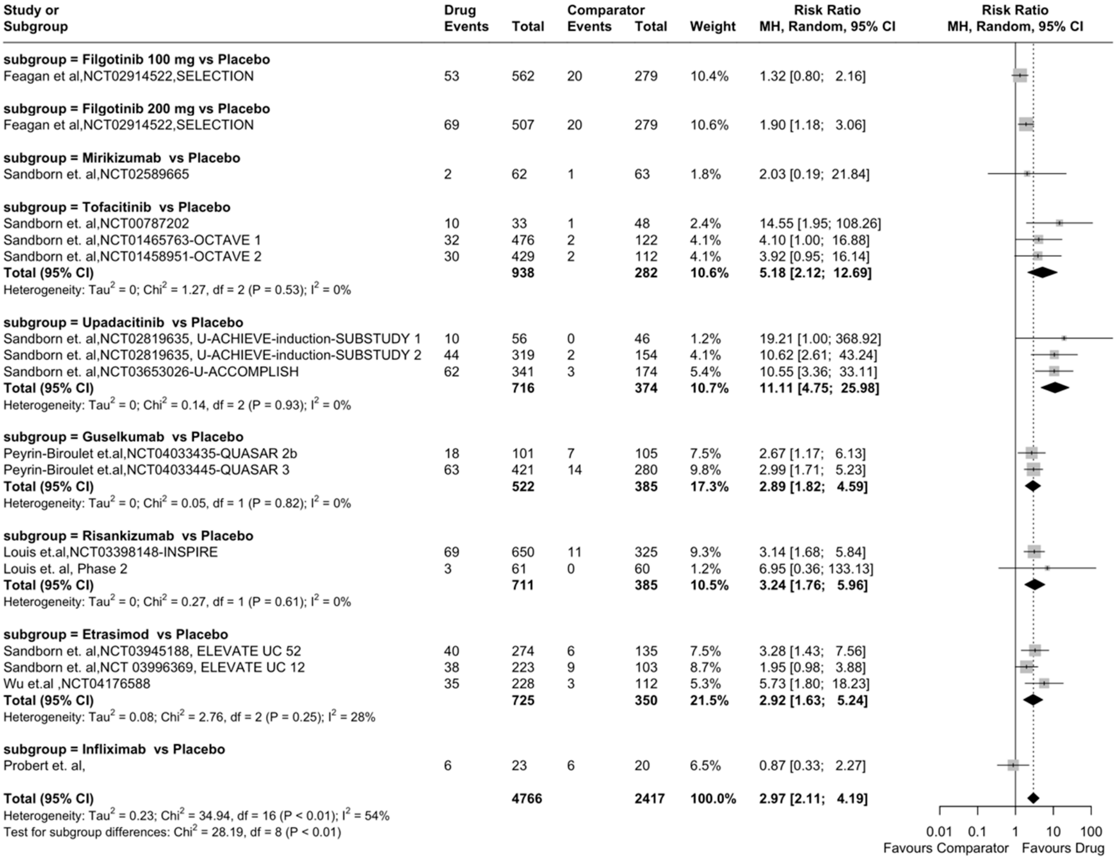

Supplementary Figure S13: pair-wise meta-analysis for induction of endoscopic remission (all drugs)

Study or Subgroup	Drug Events	Total	Comparator Events	Total	Weight	Risk Ratio MH, Random, 95% CI
**subgroup = Filgotinib 100 mg vs Placebo**						
Feagan et al,NCT02914522,SELECTION	53	562	20	279	7.1%	1.32 [0.80; 2.16]
**subgroup = Filgotinib 200 mg vs Placebo**						
Feagan et al,NCT02914522,SELECTION	69	507	20	279	7.2%	1.90 [1.18; 3.06]
**subgroup = Filgotinib 100 mg vs Filgotinib 200 mg**						
Feagan et al,NCT02914522,SELECTION	53	562	60	507	7.7%	0.80 [0.56; 1.13]
**subgroup = Mirikizumab vs Placebo**						
Sandborn et. al,NCT02589665	2	62	1	63	1.7%	2.03 [0.19; 21.84]
**subgroup = Golimumab/Guselkumab vs Golimumab**						
Sands et .al,NCT03662542-VEGA	13	71	7	72	5.6%	1.88 [0.80; 4.44]
**subgroup = Golimumab/Guselkumab vs Guselkumab**						
Sands et .al,NCT03662542-VEGA	13	71	6	71	5.3%	2.17 [0.87; 5.38]
**subgroup = Golimumab vs Guselkumab**						
Sands et .al,NCT03662542-VEGA	7	72	6	71	4.8%	1.15 [0.41; 3.25]
**subgroup = Tofacitinib vs Placebo**						
Sandborn et. al,NCT00787202	10	33	1	48	2.3%	14.55 [1.95; 108.26]
Sandborn et. al,NCT01465763-OCTAVE 1	32	476	2	122	3.6%	4.10 [1.00; 16.88]
Sandborn et. al,NCT01458951-OCTAVE 2	30	429	2	112	3.6%	3.92 [0.95; 16.14]
**Total (95% CI)**		**938**		**282**	**9.4%**	**5.18 [2.12; 12.69]**
Heterogeneity: $Tau^2 = 0$; $Chi^2 = 1.27$, df = 2 (P = 0.53); $I^2 = 0\%$						
**subgroup = Upadacitinib vs Placebo**						
Sandborn et. al,NCT02819635, U-ACHIEVE-induction-SUBSTUDY 1	10	56	0	46	1.2%	19.21 [1.00; 368.92]
Sandborn et. al,NCT02819635, U-ACHIEVE-induction-SUBSTUDY 2	44	319	2	154	3.6%	10.62 [2.61; 43.24]
Sandborn et. al,NCT03653026-U-ACCOMPLISH	62	341	3	174	4.4%	10.55 [3.36; 33.11]
**Total (95% CI)**		**716**		**374**	**9.2%**	**11.11 [4.75; 25.98]**
Heterogeneity: $Tau^2 = 0$; $Chi^2 = 0.14$, df = 2 (P = 0.93); $I^2 = 0\%$						
**subgroup = Guselkumab vs Placebo**						
Peyrin-Biroulet et.al,NCT04033435-QUASAR 2b	18	101	7	105	5.7%	2.67 [1.17; 6.13]
Peyrin-Biroulet et.al,NCT04033445-QUASAR 3	63	421	14	280	6.9%	2.99 [1.71; 5.23]
**Total (95% CI)**		**522**		**385**	**12.6%**	**2.89 [1.82; 4.59]**
Heterogeneity: $Tau^2 = 0$; $Chi^2 = 0.05$, df = 1 (P = 0.82); $I^2 = 0\%$						
**subgroup = Risankizumab vs Placebo**						
Louis et.al,NCT03398148-INSPIRE	69	650	11	325	6.6%	3.14 [1.68; 5.84]
Louis et. al, Phase 2	3	61	0	60	1.2%	6.95 [0.36; 133.13]
**Total (95% CI)**		**711**		**385**	**7.8%**	**3.24 [1.76; 5.96]**
Heterogeneity: $Tau^2 = 0$; $Chi^2 = 0.27$, df = 1 (P = 0.61); $I^2 = 0\%$						
**subgroup = Etrasimod vs Placebo**						
Sandborn et. al,NCT03945188, ELEVATE UC 52	40	274	6	135	5.7%	3.28 [1.43; 7.56]
Sandborn et. al,NCT 03996369, ELEVATE UC 12	38	223	9	103	6.3%	1.95 [0.98; 3.88]
Wu et.al ,NCT04176588	35	228	3	112	4.4%	5.73 [1.80; 18.23]
**Total (95% CI)**		**725**		**350**	**16.4%**	**2.92 [1.63; 5.24]**
Heterogeneity: $Tau^2 = 0.08$; $Chi^2 = 2.76$, df = 2 (P = 0.25); $I^2 = 28\%$						
**subgroup = Infliximab vs Placebo**						
Probert et. al,	6	23	6	20	5.1%	0.87 [0.33; 2.27]
**Total (95% CI)**		**5542**		**3138**	**100.0%**	**2.55 [1.79; 3.64]**
Heterogeneity: $Tau^2 = 0.39$; $Chi^2 = 68.03$, df = 20 (P < 0.01); $I^2 = 71\%$						
Test for subgroup differences: $Chi^2 = 58.81$, df = 12 (P < 0.01)						

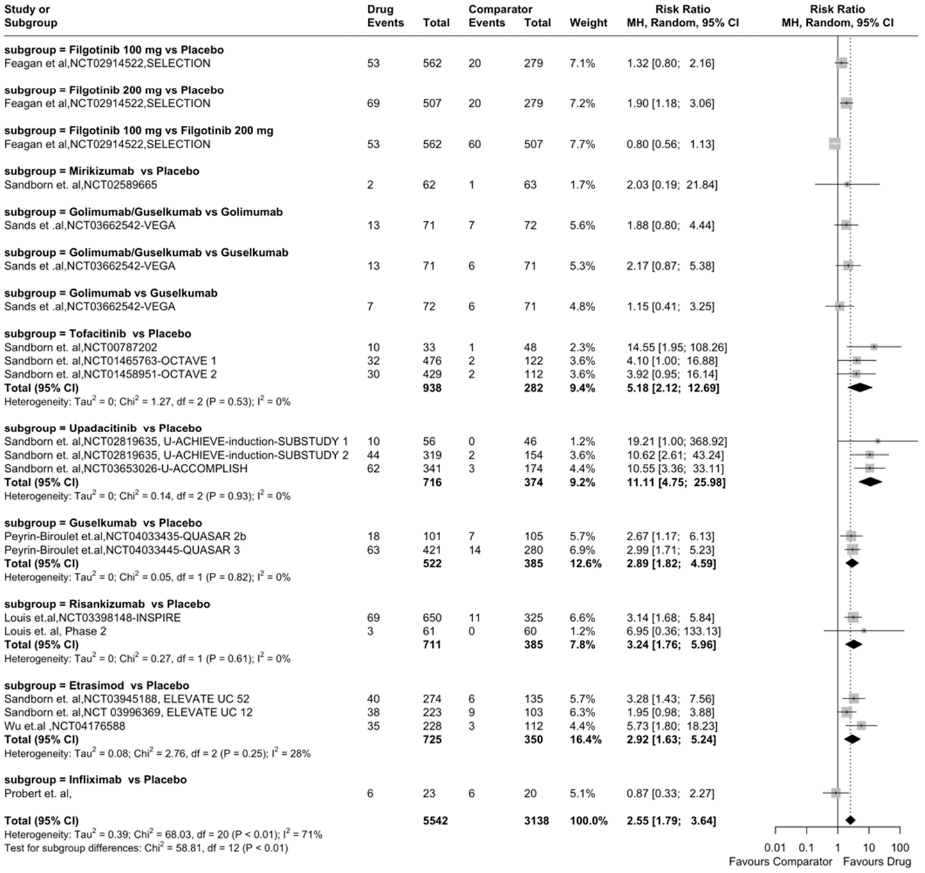

Supplementary Figure S14: pair-wise meta-analysis for maintenance of endoscopic remission (only drugs compared to placebo)

Study or Subgroup	Drug Events	Total	Comparator Events	Total	Weight	Risk Ratio MH, Random, 95% CI
**subgroup = Filgotinib 100 mg vs Placebo**						
Feagan et al,NCT02914522,SELECTION Maintenance	23	172	6	98	5.7%	2.18 [0.92; 5.18]
**subgroup = Filgotinib 200 mg vs Placebo**						
Feagan et al,NCT02914522,SELECTION Maintenance	31	199	6	98	5.9%	2.54 [1.10; 5.89]
**subgroup = Tofacitinib 10 mg vs Placebo**						
Sandborn et. al,NCT01458574-OCTAVE Sustain	33	197	8	198	7.0%	4.15 [1.96; 8.75]
**subgroup = Tofacitinib 5 mg vs Placebo**						
Sandborn et. al,NCT01458574-OCTAVE Sustain	29	198	8	198	6.8%	3.62 [1.70; 7.73]
**subgroup = Upadacitinib 15 mg vs Placebo**						
Sandborn et. al,NCT02819635, U-ACHIEVE-Maintenance -SUBSTUDY 3	36	148	8	149	7.2%	4.53 [2.18; 9.42]
**subgroup = Upadacitinib 30 mg vs Placebo**						
Sandborn et. al,NCT02819635, U-ACHIEVE-Maintenance -SUBSTUDY 3	40	154	8	149	7.2%	4.84 [2.34; 9.99]
**subgroup = Mirikizumab 200 mg vs Placebo**						
Sandborn et. al,NCT02589665	7	47	1	13	1.4%	1.94 [0.26; 14.35]
**subgroup = Risankizumab 180 mg vs Placebo**						
Louis et.al,NCT03398135-COMMAND	41	179	27	183	12.2%	1.55 [1.00; 2.41]
**subgroup = Risankizumab 360 mg vs Placebo**						
Louis et.al,NCT03398135-COMMAND	45	186	27	183	12.4%	1.64 [1.07; 2.52]
**subgroup = Guselkumab 200mg vs Placebo**						
Peyrin-Biroulet et.al,NCT04033445-QUASAR 3	64	190	29	190	13.3%	2.21 [1.49; 3.26]
**subgroup = Guselkumab 100 mg vs Placebo**						
Peyrin-Biroulet et.al,NCT04033445-QUASAR 3	65	188	29	190	13.3%	2.27 [1.54; 3.34]
**subgroup = Etrasimod vs Placebo**						
Sandborn et. al,NCT03945188, ELEVATE UC 52	72	274	8	135	7.6%	4.43 [2.20; 8.94]
**Total (95% CI)**		**2132**		**1784**	**100.0%**	**2.61 [2.05; 3.34]**

Heterogeneity: $Tau^2 = 0.08$; $Chi^2 = 20.31$, df = 11 (P = 0.04); $I^2 = 46\%$
Test for subgroup differences: $Chi^2 = 19.77$, df = 11 (P = 0.05)

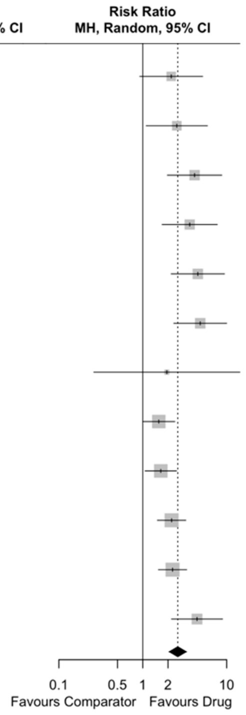

Supplementary Figure S15: pair-wise meta-analysis for maintenance of endoscopic remission (all drugs)

Study or Subgroup	Drug Events	Total	Comparator Events	Total	Weight	Risk Ratio MH, Random, 95% CI
**subgroup = Filgotinib 100 mg vs Placebo**						
Feagan et al,NCT02914522,SELECTION Maintenance	23	172	6	98	4.1%	2.18 [0.92; 5.18]
**subgroup = Filgotinib 200 mg vs Placebo**						
Feagan et al,NCT02914522,SELECTION Maintenance	31	199	6	98	4.2%	2.54 [1.10; 5.89]
**subgroup = Filgotinib 100 mg vs Filgotinib 200 mg**						
Feagan et al,NCT02914522,SELECTION Maintenance	23	172	31	199	5.7%	0.86 [0.52; 1.41]
**subgroup = Tofacitinib 10 mg vs Placebo**						
Sandborn et. al,NCT01458574-OCTAVE Sustain	33	197	8	198	4.6%	4.15 [1.96; 8.75]
**subgroup = Tofacitinib 5 mg vs Placebo**						
Sandborn et. al,NCT01458574-OCTAVE Sustain	29	198	8	198	4.5%	3.62 [1.70; 7.73]
**subgroup = Tofacitinib 10 mg vs Tofacitinib 5 mg**						
Sandborn et. al,NCT01458574-OCTAVE Sustain	33	197	29	198	5.9%	1.14 [0.72; 1.81]
**subgroup = Upadacitinib 15 mg vs Placebo**						
Sandborn et. al,NCT02819635, U-ACHIEVE-Maintenance -SUBSTUDY 3	36	148	8	149	4.6%	4.53 [2.18; 9.42]
**subgroup = Upadacitinib 30 mg vs Placebo**						
Sandborn et. al,NCT02819635, U-ACHIEVE-Maintenance -SUBSTUDY 3	40	154	8	149	4.7%	4.84 [2.34; 9.99]
**subgroup = Upadacitinib 15 mg vs Upadacitinib 30mg**						
Sandborn et. al,NCT02819635, U-ACHIEVE-Maintenance -SUBSTUDY 3	36	148	40	154	6.2%	0.94 [0.63; 1.38]
**subgroup = Mirikizumab 200 mg vs Placebo**						
Sandborn et. al,NCT02589665	7	47	1	13	1.4%	1.94 [0.26; 14.35]
**subgroup = Risankizumab 180 mg vs Placebo**						
Louis et.al,NCT03398135-COMMAND	41	179	27	183	6.0%	1.55 [1.00; 2.41]
**subgroup = Risankizumab 360 mg vs Placebo**						
Louis et.al,NCT03398135-COMMAND	45	186	27	183	6.0%	1.64 [1.07; 2.52]
**subgroup = Risankizumab 180mg vs Risankizumab 360mg**						
Louis et.al,NCT03398135-COMMAND	41	179	45	186	6.3%	0.95 [0.65; 1.37]
**subgroup = Guselkumab 200mg vs Placebo**						
Peyrin-Biroulet et.al,NCT04033445-QUASAR 3	64	190	29	190	6.2%	2.21 [1.49; 3.26]
**subgroup = Guselkumab 100 mg vs Placebo**						
Peyrin-Biroulet et.al,NCT04033445-QUASAR 3	65	188	29	190	6.2%	2.27 [1.54; 3.34]
**subgroup = Guselkumab 200mg vs Guselkumab 100 mg**						
Peyrin-Biroulet et.al,NCT04033445-QUASAR 3	64	190	65	188	6.6%	0.97 [0.74; 1.29]
**subgroup = Golimumab/Guselkumab vs Golimumab**						
Sands et .al,NCT03662542-VEGA	18	71	5	72	3.8%	3.65 [1.43; 9.30]
**subgroup = Golimumab/Guselkumab vs Guselkumab**						
Sands et .al,NCT03662542-VEGA	18	71	11	71	4.9%	1.64 [0.83; 3.21]
**subgroup = Golimumab vs Guselkumab**						
Sands et .al,NCT03662542-VEGA	5	72	11	71	3.5%	0.45 [0.16; 1.22]
**subgroup = Etrasimod vs Placebo**						
Sandborn et. al,NCT03945188, ELEVATE UC 52	72	274	8	135	4.8%	4.43 [2.20; 8.94]
**Total (95% CI)**		**3232**		**2923**	**100.0%**	**1.84 [1.41; 2.40]**

Heterogeneity: $Tau^2 = 0.26$; $Chi^2 = 89.21$, df = 19 ($P < 0.01$); $I^2 = 79\%$
Test for subgroup differences: $Chi^2 = 86.68$, df = 19 ($P < 0.01$)

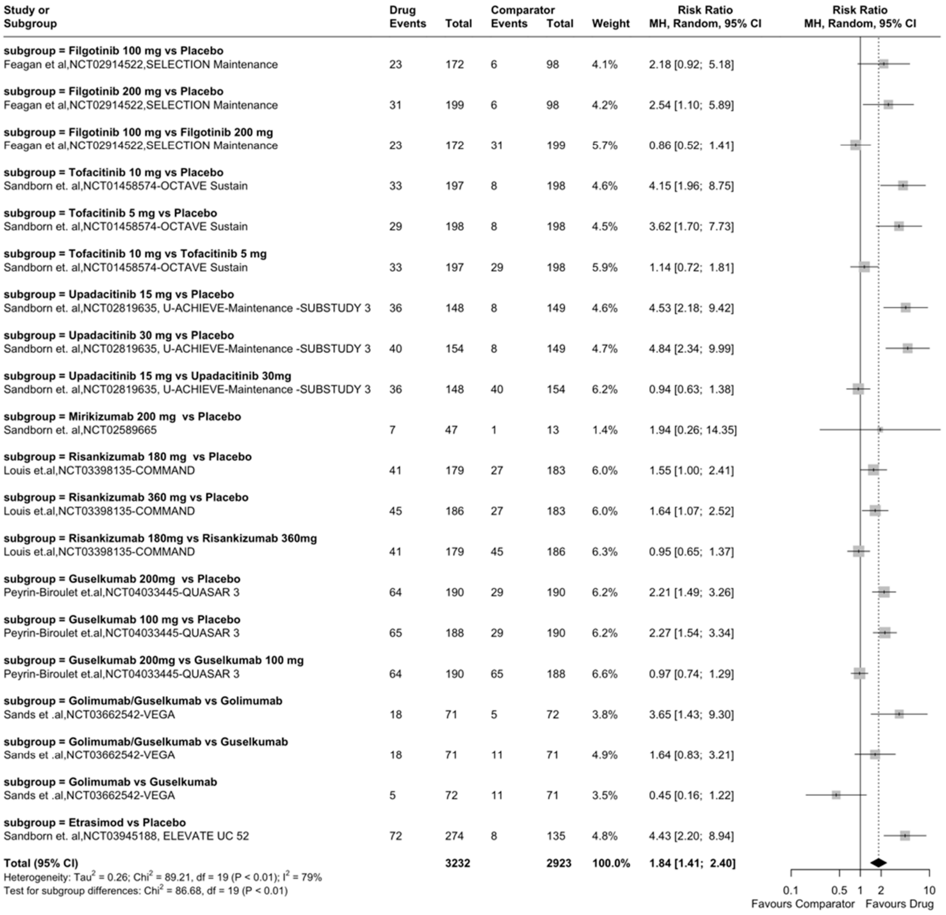

Supplementary Figure S16: pair-wise meta-analysis for induction of histologic improvement

Study or Subgroup	Drug Events	Total	Comparator Events	Total	Weight	Risk Ratio MH, Random, 95% CI	Risk Ratio MH, Random, 95% CI
**subgroup = Etrasimod vs Placebo**							
Sandborn et. al,NCT02447302,OASIS	13	41	5	49	5.7%	3.11 [1.21; 7.99]	
**subgroup = Upadacitinib vs Placebo**							
Sandborn et. al,NCT02819635, U-ACHIEVE-induction-SUBSTUDY 1	27	56	3	46	4.1%	7.39 [2.39; 22.82]	
Sandborn et. al,NCT02819635, U-ACHIEVE-induction-SUBSTUDY 2	175	319	36	154	28.7%	2.35 [1.73; 3.18]	
Sandborn et. al,NCT03653026-U-ACCOMPLISH	212	341	43	174	31.5%	2.52 [1.92; 3.30]	
**Total (95% CI)**		**716**		**374**	**64.3%**	**2.63 [1.92; 3.62]**	
Heterogeneity: Tau^2 = 0.03; Chi^2 = 3.77, df = 2 (P = 0.15); I^2 = 47%							
**subgroup = Ustekinumab vs Placebo**							
Sands et .al,NCT02407236-UNIFI	105	322	55	319	30.1%	1.89 [1.42; 2.52]	
**Total (95% CI)**		**1079**		**742**	**100.0%**	**2.39 [1.89; 3.03]**	
Heterogeneity: Tau^2 = 0.03; Chi^2 = 6.80, df = 4 (P = 0.15); I^2 = 41%							
Test for subgroup differences: Chi^2 = 2.80, df = 2 (P = 0.25)							

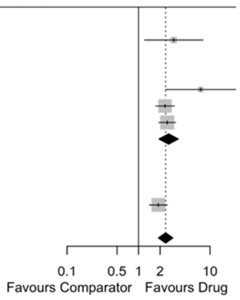

Supplementary Figure S17: pair-wise meta-analysis for maintenance of histologic improvement

Study or Subgroup	Drug Events	Total	Comparator Events	Total	Weight	Risk Ratio MH, Random, 95% CI
**subgroup = Ustekinumab vs Placebo**						
Sands et .al,NCT02407236-UNIFI	99	176	55	175	19.9%	1.79 [1.39; 2.31]
**subgroup = Guselkumab 200mg vs Placebo**						
Peyrin-Biroulet et.al,NCT04033445-QUASAR 3	122	190	58	190	20.0%	2.10 [1.66; 2.67]
**subgroup = Guselkumab 100 mg vs Placebo**						
Peyrin-Biroulet et.al,NCT04033445-QUASAR 3	122	188	58	190	20.0%	2.13 [1.67; 2.70]
**subgroup = Guselkumab 200mg vs Guselkumab 100 mg**						
Peyrin-Biroulet et.al,NCT04033445-QUASAR 3	122	190	122	188	20.5%	0.99 [0.85; 1.15]
**subgroup = Adalimumab vs Vedolizumab**						
Sands et .al,NCT02497469-VARSITY	53	386	128	383	19.6%	0.41 [0.31; 0.55]
**Total (95% CI)**		**1130**		**1126**	**100.0%**	**1.27 [0.75; 2.16]**

Heterogeneity: $Tau^2 = 0.35$; $Chi^2 = 111.96$, df = 4 ($P < 0.01$); $I^2 = 96\%$
Test for subgroup differences: $Chi^2 = 111.82$, df = 4 ($P < 0.01$)

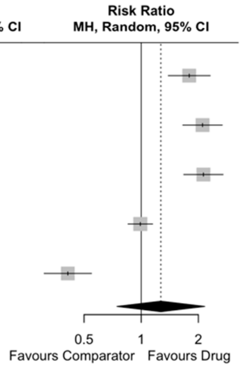

Supplementary Figure S18: pair-wise meta-analysis for induction of histologic remission

Study or Subgroup	Drug Events	Total	Comparator Events	Total	Weight	Risk Ratio MH, Random, 95% CI
**subgroup = Mirikizumab vs Placebo**						
Sandborn et. al,NCT02589665	28	62	11	63	10.4%	2.59 [1.42; 4.73]
**subgroup = Golimumab/Guselkumab vs Golimumab**						
Sands et .al,NCT03662542-VEGA	40	71	23	72	12.2%	1.76 [1.19; 2.62]
**subgroup = Golimumab/Guselkumab vs Guselkumab**						
Sands et .al,NCT03662542-VEGA	40	71	35	71	12.9%	1.14 [0.84; 1.56]
**subgroup = Golimumab vs Guselkumab**						
Sands et .al,NCT03662542-VEGA	23	72	35	71	12.1%	0.65 [0.43; 0.98]
**subgroup = Filgotinib 100 mg vs Placebo**						
Feagan et al,NCT02914522,SELECTION	105	562	34	279	12.5%	1.53 [1.07; 2.20]
**subgroup = Filgotinib 200 mg vs Placebo**						
Feagan et al,NCT02914522,SELECTION	138	507	34	279	12.6%	2.23 [1.58; 3.16]
**subgroup = Filgotinib 100 mg vs Filgotinib 200 mg**						
Feagan et al,NCT02914522,SELECTION	105	562	138	507	13.4%	0.69 [0.55; 0.86]
**subgroup = Etrasimod vs Placebo**						
Sandborn et. al,NCT02447302,OASIS	8	41	3	49	5.5%	3.19 [0.90; 11.24]
**subgroup = Ozanimod vs Placebo**						
Sandborn et. al,NCT01647516-TOUCHSTONE	15	67	7	65	8.4%	2.08 [0.91; 4.77]
**Total (95% CI)**		**2015**		**1456**	**100.0%**	**1.44 [0.98; 2.10]**

Heterogeneity: $Tau^2 = 0.26$; $Chi^2 = 60.95$, df = 8 ($P < 0.01$); $I^2 = 87\%$
Test for subgroup differences: $Chi^2 = 60.47$, df = 8 ($P < 0.01$)

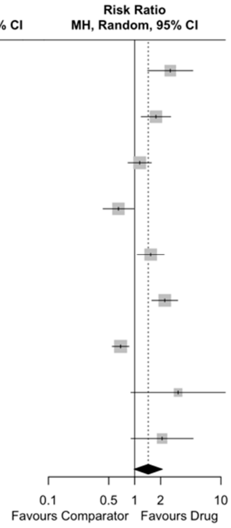

Supplementary Figure S19: pair-wise meta-analysis for maintenance of histologic remission

Study or Subgroup	Drug Events	Total	Comparator Events	Total	Weight	Risk Ratio MH, Random, 95% CI
**subgroup = Filgotinib 100 mg vs Placebo**						
Feagan et al,NCT02914522,SELECTION Maintenance	48	172	13	98	7.8%	2.10 [1.20; 3.68]
**subgroup = Filgotinib 200 mg vs Placebo**						
Feagan et al,NCT02914522,SELECTION Maintenance	76	199	13	98	8.0%	2.88 [1.68; 4.92]
**subgroup = Filgotinib 100 mg vs Filgotinib 200 mg**						
Feagan et al,NCT02914522,SELECTION Maintenance	48	172	76	199	9.3%	0.73 [0.54; 0.98]
**subgroup = Mirikizumab vs Placebo**						
Sandborn et. al,NCT02589665	31	47	5	13	6.8%	1.71 [0.84; 3.51]
**subgroup = Guselkumab 200mg vs Placebo**						
Peyrin-Biroulet et.al,NCT04033445-QUASAR 3	115	190	51	190	9.5%	2.25 [1.74; 2.93]
**subgroup = Guselkumab 100 mg vs Placebo**						
Peyrin-Biroulet et.al,NCT04033445-QUASAR 3	111	188	51	190	9.5%	2.20 [1.69; 2.86]
**subgroup = Guselkumab 200mg vs Guselkumab 100 mg**						
Peyrin-Biroulet et.al,NCT04033445-QUASAR 3	115	190	111	188	9.8%	1.03 [0.87; 1.21]
**subgroup = Golimumab/Guselkumab vs Golimumab**						
Sands et .al,NCT03662542-VEGA	36	71	18	72	8.4%	2.03 [1.28; 3.22]
**subgroup = Golimumab/Guselkumab vs Guselkumab**						
Sands et .al,NCT03662542-VEGA	36	71	29	71	9.0%	1.24 [0.86; 1.78]
**subgroup = Golimumab vs Guselkumab**						
Sands et .al,NCT03662542-VEGA	18	72	29	71	8.3%	0.61 [0.38; 1.00]
**subgroup = Adalimumab vs Vedolizumab**						
Sands et .al,NCT02497469-VARSITY	12	386	40	383	7.4%	0.30 [0.16; 0.56]
**subgroup = Ozanimod vs Placebo**						
Sandborn et. al,NCT01647516-TOUCHSTONE	21	67	6	65	6.1%	3.40 [1.46; 7.87]
**Total (95% CI)**		**1825**		**1638**	**100.0%**	**1.39 [1.00; 1.93]**

Heterogeneity: $Tau^2 = 0.28$; $Chi^2 = 105.86$, df = 11 ($P < 0.01$); $I^2 = 90\%$
Test for subgroup differences: $Chi^2 = 105.28$, df = 11 ($P < 0.01$)

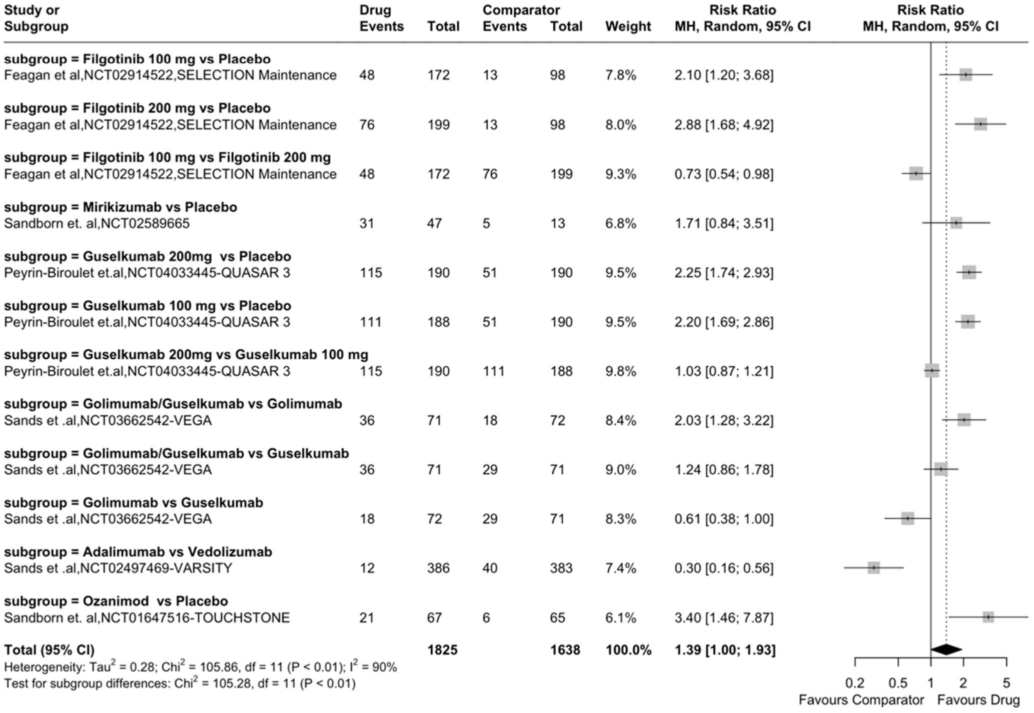

Supplementary Figure S20: pair-wise meta-analysis for induction of endoscopic improvement in biologic-naïve and no previous biologic failure status patients

Study or Subgroup	Drug Events	Total	Comparator Events	Total	Weight	Risk Ratio MH, Random, 95% CI
**subgroup = Etrasimod vs Placebo**						
Sandborn et. al,NCT03945188, ELEVATE UC 52	76	194	17	93	6.9%	2.14 [1.35; 3.41]
Sandborn et. al,NCT 03996369, ELEVATE UC 12	51	148	14	74	6.5%	1.82 [1.08; 3.07]
**Total (95% CI)**		**342**		**167**	**13.4%**	**1.99 [1.41; 2.82]**
Heterogeneity: $Tau^2 = 0$; $Chi^2 = 0.21$, df = 1 (P = 0.65); $I^2 = 0\%$						
**subgroup = Mirikizumab vs Placebo**						
Sandborn et. al,NCT02589665	11	22	2	23	2.5%	5.75 [1.43; 23.05]
Haens et.al,NCT03518086-LUCENT 1	226	492	48	171	8.2%	1.64 [1.26; 2.12]
**Total (95% CI)**		**514**		**194**	**10.6%**	**2.53 [0.78; 8.24]**
Heterogeneity: $Tau^2 = 0.54$; $Chi^2 = 3.07$, df = 1 (P = 0.08); $I^2 = 67\%$						
**subgroup = Upadacitinib vs Placebo**						
Sandborn et. al,NCT02819635, U-ACHIEVE-induction-SUBSTUDY 1	9	14	1	12	1.5%	7.71 [1.13; 52.45]
Sandborn et. al,NCT02819635, U-ACHIEVE-induction-SUBSTUDY 2	71	151	10	76	6.0%	3.57 [1.96; 6.52]
Sandborn et. al,NCT03653026-U-ACCOMPLISH	86	168	10	85	6.0%	4.35 [2.39; 7.93]
**Total (95% CI)**		**333**		**173**	**13.5%**	**4.07 [2.69; 6.16]**
Heterogeneity: $Tau^2 = 0$; $Chi^2 = 0.66$, df = 2 (P = 0.72); $I^2 = 0\%$						
**subgroup = Ustekinumab vs Placebo**						
Sands et .al,NCT02407236-UNIFI	52	156	33	158	7.5%	1.60 [1.10; 2.32]
**subgroup = Guselkumab vs Placebo**						
Peyrin-Biroulet et.al,NCT04033435-QUASAR 2b	20	55	8	54	5.2%	2.45 [1.18; 5.09]
**subgroup = Risankizumab vs Placebo**						
Louis et.al,NCT03398148-INSPIRE	151	317	22	155	7.3%	3.36 [2.24; 5.03]
**subgroup = Filgotinib 100 mg vs Placebo**						
Feagan et al,NCT02914522,SELECTION GROUP A	73	277	28	137	7.4%	1.29 [0.88; 1.89]
**subgroup = Filgotinib 200 mg vs Placebo**						
Feagan et al,NCT02914522,SELECTION GROUP A	83	245	28	137	7.5%	1.66 [1.14; 2.41]
**subgroup = Filgotinib 100 mg vs Filgotinib 200 mg**						
Feagan et al,NCT02914522,SELECTION GROUP A	73	277	83	245	8.1%	0.78 [0.60; 1.01]
**subgroup = Vedolizumab vs Placebo**						
Motoya et.al,NCT02039505	38	79	13	41	6.6%	1.52 [0.92; 2.51]
**subgroup = Tofacitinib vs Placebo**						
Sandborn et. al,NCT01465763-OCTAVE 1	88	222	15	57	6.9%	1.51 [0.95; 2.40]
Sandborn et. al,NCT01458951-OCTAVE 2	71	196	9	47	5.9%	1.89 [1.02; 3.50]
**Total (95% CI)**		**418**		**104**	**12.8%**	**1.64 [1.13; 2.37]**
Heterogeneity: $Tau^2 = 0$; $Chi^2 = 0.34$, df = 1 (P = 0.56); $I^2 = 0\%$						
**Total (95% CI)**		**3013**		**1565**	**100.0%**	**1.95 [1.51; 2.53]**
Heterogeneity: $Tau^2 = 0.19$; $Chi^2 = 71.69$, df = 15 (P < 0.01); $I^2 = 79\%$						
Test for subgroup differences: $Chi^2 = 65.83$, df = 10 (P < 0.01)						

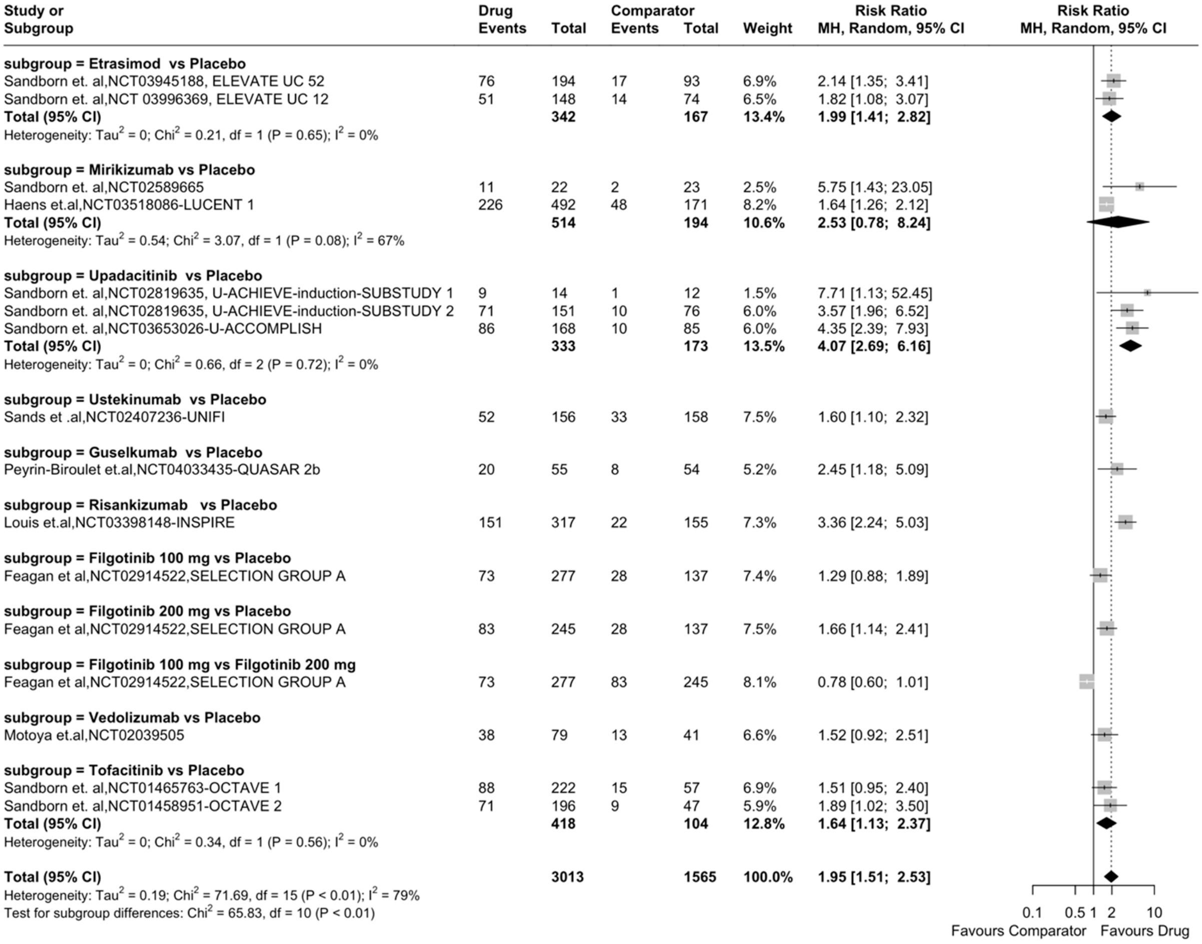

Supplementary Figure S21: pair-wise meta-analysis for induction of endoscopic improvement in biologic-exposed and with previous biologic failure status patients

Study or Subgroup	Drug Events	Drug Total	Comparator Events	Comparator Total	Weight	Risk Ratio MH, Random, 95% CI
**subgroup = Etrasimod vs Placebo**						
Sandborn et. al,NCT03945188, ELEVATE UC 52	20	80	2	42	4.4%	5.25 [1.29; 21.39]
Sandborn et. al,NCT 03996369, ELEVATE UC 12	17	74	7	38	6.9%	1.25 [0.57; 2.74]
**Total (95% CI)**		**154**		**80**	**11.3%**	**2.29 [0.53; 9.83]**
Heterogeneity: $Tau^2 = 0.80$; $Chi^2 = 3.36$, df = 1 (P = 0.07); $I^2 = 70\%$						
**subgroup = Mirikizumab vs Placebo**						
Sandborn et. al,NCT02589665	8	40	2	40	4.1%	4.00 [0.90; 17.68]
Haens et.al,NCT03518086-LUCENT 1	85	361	12	118	8.0%	2.32 [1.31; 4.08]
**Total (95% CI)**		**401**		**158**	**12.1%**	**2.48 [1.46; 4.22]**
Heterogeneity: $Tau^2 = 0$; $Chi^2 = 0.45$, df = 1 (P = 0.50); $I^2 = 0\%$						
**subgroup = Upadacitinib vs Placebo**						
Sandborn et. al,NCT02819635, U-ACHIEVE-induction-SUBSTUDY 1	11	42	0	34	1.5%	20.90 [1.09; 400.42]
Sandborn et. al,NCT02819635, U-ACHIEVE-induction-SUBSTUDY 2	45	168	1	78	2.9%	20.89 [2.93; 148.82]
Sandborn et. al,NCT03653026-U-ACCOMPLISH	64	173	4	89	6.1%	8.23 [3.10; 21.87]
**Total (95% CI)**		**383**		**201**	**10.4%**	**10.52 [4.55; 24.34]**
Heterogeneity: $Tau^2 = 0$; $Chi^2 = 0.97$, df = 2 (P = 0.62); $I^2 = 0\%$						
**subgroup = Ustekinumab vs Placebo**						
Sands et .al,NCT02407236-UNIFI	35	166	11	161	7.7%	3.09 [1.62; 5.86]
**subgroup = Guselkumab vs Placebo**						
Peyrin-Biroulet et.al,NCT04033435-QUASAR 2b	11	46	5	51	6.0%	2.44 [0.92; 6.49]
**subgroup = Risankizumab vs Placebo**						
Louis et.al,NCT03398148-INSPIRE	86	333	17	170	8.4%	2.58 [1.59; 4.20]
**subgroup = Filgotinib 100 mg vs Placebo**						
Feagan et al,NCT02914522,SELECTION GROUP B	37	285	11	142	7.7%	1.68 [0.88; 3.19]
**subgroup = Filgotinib 200 mg vs Placebo**						
Feagan et al,NCT02914522,SELECTION GROUP B	45	262	11	142	7.7%	2.22 [1.18; 4.15]
**subgroup = Filgotinib 100 mg vs Filgotinib 200 mg**						
Feagan et al,NCT02914522,SELECTION GROUP B	37	285	45	262	8.7%	0.76 [0.51; 1.13]
**subgroup = Vedolizumab vs Placebo**						
Motoya et.al,NCT02039505	22	85	12	41	7.9%	0.88 [0.49; 1.61]
**subgroup = Tofacitinib vs Placebo**						
Sandborn et. al,NCT01465763-OCTAVE 1	61	254	4	65	6.1%	3.90 [1.47; 10.34]
Sandborn et. al,NCT01458951-OCTAVE 2	51	234	4	65	6.0%	3.54 [1.33; 9.44]
**Total (95% CI)**		**488**		**130**	**12.1%**	**3.72 [1.86; 7.42]**
Heterogeneity: $Tau^2 = 0$; $Chi^2 = 0.02$, df = 1 (P = 0.89); $I^2 = 0\%$						
**Total (95% CI)**		**2888**		**1538**	**100.0%**	**2.50 [1.68; 3.72]**
Heterogeneity: $Tau^2 = 0.43$; $Chi^2 = 60.49$, df = 15 (P < 0.01); $I^2 = 75\%$						
Test for subgroup differences: $Chi^2 = 51.59$, df = 10 (P < 0.01)						

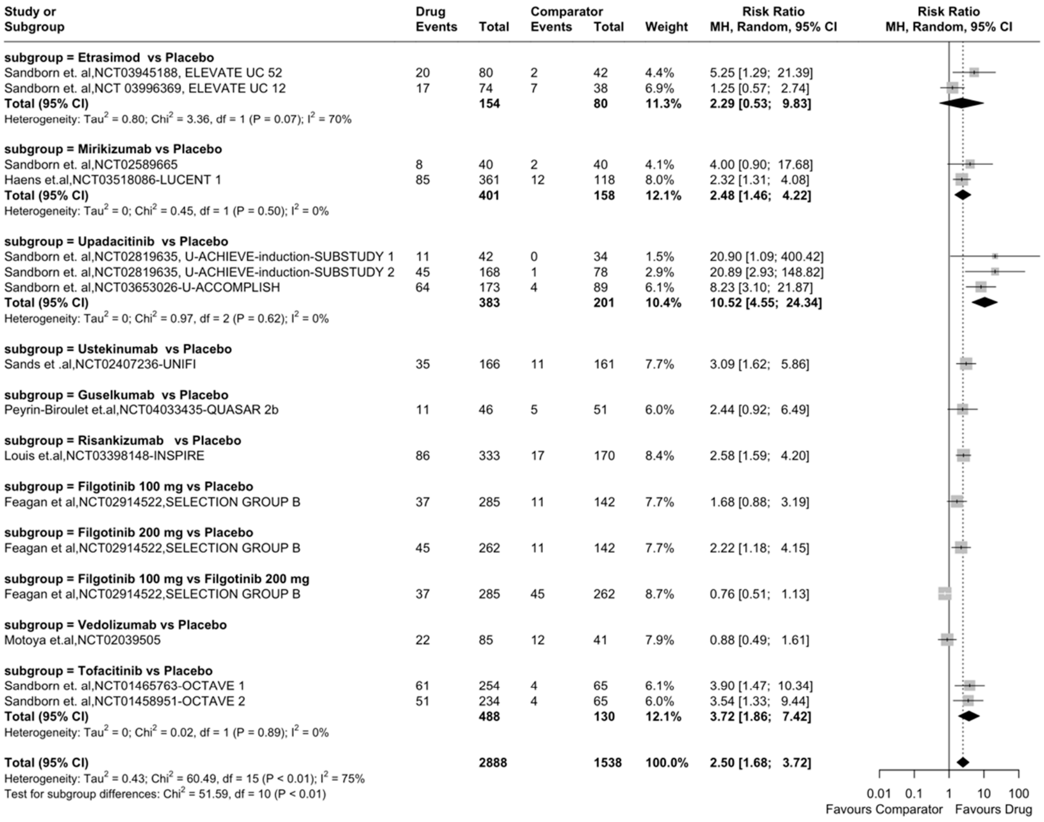

Supplementary Figure S22: pair-wise meta-analysis for maintenance of endoscopic improvement in biologic-naïve and no previous biologic failure status patients

Study or Subgroup	Drug Events	Total	Comparator Events	Total	Weight	Risk Ratio MH, Random, 95% CI
**subgroup = Etrasimod vs Placebo**						
Sandborn et. al,NCT03945188, ELEVATE UC 52	78	194	12	93	6.6%	3.12 [1.79; 5.43]
**subgroup = Mirikizumab vs Placebo**						
Haens et.al,NCT03518086-LUCENT 2	143	229	39	114	7.7%	1.83 [1.39; 2.40]
**subgroup = Upadacitinib 30mg vs Placebo**						
Sandborn et. al,NCT02819635, U-ACHIEVE-Maintenance-SUBSTUDY 3	54	81	15	68	7.0%	3.02 [1.88; 4.85]
**subgroup = Upadacitinib 15mg vs Placebo**						
Sandborn et. al,NCT02819635, U-ACHIEVE-Maintenance-SUBSTUDY 3	41	77	15	68	6.9%	2.41 [1.47; 3.95]
**subgroup = Upadacitinib 15 mg vs Upadacitinib 30 mg**						
Sandborn et. al,NCT02819635, U-ACHIEVE-Maintenance-SUBSTUDY 3	41	77	54	81	7.8%	0.80 [0.62; 1.04]
**subgroup = Ustekinumab vs Placebo**						
Sands et .al,NCT02407236-UNIFI	49	85	30	87	7.5%	1.67 [1.19; 2.35]
**subgroup = Risankizumab 180 mg SC vs Placebo**						
Louis et.al,NCT03398135-COMMAND	27	45	16	45	7.0%	1.69 [1.07; 2.67]
**subgroup = Risankizumab 360 mg vs Placebo**						
Louis et.al,NCT03398135-COMMAND	39	47	16	45	7.2%	2.33 [1.54; 3.53]
**subgroup = Risankizumab 180mg vs Risankizumab 360mg**						
Louis et.al,NCT03398135-COMMAND	27	45	39	47	7.7%	0.72 [0.55; 0.95]
**subgroup = Filgotinib 100 mg vs Placebo**						
Feagan et al,NCT02914522,SELECTION GROUP A	32	105	10	54	6.3%	1.65 [0.88; 3.09]
**subgroup = Filgotinib 200 mg vs Placebo**						
Feagan et al,NCT02914522,SELECTION GROUP A	57	107	10	54	6.5%	2.88 [1.60; 5.17]
**subgroup = Filgotinib 100 mg vs Filgotinib 200 mg**						
Feagan et al,NCT02914522,SELECTION GROUP A	32	105	57	107	7.5%	0.57 [0.41; 0.80]
**subgroup = Adalimumab vs Vedolizumab**						
Sands et .al,NCT02497469-VARSITY	90	305	131	304	7.9%	0.68 [0.55; 0.85]
**subgroup = Vedolizumab vs Placebo**						
Motoya et.al,NCT02039505	15	24	10	28	6.5%	1.75 [0.97; 3.14]
**Total (95% CI)**		**1526**		**1195**	**100.0%**	**1.52 [1.10; 2.10]**

Heterogeneity: $Tau^2 = 0.33$; $Chi^2 = 140.43$, df = 13 ($P < 0.01$); $I^2 = 91\%$
Test for subgroup differences: $Chi^2 = 136.17$, df = 13 ($P < 0.01$)

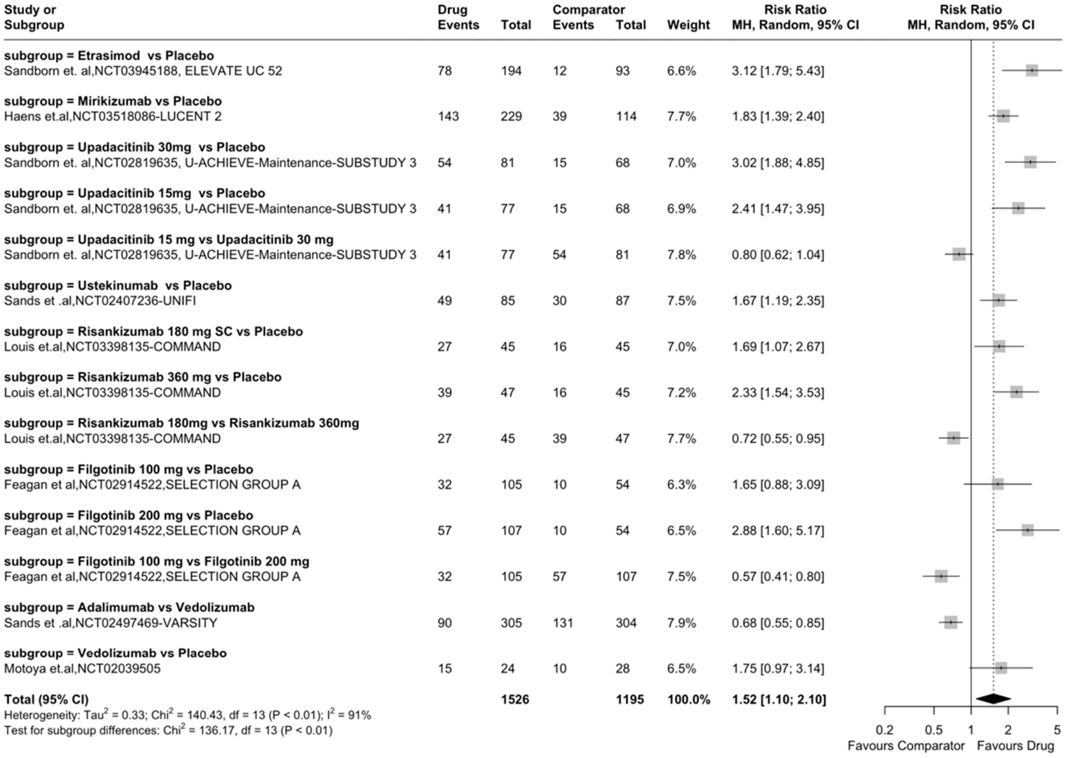

Supplementary Figure S23: pair-wise meta-analysis for maintenance of endoscopic improvement in biologic-exposed and with previous biologic failure status patients

Study or Subgroup	Drug Events	Total	Comparator Events	Total	Weight	Risk Ratio MH, Random, 95% CI
**subgroup = Etrasimod vs Placebo**						
Sandborn et. al,NCT03945188, ELEVATE UC 52	24	80	2	42	3.4%	6.30 [1.56; 25.38]
**subgroup = Mirikizumab vs Placebo**						
Haens et.al,NCT03518086-LUCENT 2	65	128	13	64	7.9%	2.50 [1.49; 4.18]
**subgroup = Upadacitinib 30mg vs Placebo**						
Sandborn et. al,NCT02819635, U-ACHIEVE-Maintenance-SUBSTUDY 3	41	73	6	81	6.1%	7.58 [3.42; 16.81]
**subgroup = Upadacitinib 15mg vs Placebo**						
Sandborn et. al,NCT02819635, U-ACHIEVE-Maintenance-SUBSTUDY 3	31	71	6	81	6.0%	5.89 [2.61; 13.30]
**subgroup = Upadacitinib 15 mg vs Upadacitinib 30 mg**						
Sandborn et. al,NCT02819635, U-ACHIEVE-Maintenance-SUBSTUDY 3	31	71	41	73	9.0%	0.78 [0.56; 1.08]
**subgroup = Ustekinumab vs Placebo**						
Sands et .al,NCT02407236-UNIFI	41	91	20	88	8.4%	1.98 [1.27; 3.10]
**subgroup = Risankizumab 180 mg SC vs Placebo**						
Louis et.al,NCT03398135-COMMAND	64	134	42	138	9.2%	1.57 [1.15; 2.14]
**subgroup = Risankizumab 360 mg vs Placebo**						
Louis et.al,NCT03398135-COMMAND	54	139	42	138	9.1%	1.28 [0.92; 1.77]
**subgroup = Risankizumab 180mg vs Risankizumab 360mg**						
Louis et.al,NCT03398135-COMMAND	64	134	54	139	9.4%	1.23 [0.94; 1.62]
**subgroup = Filgotinib 100 mg vs Placebo**						
Feagan et al,NCT02914522,SELECTION GROUP B	14	67	5	44	5.3%	1.84 [0.71; 4.74]
**subgroup = Filgotinib 200 mg vs Placebo**						
Feagan et al,NCT02914522,SELECTION GROUP B	24	92	5	44	5.6%	2.30 [0.94; 5.61]
**subgroup = Filgotinib 100 mg vs Filgotinib 200 mg**						
Feagan et al,NCT02914522,SELECTION GROUP B	14	67	24	92	7.5%	0.80 [0.45; 1.43]
**subgroup = Adalimumab vs Vedolizumab**						
Sands et .al,NCT02497469-VARSITY	17	81	21	79	7.6%	0.79 [0.45; 1.38]
**subgroup = Vedolizumab vs Placebo**						
Motoya et.al,NCT02039505	11	17	4	14	5.5%	2.26 [0.92; 5.57]
**Total (95% CI)**		**1245**		**1117**	**100.0%**	**1.77 [1.29; 2.43]**

Heterogeneity: $Tau^2 = 0.26$; $Chi^2 = 68.58$, df = 13 ($P < 0.01$); $I^2 = 81\%$
Test for subgroup differences: $Chi^2 = 64.79$, df = 13 ($P < 0.01$)

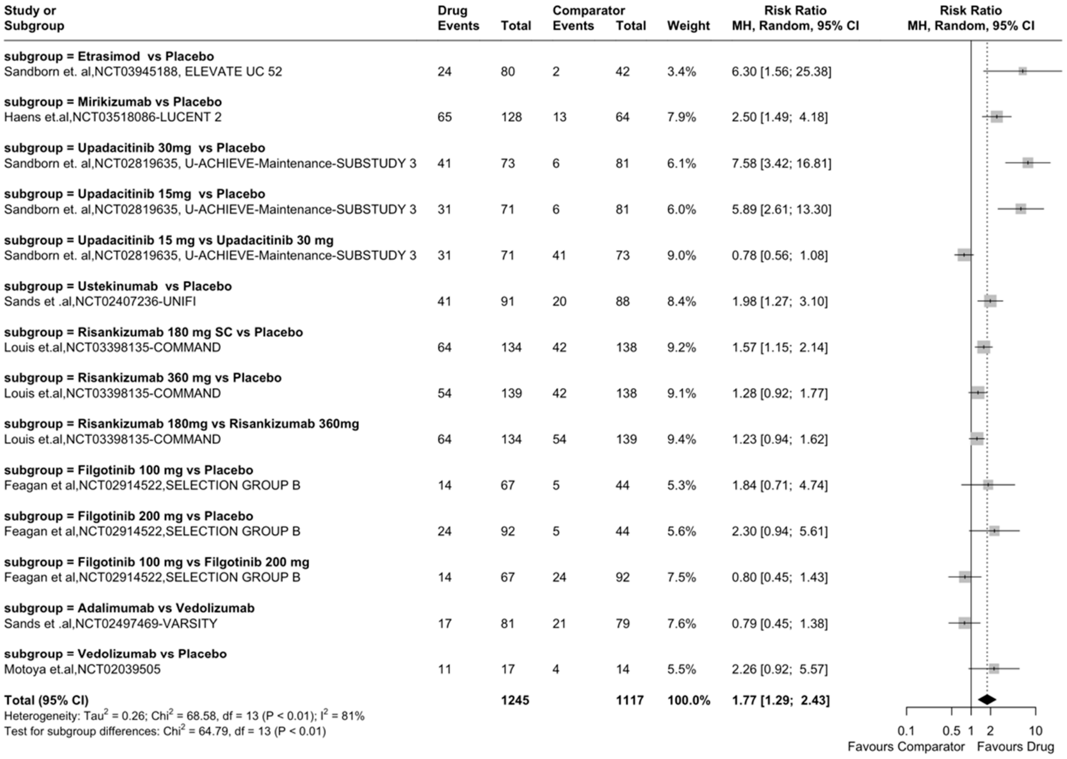

Supplementary Figure S24: pair-wise meta-analysis for induction of mucosal healing in biologic-naïve and no previous biologic failure status patients

Study or Subgroup	Drug Events	Total	Comparator Events	Total	Weight	Risk Ratio MH, Random, 95% CI
**subgroup = Etrasimod vs Placebo**						
Sandborn et. al,NCT03945188, ELEVATE UC 52	47	194	6	93	10.1%	3.76 [1.67; 8.47]
Sandborn et. al,NCT 03996369, ELEVATE UC 12	28	148	8	74	10.9%	1.75 [0.84; 3.65]
**Total (95% CI)**		**342**		**167**	**21.0%**	**2.51 [1.18; 5.35]**
Heterogeneity: $Tau^2 = 0.14$; $Chi^2 = 1.91$, df = 1 (P = 0.17); $I^2 = 48\%$						
**subgroup = Mirikizumab vs Placebo**						
Haens et.al,NCT03518086-LUCENT 1	176	492	32	171	14.7%	1.91 [1.37; 2.67]
**subgroup = Ustekinumab vs Placebo**						
Sands et .al,NCT02407236-UNIFI	36	156	22	158	13.4%	1.66 [1.02; 2.68]
**subgroup = Guselkumab vs Placebo**						
Peyrin-Biroulet et.al,NCT04033435-QUASAR 2b	15	55	6	54	9.6%	2.45 [1.03; 5.85]
**subgroup = Risankizumab vs Placebo**						
Louis et.al,NCT03398148-INSPIRE	34	317	0	155	1.4%	51.62 [1.67; 1595.61]
**subgroup = Filgotinib 100 mg vs Placebo**						
Feagan et al,NCT02914522,SELECTION GROUP A	41	277	15	137	12.6%	1.35 [0.78; 2.35]
**subgroup = Filgotinib 200 mg vs Placebo**						
Feagan et al,NCT02914522,SELECTION GROUP A	57	245	15	137	12.9%	2.12 [1.25; 3.61]
**subgroup = Filgotinib 100 mg vs Filgotinib 200 mg**						
Feagan et al,NCT02914522,SELECTION GROUP A	41	277	57	245	14.5%	0.64 [0.44; 0.91]
**Total (95% CI)**		**2161**		**1224**	**100.0%**	**1.76 [1.16; 2.69]**
Heterogeneity: $Tau^2 = 0.29$; $Chi^2 = 37.23$, df = 8 (P < 0.01); $I^2 = 79\%$						
Test for subgroup differences: $Chi^2 = 32.41$, df = 7 (P < 0.01)						

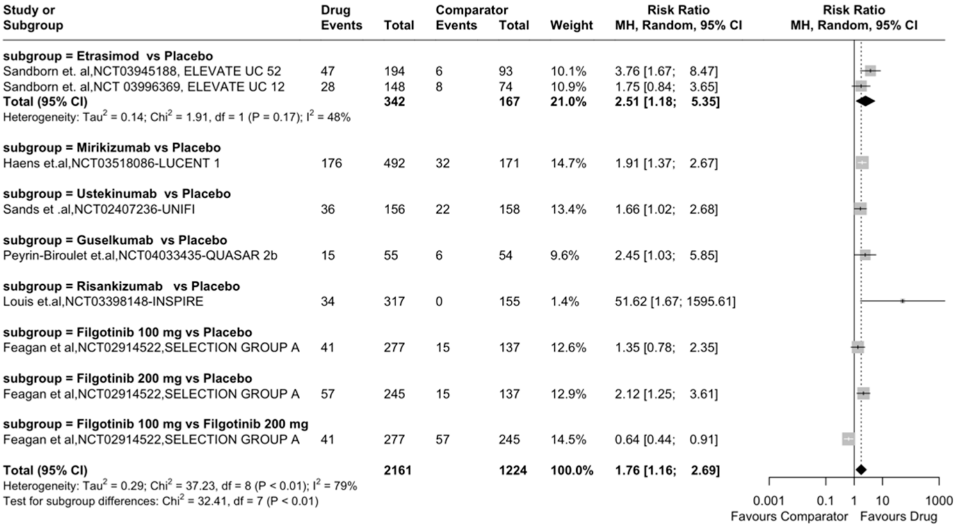

Supplementary Figure S25: pair-wise meta-analysis for induction of mucosal healing in biologic-exposed and with previous biologic failure status patients

Study or Subgroup	Drug Events	Total	Comparator Events	Total	Weight	Risk Ratio MH, Random, 95% CI
**subgroup = Etrasimod vs Placebo**						
Sandborn et. al,NCT03945188, ELEVATE UC 52	11	80	0	42	2.0%	17.78 [0.61; 516.32]
Sandborn et. al,NCT 03996369, ELEVATE UC 12	8	74	2	38	7.6%	2.05 [0.46; 9.20]
**Total (95% CI)**		**154**		**80**	**9.6%**	**3.88 [0.45; 33.26]**
Heterogeneity: $Tau^2 = 1.12$; $Chi^2 = 1.63$, df = 1 (P = 0.20); $I^2 = 39\%$						
**subgroup = Mirikizumab vs Placebo**						
Haens et.al,NCT03518086-LUCENT 1	56	361	8	118	15.9%	2.29 [1.12; 4.66]
**subgroup = Ustekinumab vs Placebo**						
Sands et .al,NCT02407236-UNIFI	22	166	6	161	13.6%	3.56 [1.48; 8.54]
**subgroup = Guselkumab vs Placebo**						
Peyrin-Biroulet et.al,NCT04033435-QUASAR 2b	6	46	3	51	8.9%	2.22 [0.59; 8.36]
**subgroup = Risankizumab vs Placebo**						
Louis et.al,NCT03398148-INSPIRE	7	333	2	170	7.2%	1.79 [0.38; 8.51]
**subgroup = Filgotinib 100 mg vs Placebo**						
Feagan et al,NCT02914522,SELECTION GROUP B	18	285	6	142	13.3%	1.49 [0.61; 3.68]
**subgroup = Filgotinib 200 mg vs Placebo**						
Feagan et al,NCT02914522,SELECTION GROUP B	26	262	6	142	13.8%	2.35 [0.99; 5.57]
**subgroup = Filgotinib 100 mg vs Filgotinib 200 mg**						
Feagan et al,NCT02914522,SELECTION GROUP B	18	285	26	262	17.8%	0.64 [0.36; 1.13]
**Total (95% CI)**		**1892**		**1126**	**100.0%**	**1.86 [1.12; 3.08]**
Heterogeneity: $Tau^2 = 0.28$; $Chi^2 = 17.38$, df = 8 (P = 0.03); $I^2 = 54\%$						
Test for subgroup differences: $Chi^2 = 15.64$, df = 7 (P = 0.03)						

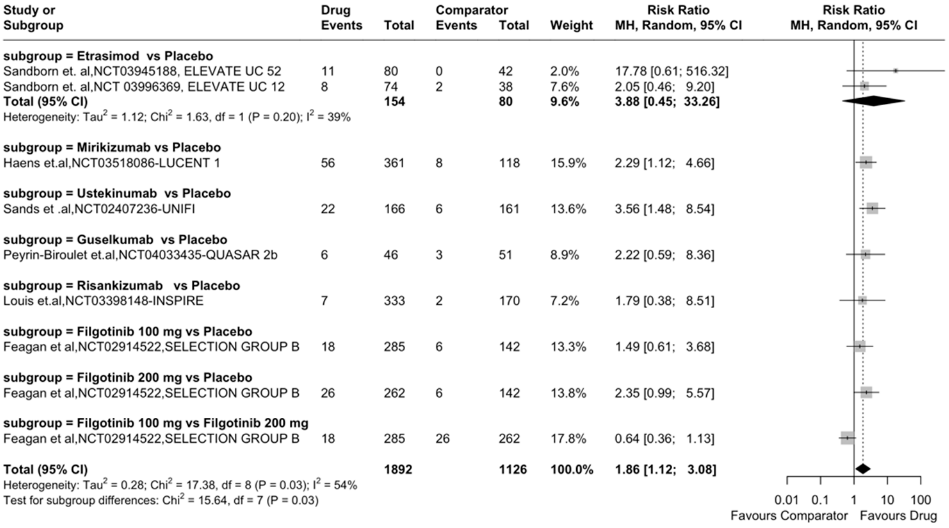

Supplementary Figure S24: pair-wise meta-analysis for maintenance of mucosal healing in biologic-naïve and no previous biologic failure status patients

Study or Subgroup	Drug Events	Drug Total	Comparator Events	Comparator Total	Weight	Risk Ratio MH, Random, 95% CI
**subgroup = Etrasimod vs Placebo**						
Sandborn et. al,NCT03945188, ELEVATE UC 52	55	194	10	93	11.3%	2.64 [1.41; 4.93]
**subgroup = Mirikizumab vs Placebo**						
Haens et.al,NCT03518086-LUCENT 2	108	229	30	114	18.8%	1.79 [1.28; 2.51]
**subgroup = Upadacitinib 30mg vs Placebo**						
Sandborn et. al,NCT02819635, U-ACHIEVE-Maintenance-SUBSTUDY 3	18	81	5	68	6.7%	3.02 [1.18; 7.71]
**subgroup = Upadacitinib 15 mg vs Placebo**						
Sandborn et. al,NCT02819635, U-ACHIEVE-Maintenance-SUBSTUDY 3	14	77	5	68	6.4%	2.47 [0.94; 6.51]
**subgroup = Upadacitinib 15 mg vs Upadacitinib 30 mg**						
Sandborn et. al,NCT02819635, U-ACHIEVE-Maintenance-SUBSTUDY 3	14	77	18	81	11.4%	0.82 [0.44; 1.53]
**subgroup = Risankizumab 180 mg SC vs Placebo**						
Louis et.al,NCT03398135-COMMAND	12	45	8	45	8.5%	1.50 [0.68; 3.32]
**subgroup = Risankizumab 360 mg vs Placebo**						
Louis et.al,NCT03398135-COMMAND	15	47	8	45	9.1%	1.80 [0.84; 3.82]
**subgroup = Risankizumab 180mg vs Risankizumab 360mg**						
Louis et.al,NCT03398135-COMMAND	12	45	15	47	11.1%	0.84 [0.44; 1.58]
**subgroup = CT-P13 vs Placebo**						
Sands et.al,NCT04205643-LIBERTY-UC	96	265	23	131	16.8%	2.06 [1.38; 3.09]
**Total (95% CI)**		**1060**		**692**	**100.0%**	**1.68 [1.27; 2.23]**

Heterogeneity: $Tau^2 = 0.08$; $Chi^2 = 15.13$, df = 8 (P = 0.06); $I^2 = 47\%$
Test for subgroup differences: $Chi^2 = 15.00$, df = 8 (P = 0.06)

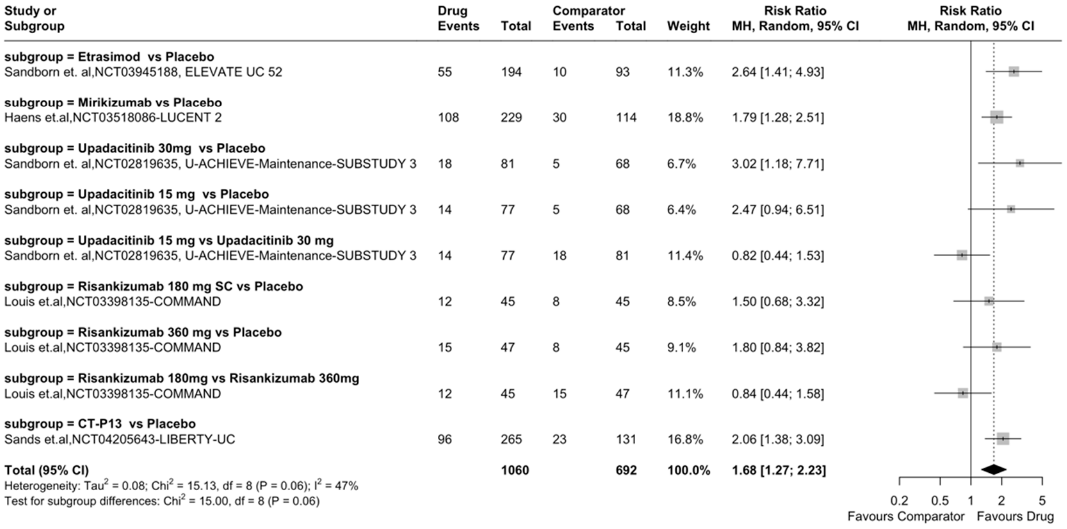

Supplementary Figure S25: pair-wise meta-analysis for maintenance of mucosal healing in biologic-exposed and with previous biologic failure status patients

Study or Subgroup	Drug Events	Total	Comparator Events	Total	Weight	Risk Ratio MH, Random, 95% CI
**subgroup = Etrasimod vs Placebo**						
Sandborn et. al,NCT03945188, ELEVATE UC 52	18	80	1	42	5.2%	9.45 [1.31; 68.36]
**subgroup = Mirikizumab vs Placebo**						
Haens et.al,NCT03518086-LUCENT 2	46	128	9	64	15.9%	2.56 [1.34; 4.89]
**subgroup = Upadacitinib 30mg vs Placebo**						
Sandborn et. al,NCT02819635, U-ACHIEVE-Maintenance-SUBSTUDY 3	12	73	2	81	7.9%	6.66 [1.54; 28.76]
**subgroup = Upadacitinib 15 mg vs Placebo**						
Sandborn et. al,NCT02819635, U-ACHIEVE-Maintenance-SUBSTUDY 3	12	71	2	81	7.9%	6.85 [1.59; 29.55]
**subgroup = Upadacitinib 15 mg vs Upadacitinib 30 mg**						
Sandborn et. al,NCT02819635, U-ACHIEVE-Maintenance-SUBSTUDY 3	12	71	12	73	14.9%	1.03 [0.50; 2.13]
**subgroup = Risankizumab 180 mg SC vs Placebo**						
Louis et.al,NCT03398135-COMMAND	11	134	10	138	13.8%	1.13 [0.50; 2.58]
**subgroup = Risankizumab 360 mg vs Placebo**						
Louis et.al,NCT03398135-COMMAND	14	139	10	138	14.4%	1.39 [0.64; 3.02]
**subgroup = Risankizumab 180mg vs Risankizumab 360mg**						
Louis et.al,NCT03398135-COMMAND	11	134	14	139	14.6%	0.82 [0.38; 1.73]
**subgroup = CT-P13 vs Placebo**						
Sands et.al,NCT04205643-LIBERTY-UC	9	29	1	13	5.3%	4.03 [0.57; 28.63]
**Total (95% CI)**		**859**		**769**	**100.0%**	**1.98 [1.17; 3.33]**

Heterogeneity: $Tau^2 = 0.33$; $Chi^2 = 19.47$, df = 8 (P = 0.01); $I^2 = 59\%$
Test for subgroup differences: $Chi^2 = 18.63$, df = 8 (P = 0.02)

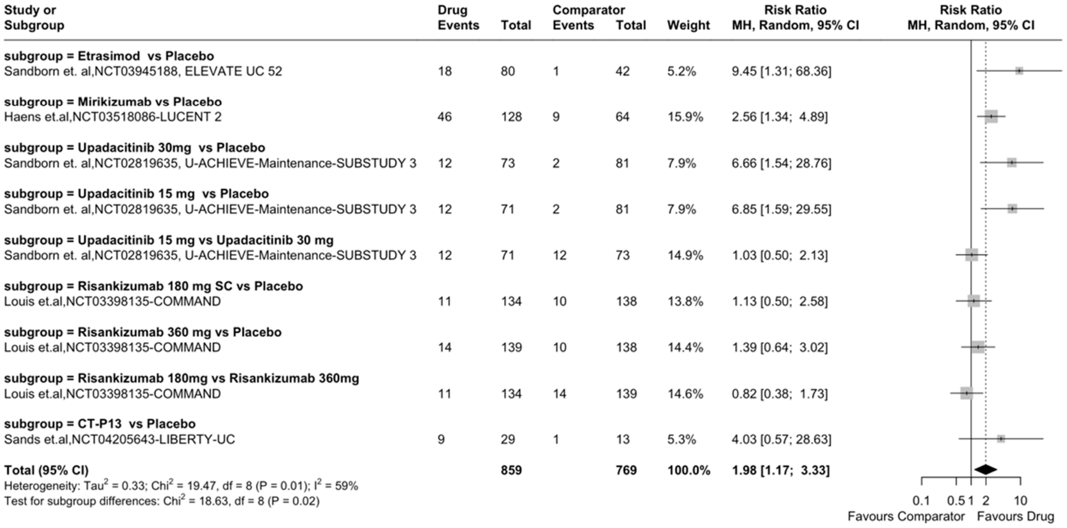

Supplementary Table S4: PRISMA checklist

**TITLE**			
Title	1	Identify the report as a systematic review.	Page-1
**ABSTRACT**			
Abstract	2	See the PRISMA 2020 for Abstracts checklist.	Page-1
**INTRODUCTION**			
Rationale	3	Describe the rationale for the review in the context of existing knowledge.	Page-2,3
Objectives	4	Provide an explicit statement of the objective(s) or question(s) the review addresses.	Page-2,3
**METHODS**			
Eligibility criteria	5	Specify the inclusion and exclusion criteria for the review and how studies were grouped for the syntheses.	Page-3
Information sources	6	Specify all databases, registers, websites, organisations, reference lists and other sources searched or consulted to identify studies. Specify the date when each source was last searched or consulted.	Page-3
Search strategy	7	Present the full search strategies for all databases, registers and websites, including any filters and limits used.	Supplementary Appendix page 44-49
Selection process	8	Specify the methods used to decide whether a study met the inclusion criteria of the review, including how many reviewers screened each record and each report retrieved, whether they worked independently, and if applicable, details of automation tools used in the process.	Page 3
Data collection process	9	Specify the methods used to collect data from reports, including how many reviewers collected data from each report, whether they worked independently, any processes for obtaining or confirming data from study investigators, and if applicable, details of automation tools used in the process.	Page 3
Data items	10a	List and define all outcomes for which data were sought. Specify whether all results that were compatible with each outcome domain in each study were sought (e.g. for all measures, time points, analyses), and if not, the methods used to decide which results to collect.	Page 3,4
	10b	List and define all other variables for which data were sought (e.g. participant and intervention characteristics, funding sources). Describe any assumptions made about any missing or unclear information.	Supplementary Appendix page 3-15
Study risk of bias assessment	11	Specify the methods used to assess risk of bias in the included studies, including details of the tool(s) used, how many reviewers assessed each study and whether they worked independently, and if applicable, details of automation tools used in the process.	Page 3
Effect measures	12	Specify for each outcome the effect measure(s) (e.g. risk ratio, mean difference) used in the synthesis or presentation of results.	Page 4
Synthesis methods	13a	Describe the processes used to decide which studies were eligible for each synthesis (e.g. tabulating the study intervention characteristics and comparing against the planned groups for each synthesis (item #5)).	Page 3,4
	13b	Describe any methods required to prepare the data for presentation or synthesis, such as handling of missing summary statistics, or data conversions.	NA
	13c	Describe any methods used to tabulate or visually display results of individual studies and syntheses.	NA
	13d	Describe any methods used to synthesize results and provide a rationale for the choice(s). If meta-analysis was performed, describe the model(s), method(s) to identify the presence and extent of statistical heterogeneity, and software package(s) used.	Page 4
	13e	Describe any methods used to explore possible causes of heterogeneity among study results (e.g. subgroup analysis, meta-regression).	Page 4
	13f	Describe any sensitivity analyses conducted to assess robustness of the synthesized results.	Page 4
Reporting bias assessment	14	Describe any methods used to assess risk of bias due to missing results in a synthesis (arising from reporting biases).	Page 4
Certainty assessment	15	Describe any methods used to assess certainty (or confidence) in the body of evidence for an outcome.	Page 4

Supplementary Table S4:
PRISMA checklist

**RESULTS**			
Study selection	16a	Describe the results of the search and selection process, from the number of records identified in the search to the number of studies included in the review, ideally using a flow diagram.	Page 5
	16b	Cite studies that might appear to meet the inclusion criteria, but which were excluded, and explain why they were excluded.	Page 5
Study characteristics	17	Cite each included study and present its characteristics.	Supplementary Tables S1 and S2
Risk of bias in studies	18	Present assessments of risk of bias for each included study.	Supplementary Figure S1
Results of individual studies	19	For all outcomes, present, for each study: (a) summary statistics for each group (where appropriate) and (b) an effect estimate and its precision (e.g. confidence/credible interval), ideally using structured tables or plots.	Table 1,2, Supplementary Figures S3-S25
Results of syntheses	20a	For each synthesis, briefly summarise the characteristics and risk of bias among contributing studies.	NA
	20b	Present results of all statistical syntheses conducted. If meta-analysis was done, present for each the summary estimate and its precision (e.g. confidence/credible interval) and measures of statistical heterogeneity. If comparing groups, describe the direction of the effect.	Table 1,2, Supplementary Figures S3-S25
	20c	Present results of all investigations of possible causes of heterogeneity among study results.	NA
	20d	Present results of all sensitivity analyses conducted to assess the robustness of the synthesized results.	NA
Reporting biases	21	Present assessments of risk of bias due to missing results (arising from reporting biases) for each synthesis assessed.	NA
Certainty of evidence	22	Present assessments of certainty (or confidence) in the body of evidence for each outcome assessed.	Supplementary Table S3
**DISCUSSION**			
Discussion	23a	Provide a general interpretation of the results in the context of other evidence.	Page 10
	23b	Discuss any limitations of the evidence included in the review.	Page 11-12
	23c	Discuss any limitations of the review processes used.	Page 11-12
	23d	Discuss implications of the results for practice, policy, and future research.	Page 11
**OTHER INFORMATION**			
Registration and protocol	24a	Provide registration information for the review, including register name and registration number, or state that the review was not registered.	Page 4
	24b	Indicate where the review protocol can be accessed, or state that a protocol was not prepared.	Page 4
	24c	Describe and explain any amendments to information provided at registration or in the protocol.	NA
Support	25	Describe sources of financial or non-financial support for the review, and the role of the funders or sponsors in the review.	Page 12
Competing interests	26	Declare any competing interests of review authors.	Page 12
Availability of data, code and other materials	27	Report which of the following are publicly available and where they can be found: template data collection forms; data extracted from included studies; data used for all analyses; analytic code; any other materials used in the review.	Page 12

Search Algorithm

PubMed via Medline -1582

1 Tumor necrosis factor inhibitor*[tw]

2 Tumour necrosis factor inhibitor*[tw]

3 "TNF-alpha antagonist*"[tw]

4 "Tumor Necrosis Factor Alpha Blocking Agent*" [tw]

5 "Tumour Necrosis Factor Alpha Blocking Agent*" [tw]

6 TNF inhibitor* [tw]

7 TNF-a blocker*[tw]

8 "anti-TNF agent*" [tw]

9 Infliximab [tw]

10 Remicade [tw]

11 Inflectra [tw]

12 CT-P 13 [tw]

13 Adalimumab [tw]

14 Humira [tw]

15 Golimumab [tw]

16 Simponi [tw]

17 Vedolizumab [tw]

18 Entyvio [tw]

19 Tofacitinib [tw]

20 Xeljanz [tw]

21 ustekinumab [tw]

22 Stelara [tw]

23 etrolizumab [tw]

24 filgotinib [tw]

25 ozanimod [tw]

26 *upadacitinib* [tw]

27 etrasimod

28 mirikizumab

29 jak inhibitor* [tw]

30 anti-integrin [tw]

31 Anti-Interleukin [tw]

32 Guselkumab[tw]

33 Tremfya [tw]

34 Risankizumab[tw]

35 Skyrizi [tw]

36 OR/1-35

37 Ulcerative colitis [mh]

38 Proctocolitis [mh]

39 Proctitis [mh]

40 Pancolitis [tw]

41 rectitis [tw]

42 proctocolitis [tw]

43 procto-colitis [tw]

44 colorectitis [tw]

45 rectocolitis [tw]

46 recto-sigmoiditis [tw]

47 rectosigmoiditis [tw]

48 procto-sigmoiditis [tw]

49 proctosigmoiditis [tw]

50 proctitis [tw]

51 ((Ulcer* or total or sub-total or subtotal or extensive or left-sided or universal) AND colitis) [tw]

52 OR/ 37-51

53 Randomised controlled trial [mh]

54 randomized controlled trial [mh]

55 Double Blind Method [mh]

56 Single Blind Method [mh]

57 Random Allocation [mh]

58 clinical trial [mh]

59 ((singl* or doub* or treb* or tripl*) AND (blind or mask) [tw]

60 clinical trial phase i [tw]

61 clinical trial phase ii [tw]

62 clinical trial phase iii [tw]

63 clinical trial phase iv [tw]

64 controlled clinical trial [tw]

65 randomized controlled trial [tw]

66 randomised controlled trial [tw]

67 multicenter study [tw]

68 clinical trial [tw]

69 random* [tw]

70 randomly allocated [tw]

71 OR/53-70

72 36 AND 52 AND 71

Embase via Ovid-2793 search until 27/04/2024 due to no access

1 TNF-alpha antagonist$.mp.

2 TNF inhibitor$.mp

3 TNF-a blocker$.mp

4 Infliximab.mp

5 Remicade.mp.

6 Inflectra .mp.

7 CT-P 13.mp

8 Adalimumab .mp.

9 Humira.mp.

10 Golimumab.mp.

11 Simponi.mp.

12 Vedolizumab.mp.

13 Entyvio.mp.

14 Tofacitinib .mp.

15 Xeljanz .mp.

16 ustekinumab .mp.

17 Stelara.mp.

18 etrolizumab.mp.

19 filgotinib.mp.

20 ozanimod.mp.

21 *upadacitinib*.mp.

22 jak inhibitor$.mp

23 anti-integrin.mp

24 Anti-Interleukin.mp

25 OR/1-24

26 ulcerative colitis.mp or ulcerative colitis/

27 proctocolitis.mp or proctocolitis/

28 proctosigmoiditis .mp.

29 procto-sigmoiditis .mp.

30 rectosigmoiditis .mp.

31 recto-sigmoiditis .mp.

32 rectocolitis.mp.

33 colorectitis .mp.

34 procto-colitis .mp.

35 proctocolitis .mp.

36 rectitis .mp.

37 OR/ 26-36

38 Randomised controlled trial/

39 Single blind procedure/

40 Double blind procedure/

41 Randomi?ed controlled trial$.mp.

42 Rct. mp.

43 Random allocation.mp

44 Single blind$.mp

45 Double blind$.mp

46 Triple blind$.mp

47 OR/38-46

49 25 AND 37 AND 47

CENTRAL -Cochrane Library-1072

1 Tumour necrosis factor inhibitor

2 TNF-alpha antagonist

3 TNF inhibitor

4 TNF-a blocker

5 Infliximab

6 Remicade

7 Inflectra

8 CT-P 13

9 Adalimumab

10 Humira

11 Golimumab

12 Simponi

13 Vedolizumab

14 Entyvio

15 Tofacitinib

16 Xeljanz

17 ustekinumab

18 Stelara

19 etrolizumab

20 filgotinib

21 ozanimod

22 *upadacitinib*

23 jak inhibitor

24 anti-integrin

25 Anti-Interleukin

26 Risankizumab

27 Skyrizi

28 Guselkumab

29 Tremfya

30 OR/1-29

31 MeSH descriptor: [Colitis, Ulcerative] explode all trees

32 MeSH descriptor [Proctitis] explode all trees

33 (total or sub-total or subtotal or extensive or left-sided or universal) AND colitis

34 Pancolitis or rectitis or proctocolitis or procto-colitis or colorectitis or rectocolitis or recto-sigmoiditis or rectosigmoiditis or procto-sigmoiditis or proctosigmoiditis or proctitis
35 OR/ 31-34
36 30 AND 35 in Cochrane Reviews, Cochrane Protocols and Trials

WEB OF SCIENCE-1947

#1 ALL=((Ulcerative colitis OR Proctocolitis OR Proctitis OR Pancolitis OR rectitis OR proctocolitis OR "procto-colitis" OR colorectitis OR rectocolitis OR "recto-sigmoiditis" OR rectosigmoiditis OR "procto-sigmoiditis" OR proctosigmoiditis OR proctitis OR (Ulcer* AND (total OR sub-total OR subtotal OR extensive OR left-sided OR universal) AND colitis)))

#2 ALL=((Randomised controlled trial OR randomized controlled trial OR "Double Blind Method" OR "Single Blind Method" OR "Random Allocation" OR "clinical trial" OR (singl* OR doub* OR treb* OR tripl*) AND (blind OR mask) OR "clinical trial phase i" OR "clinical trial phase ii" OR "clinical trial phase iii" OR "clinical trial phase iv" OR "controlled clinical trial" OR "randomized controlled trial" OR "randomised controlled trial" OR "multicenter study" OR random* OR "randomly allocated"))

#3 ALL=((Tumor necrosis factor inhibitor* OR Tumour necrosis factor inhibitor* OR "TNF-alpha antagonist*" OR "Tumor Necrosis Factor Alpha Blocking Agent*" OR "Tumour Necrosis Factor Alpha Blocking Agent*" OR TNF inhibitor* OR "TNF-a blocker*" OR "anti-TNF agent*" OR Infliximab OR Remicade OR Inflectra OR "CT-P 13" OR Adalimumab OR Humira OR Golimumab OR Simponi OR Vedolizumab OR Entyvio OR Tofacitinib OR Xeljanz OR ustekinumab OR Stelara OR etrolizumab OR filgotinib OR ozanimod OR upadacitinib OR "jak inhibitor*" OR "Risankizumab" OR "Guselkumab" "anti-integrin" OR "Anti-Interleukin"))
